# Targeting the overexpressed mitochondrial protein VDAC1 in a mouse model of Alzheimer’s disease protects against mitochondrial dysfunction and mitigates brain pathology

**DOI:** 10.1186/s40035-022-00329-7

**Published:** 2022-12-28

**Authors:** Ankit Verma, Anna Shteinfer-Kuzmine, Nikita Kamenetsky, Srinivas Pittala, Avijit Paul, Edna Nahon Crystal, Alberto Ouro, Vered Chalifa-Caspi, Swaroop Kumar Pandey, Alon Monsengo, Noga Vardi, Shira Knafo, Varda Shoshan-Barmatz

**Affiliations:** 1 Department of Life Sciences, Ben-Gurion University of the Negev, 84105 Beer-Sheva, Israel; 2 National Institute for Biotechnology in the Negev, Ben-Gurion University of the Negev, 84105 Beer-Sheva, Israel; 3 Achva Academic College, 79804 Shikmim, Israel; 4 Department of Physiology, Faculty of Health Sciences, Ben-Gurion University of the Negev, 84105 Beer-Sheva, Israel; 5 Ilse Katz Institute for Nanoscale Science and Technology, Ben-Gurion University of the Negev, 84105 Beer-Sheva, Israel; 6 The Shraga Segal Department of Microbiology, Immunology, and Genetics, Faculty of Health Sciences, Ben-Gurion University of the Negev, 84105 Beer-Sheva, Israel; 7 Present Address: NeuroAging Group (NEURAL), Clinical Neurosciences Research Laboratory (LINC), Health Research Institute of Santiago de Compostela (IDIS), 15706 Santiago de Compostela, Spain

**Keywords:** Alzheimer’s disease, Metabolism, Mitochondria, Neuroinflammation, VDAC1

## Abstract

**Background:**

Alzheimer's disease (AD) exhibits mitochondrial dysfunctions associated with dysregulated metabolism, brain inflammation, synaptic loss, and neuronal cell death. As a key protein serving as the mitochondrial gatekeeper, the voltage-dependent anion channel-1 (VDAC1) that controls metabolism and Ca^2+^ homeostasis is positioned at a convergence point for various cell survival and death signals. Here, we targeted VDAC1 with VBIT-4, a newly developed inhibitor of VDAC1 that prevents its pro-apoptotic activity, and mitochondria dysfunction.

**Methods:**

To address the multiple pathways involved in AD, neuronal cultures and a 5 × FAD mouse model of AD were treated with VBIT-4. We addressed multiple topics related to the disease and its molecular mechanisms using immunoblotting, immunofluorescence, q-RT-PCR, 3-D structural analysis and several behavioral tests.

**Results:**

In neuronal cultures, amyloid-beta (Aβ)-induced VDAC1 and p53 overexpression and apoptotic cell death were prevented by VBIT-4. Using an AD-like 5 × FAD mouse model, we showed that VDAC1 was overexpressed in neurons surrounding Aβ plaques, but not in astrocytes and microglia, and this was associated with neuronal cell death. VBIT-4 prevented the associated pathophysiological changes including neuronal cell death, neuroinflammation, and neuro-metabolic dysfunctions. VBIT-4 also switched astrocytes and microglia from being pro-inflammatory/neurotoxic to neuroprotective phenotype. Moreover, VBIT-4 prevented cognitive decline in the 5 × FAD mice as evaluated using several behavioral assessments of cognitive function. Interestingly, VBIT-4 protected against AD pathology, with no significant change in phosphorylated Tau and only a slight decrease in Aβ-plaque load.

**Conclusions:**

The study suggests that mitochondrial dysfunction with its gatekeeper VDAC1 is a promising target for AD therapeutic intervention, and VBIT-4 is a promising drug candidate for AD treatment.

**Supplementary Information:**

The online version contains supplementary material available at 10.1186/s40035-022-00329-7.

## Introduction

Alzheimer's disease (AD), a progressive neurodegenerative disorder characterized by severe memory impairment and cognitive deficits, currently affects up to 50 million people worldwide. There are expected 10 million new diagnoses every year, and this figure will reach over 135 million by 2050 [1]. The major neuropathological features of AD are synaptic malfunction, neuronal degeneration, accumulation of amyloid-beta peptide (Aβ) and phosphorylated Tau (p-Tau) [2]. For years, the amyloid cascade hypothesis dominated the field, suggesting that Aβ causes AD, with the expectation that eliminating Aβ or inhibiting its formation or aggregation could prevent or slow down the disease. However, targeting Aβ showed little beneficial effects on the AD pathology, and most clinical trials largely failed in improving AD conditions [3]. Accordingly, other non-Aβ and non-p-Tau mechanisms for AD development, such as lipid metabolism, neuroinflammation, and mitochondrial dysfunction have been proposed [4].

Substantial evidence has suggested that impaired brain metabolism in AD patients develops several decades before dementia appears [5], with brain hypometabolism preceding clinical signs of AD [6, 7], and is correlated with deteriorating cognitive function [8]. At least 25% of the body’s glucose is used to maintain the basal metabolic rate of the brain. In AD brains, the cerebral metabolic rate of glucose utilization is reduced [9]. In sporadic AD patients, hypometabolism of glucose occurs and ATP production declines to 50% of healthy individuals and continues to decline with disease progression [10]. Thus, AD is now considered a consequence of neuro-metabolic dysfunctions that lead to neurodegeneration and Aβ deposition [11].

The mitochondrial cascade hypothesis argues that the bioenergetic dysfunction mediates AD [12]. Early mitochondrial dysfunction in AD pathogenesis involves reduced metabolism, disrupted Ca^2+^-homeostasis, increased reactive oxygen radical (ROS) production, reduced mitochondrial DNA (mtDNA), altered mitochondrial morphology, reduced mitochondrial axonal transport [13], and activation of pro-apoptotic processes [14, 15].

Neuronal loss in the AD brain contributes to the progressive decline of memory and cognitive functions. While some neuronal loss is due to necrosis, most of it is due to apoptosis, with neurons in the AD brain displaying the hallmark of apoptosis [11]. Mitochondria-mediated cell death is implicated in premature neuronal death, with caspase-mediated apoptosis playing a dominant role [16, 17]. Moreover, Aβ directly acts on mitochondrial respiration, ATP synthesis, and metabolic enzyme activity, and it activates cytochrome *c* (Cyto *c*) release, leading to apoptosis [18]. Finally, evidence from “cybrid” technology strikingly showed that when mitochondria from individuals with AD are placed into cells from healthy individuals whose endogenous mitochondria have been removed, the generated cybrid cells display most of the cellular pathology of AD individuals [19]. Thus, targeting mitochondrial dysfunction associated with neurodegenerative disorders could be an effective therapeutic approach [20].

Here, we investigate the involvement of the mitochondrial gatekeeper—voltage-dependent anion channel-1 (VDAC1)—in AD pathology and as a potential therapeutic target. There are three isoforms of VDAC known to be expressed in mammals, VDAC1, VDAC2 and VDAC3. Of the three isoforms, VDAC1 is the most abundant protein with multiple functions that include transport of metabolites, fatty-acids, and Ca^2+^. It mediates the cross-talk between mitochondria and endoplasmic reticulum, and is involved in inflammasome activation [21, 22]. Additionally, VDAC1 is a hub protein that interacts with proteins that regulate the integration of mitochondrial functions with other cellular activities [21–24]. Importantly, VDAC1 is a key protein in mitochondria-mediated apoptosis. Stress conditions and apoptosis inducers lead to VDAC1 overexpression and oligomerization, forming a large channel that enables the release of pro-apoptotic proteins to the cytosol, and by interacting with apoptosis regulatory proteins such as Bcl-2, Bcl-xL, and hexokinase (HK), VDAC1 can switch from promoting vital metabolic process to promoting apoptosis [21–23, 25]. VDAC1 oligomers also allow the release of mtDNA [26, 27].

Accumulating evidence indicates that VDAC1 is involved in AD pathogenesis. It directly interacts with Aβ and p-Tau and is required for Aβ entry into the cell, leading to mitochondrial dysfunction and apoptosis [28–31]. High levels of VDAC1 have been demonstrated in brains of post-mortem AD patients, in AD-like transgenic mice [31, 32], and in other neurodegenerative disease models [33]. VDAC1 overexpression is associated with apoptotic cell death [34], leading to VDAC1 oligomerization, thereby to apoptosis [24, 25, 35, 36] and inflammation [26, 27].

VDAC1 overexpression, its oligomerization and apoptosis induction have been implicated in different diseases [37]. In cancers, VDAC1 is overexpressed [38–40], while its oligomerization and, thereby, apoptosis are prevented by the overexpression of anti-apoptotic proteins such as hexokinase and Bcl2, and their detachment from VDAC1 leads to VDAC1 oligomerization and apoptosis [22, 37, 39].

We have previously developed two VDAC1-interacting molecules, VBIT-4 and VBIT-12, that prevent VDAC1 oligomerization, proapoptotic protein release, ROS production and increase in intracellular Ca^2+^, leading to apoptosis inhibition, while having no effect on cells under physiological conditions [41]. Moreover, we have shown that VBIT-4 or VBIT-12 prevents mitochondrial dysfunction and apoptosis in several mouse models of diseases that show VDAC1 overexpression, such as type 2 diabetes (T2D) [42], lupus [26], and colitis [43].

This study was  aimed to explore the involvement of VDAC1 in AD pathology and the effects of the VDAC1-interacting molecule, VBIT-4, in preventing mitochondrial dysfunction and neuronal loss and restoring cognitive activity in a mouse model of AD.

## Methods

### Cell cultures, cell viability and apoptosis assays, VBIT-4 treatment, determination of VDAC1 expression levels, VDAC1 oligomerization, and VBIT-4 and VBIT-12 development

All are described in the Additional file 1.

### Immunoblotting, terminal deoxynucleotidyl transferase-mediated dUTP nick end labeling (TUNEL), Thioflavin-S Aβ staining, and q-RT-PCR analyses of brain

Immunostaining, TUNEL assay for apoptotic cell death, Thioflavin-S Aβ staining and q-RT-PCR using specific primers (Additional file 1: Table S1) were carried out as described in the Additional file 1.

### Primary culture of rat cortical neurons

Primary neuronal cultures were prepared from the cerebral cortex of E18.5 Sprague–Dawley rat embryos, as previously reported [44]. Cerebral cortices were dissociated in an HBSS dissection buffer (7 mM Hepes and 0.45% glucose without calcium and magnesium) including 0.05% trypsin and 1 mg/ml DNAse I. Cells were plated at a density of 2.5 × 10^5^ cells/cm^2^ on poly-*L*-lysine-coated coverslips placed inside the wells of a 24-well plate in neurobasal medium supplemented with B27 and GlutaMax and grown at 37 °C in 5% CO_2_ and 9% O_2_ for 5 days. Next, neurons were maintained with neurobasal medium supplemented with B27 for 12 days, and then were infected with APP swe/lnd-EGFP sinbis virus (Addgene, Watertown, MA) for 16 h. The expressed GFP represents infected cells. At 16 h post-infection, cells were collected and filtered with a 0.22-micron filter, then diluted 1:1 with neurobasal B27 medium, and used as a conditioned medium. To test the effect of the conditioned medium and the expression of VDAC1, p53 and activated caspase-3, the cortical neurons at 14 days in vitro (DIV14) were treated with or without the diluted conditioned medium in the presence or absence of VBIT-4 (10 μM) for 6, 24, and 48 h. Cells were fixed with 4% formaldehyde for 20 min, and analyzed for the expression of the above proteins using immunofluorescence (IF) staining and specific antibodies (Additional file 1: Table S2).

### Mice and treatment with VBIT-4 or VBIT-12

Male 5 × FAD transgenic mice were obtained from the Jackson Laboratory (Bar Harbor, ME) and crossed with C57Bl/RCC female mice. The 5 × FAD transgenic mice have five familial AD mutations, including amyloid precursor protein (*APP*) with K670N/M671L (Swedish mutation), I716V (Florida mutation), and V717I (London mutation), and presenilin 1 (*PS1*) with M146L and L286V mutations ([5 × FAD B6.Cg-TgAPPSwFlLon, PSEN1*M146Ln *L286V6799 Vas/J]). These mice develop massive cerebral Aβ42 loads, memory deficits, and neuronal loss [45]. Male offspring were genotyped by PCR analysis of tail DNA, and the non-5 × FAD male littermates served as WT mice. Mice were housed 4 animals per cage under a 12/12 h light/dark cycle with ad libitum access to food and water. The behavioral experiments were performed in the Center for Performing Behavioral Studies, Hadassah Hebrew University Medical Center, and the mice were sacrificed there. The experimental protocols were approved by the Institutional Ethics Committee of The Hebrew University of Jerusalem.

VBIT-4 and VBIT-12 were dissolved in DMSO (80 mg/ml) and then diluted in drinking water to a final concentration of 0.0625 mg/ml; assuming a 25-g mouse drinks about 8 ml water daily, it consumed 20 mg/kg of the compound (*n* = 9). Control untreated 5 × FAD mice (*n* = 8) received water containing DMSO (0.36%). To test the effect of VBIT-4 in WT mice, a group of WT mice was also given VBIT-4 in the drinking water (*n* = 10). The drinking solutions were given twice a week, with 1 day of water-only in between. Following 5 months of treatment (at age 7.5–8 months), cognitive performance of the mice was tested with T-maze, Y-maze, Open field habituation, and radial arm water maze tests.

At the end of the experiments, the mice were anesthetized with a lethal dose of pentobarbital and perfused via the ascending aorta with ice-cold phosphate-buffered saline (PBS), followed by cold 4% paraformaldehyde. Fixed brains were removed, further incubated for 24 h with 4% paraformaldehyde in PBS, then kept at PBS and transferred to us from Hadassah Hebrew University Medical Center. Brains were mounted and sectioned for immunohistochemistry (IHC), IF, thioflavin, or TUNEL staining.

### Behavioral tests

The following cognitive tests were performed, as described previously [46].

*Radial arm water maze* evaluates working memory, spatial learning, and cognitive ability [47]. The apparatus consists of 6 arms, 30 cm in length, converging on a central 40 × 40 cm^2^ pool filled with water to cover a plexiglass platform, located at the end of one arm. During each trial, the mouse was released from the end of a pseudo-randomly selected arm (counterbalanced across trials) and was required to navigate to a submerged platform at the end of a goal arm in 60 s. The platform location remained consistent throughout the experiment for each animal. Mice were run for 30 trials in total (15 on each day), every three trials were averaged and determined as a “block” (for example, block 1 was the average of trials 1–3 on day 1). On day 1, the animal was trained to locate the platform (12 trials alternating between hidden and visible platforms and the last three trials with a hidden platform). On day 2, the platform was hidden in all 15 trials. The last three trials of each day, i.e., block 5 (t5) and block 10 (t10), were considered “test trials”. Block 6 (t6), which can be considered as long-term memory, was calculated. The time to reach the platform and the number of errors (entry into the wrong arm) for each animal were recorded automatically using EthoVision XT (Noldus, Leesburg, VA).

*Open-field habituation* evaluates long-term non-associative, non-aversive spatial learning [48] by measuring the decrease in the exploratory activity of an animal in a test session carried out 24 h after the first exploration session (delta of 2^nd^ session − 1^st^ session). Animals were placed in a 40 cm × 50 cm × 60 cm open field box for a 5-min period. Twenty-four hours later, animals were re-exposed to the same environment for a 5-min period. The time in a mobile state and the distance traveled were measured in both sessions using EthoVision XT (Noldus, Leesburg, VA). A larger delta between days (shorter time in a mobile state during the test session compared to the first session) represented intact learning.

*Y-maze* evaluates short-term memory. The Y-maze is a 3-arm maze with all arms at equal angles. Each arm was 30 cm in length and 5 cm in width, with walls of 12-cm high. Mice were initially placed in the middle, and the sequence of arm entries was recorded manually for each mouse over an 8-min period. The triads with all three arms represented (i.e., ABC, CAB, or BCA, but not ABB) were considered “correct triads” [49].

*T-maze* was used to assess spatial long-term memory and alternation behavior, that is, to determine the mouse's ability to recognize and differentiate between a new unknown and a familiar compartment. The T-shaped maze was made of plastic with two arms 45 cm in length that extended at a right angle from a 57-cm-long alley. The arms had a width of 10 cm and were surrounded by 10-cm-high walls. The test consists of two trials with an interval of 24 h. During the interval, animals were put back in their home cages. During an 8-min acquisition trial, one of the short arms was closed. In a 3-min retention trial, mice had access to both arms and to the alley. The numbers of entries into the unfamiliar arm and the time spent in the unfamiliar arm were recorded. Mice normally tend to enter more times and spend more time in the new unknown arm than in the familiar one or in the alley [50].

#### Histological, immunohistochemistry, and immunofluorescence analyses of the brain

IHC and IF staining were performed on 5-μm-thick brain sections, with antigen retrieval performed by 30-min incubation in 0.01 M citrate buffer, pH 6.0 at 95–98 °C. Sections were washed with PBS (pH 7.4) containing 0.1% Triton-X100 (PBST). Non-specific antibody binding was reduced by incubating the sections in 10% normal goat serum for 2 h, followed by overnight incubation at 4 °C with primary antibodies (see Additional file 1: Table S2). For immunohistochemical staining, endogenous peroxidase activity was blocked by incubating the sections in 3% H_2_O_2_ for 15 min. After washing with PBST, the sections were incubated for 2 h with the appropriate HRP-conjugated secondary antibodies. Sections were washed in PBST, and peroxidase activity was visualized by incubating the section with 3,3-diaminobenzidine (DAB) (ImmPact-DAB, Burlingame, CA). After rinsing in water, the sections were counterstained with hematoxylin, and mounted with EUKITT mounting medium (Orsatech, London, UK). Finally, the sections were imaged under a panoramic scanner (3DHISTECH Ltd, Hungary) with the same light intensity and exposure time. Quantification of the immunostained images was carried out using HistoQuant software (Quant Center 2.0 software, 3DHISTECH Ltd, Hungary).

For IF staining, following incubation with the primary antibody, the fluorophore-conjugated secondary antibodies listed in Additional file 1: Table S2 were used. The cell nuclei were stained with DAPI (0.07 μg/ml). Sections were mounted with fluoroshield mounting medium (Immunobioscience, Mukilteo, WA), and viewed with an Olympus IX81 confocal microscope. Quantification of staining intensities was performed using Image J, with five randomly selected images examined in each section. Sections from at least three mouse brains for each group were subjected to immunostaining. Confocal microscope setting during image collection was constant for the same experiment and the sections were analyzed twice by two of the authors with no identity of the samples.

Protein levels were quantified based on the staining intensity in images selected over the whole area of the section using Image J software. Data were exported to Excel for statistical analysis. The background staining intensity for each image was subtracted from the total staining intensity.

### Statistics

Behavioral data were analyzed using one-way ANOVA, followed by Tukey’s post-hoc analysis. An average value was determined for each group, and data are expressed as mean ± SD. Other results are presented as the means ± SEM of results obtained from three or more independent experiments. A difference was considered statistically significant when the *P*-value was deemed < 0.05 (*), < 0.01 (**), < 0.001 (***), or < 0.0001 (****), assessed through unpaired Student’s two-tailed *t*-test.

## Results

### Aβ triggers VDAC1 and p53 overexpression in primary neuronal cultures, leading to apoptosis, which was prevented by VBIT-4

Previously, we have shown that VDAC1 overexpression leads to its oligomerization followed by apoptosis [34, 41, 51], and both are inhibited by the VDAC1-interacting molecule, VBIT-4 (Additional file 1: Fig. S1a). Consistently, in this study, cisplatin applied to the cell line SH-SY5Y of neuronal origin induced VDAC1 overexpression, VDAC1 oligomerization, and cell death, and reduced cell viability (Additional file 1: Fig. S1b–f), which were all inhibited by VBIT-4.

To determine whether Aβ-induced cell death [28, 29, 31] is also associated with VDAC1 overexpression, we used rat hippocampal primary neuronal culture and expressed the human *APP* gene carrying the Swedish and London mutations (APP_swe/lnd_) [52] together with GFP using a viral expression system (Additional file 1: Fig. S1g). In this system, the overproduced Aβ was secreted to the medium (conditioned medium) [53]. An ELISA-based assay indicated that this conditioned medium contained Aβ at 24 ± 1.2 pg/ml (*n* = 3 cultures).

**Fig. 1:**
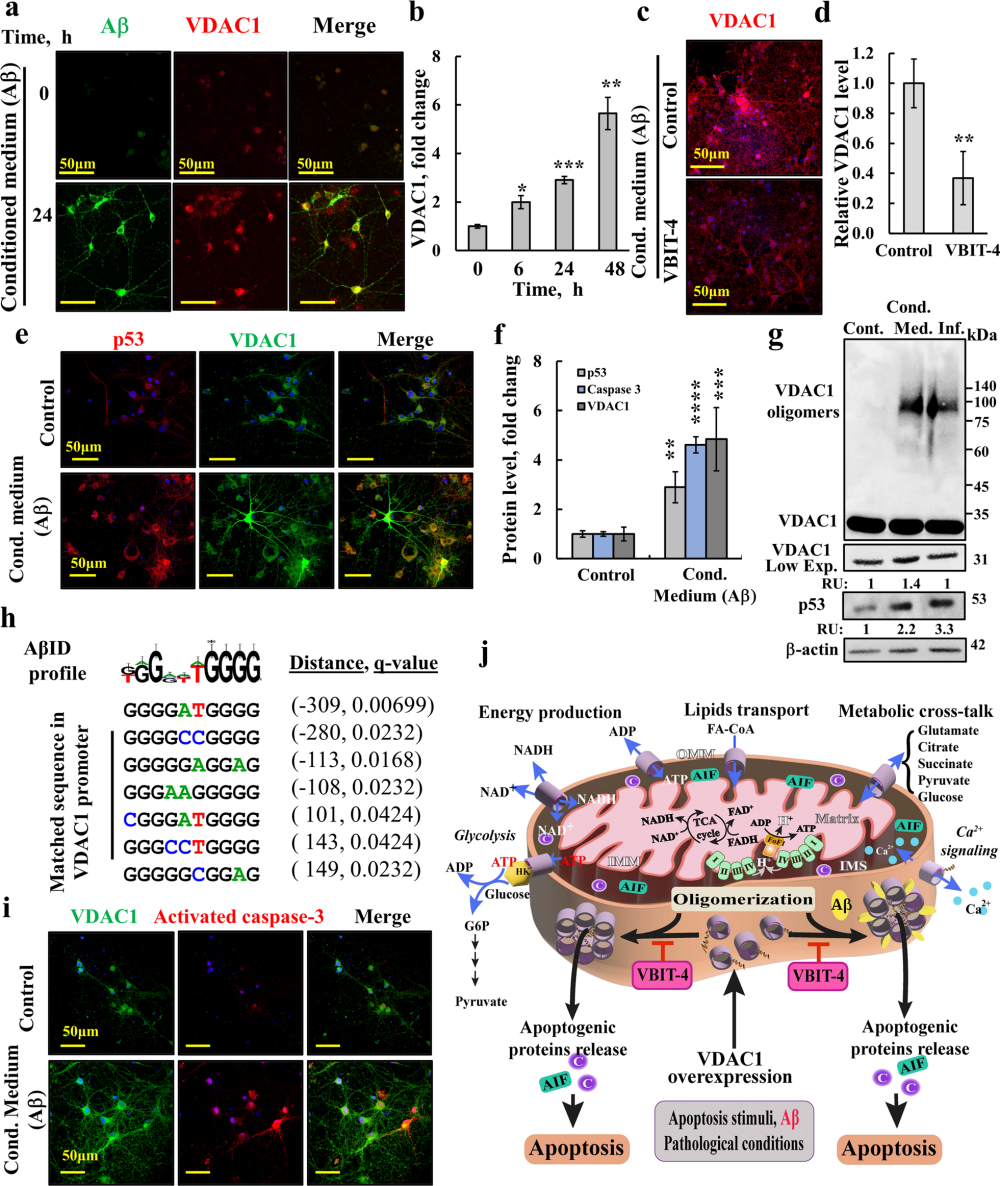
Aβ induces VDAC1 overexpression, oligomerization, and apoptotic cell death in primary neural cultures. Primary neural cultures were infected with App swe/lnd-EGFP Sindbis virus for 16 h (Additional file 1: Fig. S1g) to overexpress and secrete Aβ into the medium (conditioned medium, Cond. Med.). **a** Cells were incubated with and without 50% conditioned medium for 6, 24, and 48 h and IF stained for VDAC1. **b** Quantification of VDAC1 IF staining. **c**, **d** IF staining for VDAC1 of cells incubated for 48 h with 50% conditioned medium in the presence and absence of VBIT-4 (10 μM), and its quantification. **e**–**f** Cultured neurons incubated with and without 50% conditioned medium for 24 h were co-immunostained for VDAC1 and p53 (**e**) and their expression levels were quantified (**f**). **g** Primary neural cultures were infected with App swe/lnd-EGFP Sindbis virus for 20 h (Inf.), then conditioned medium (Cond. Med.) was collected, and control neuronal culture was incubated with and without 50% conditioned medium for 48 h and subjected to immunoblotting for VDAC1 and p53. The p53 and monomeric VDAC1 levels are shown in the bottom in relative units (RU). The low exposure (Low Exp.) is presented to show the increase in monomeric VDAC1 levels. The positions of VDAC1 monomers and oligomers and of the molecular weight standards are indicated. **h** VDAC1 promoter sites that match the sequence profiles generated from Aβ ID decamers [54]. The distance from the VDAC1 transcription start site and the q-value of a motif occurrence are presented. **i** Immunostaining for activated caspase-3 and VDAC1, in control and conditioned medium treated culture. Quantification of activated caspase-3 levels are shown in (**f**). **j** Proposed coupling of VDAC1 overexpression induced by apoptosis stimuli or Aβ and VDAC1 oligomerization forming a large channel, with and without Aβ participation, mediating the release of apoptogenic protein cytochrome *c* (Cyto *c*) and apoptosis-inducing factor (AIF) from the intermembrane space (IMS). The VDAC1-interacting molecule, VBIT-4, prevents VDAC1 oligomerization and apoptosis. The functions of VDAC1 in cell life include (blue arrows): control of metabolic cross-talk between the mitochondria and the rest of the cell; transport of Ca^2+^ to and from the IMS; mediation of cellular energy production by transporting ATP/ADP and NAD ^+^/NADH and fatty acid transport as acyl-CoA (FA-CoA) form, and regulation of glycolysis via binding of hexokinase (HK). The TCA cycle, the electron transport chain (ETC), and the ATP synthase (F0F1) are also presented

Neuronal cultures incubated with the conditioned medium (diluted 1:1 with the culture medium) [53] showed a time-dependent increase in VDAC1 level (Fig. 1a,b), which was reduced by VBIT-4 (Fig. 1c,d). Neurons with increased VDAC1 levels also showed increased expression of p53 (Fig. 1e,f). Similar results were obtained by immunoblotting analysis (Fig. 1g). Interestingly, both neurons that were infected to express APP and those that were exposed to the conditioned medium showed high levels of trimeric VDAC1, suggesting that Aβ leads to VDAC1 oligomerization (Fig. 1g) [28].

Aβ is proposed to act as a putative transcription factor by binding to the Aβ–interacting domain (AβID) in the DNA sequence (G/T)GG(A/G)(G/T)TGGGG, which is found in *APP*, *BACE1*, and *APOE* promoters [54, 55]. Additionally, soluble Aβ translocates to the nucleus where it regulates gene transcription [54, 55], including activation of the p53 promoter [56]. Here, we identified the AβID consensus sequence at seven sites in the* VDAC1* promoter region, with the most significant site being GGGGATGGGG (Fig. 1h, Additional file 1: Table S3). These results suggest that Aβ can enhance VDAC1 expression directly by binding to the *VDAC1* promoter or indirectly by activating the p53 promoter.

To determine whether Aβ-induced cell death [28, 29, 31] is associated with VDAC1 overexpression, we analyzed the expression of activated caspase-3 and found that it was increased in cells with increased VDAC1 expression (Fig. 1i,f).

These results together with previous findings [22, 41] suggest that Aβ [18] and certain pathological conditions [37] lead to VDAC1 overexpression and oligomerization, forming a channel large enough for pro-apoptotic proteins and mtDNA to cross the outer mitochondrial membrane (OMM) and to subsequently induce apoptosis and inflammation (Fig. 1j, Additional file 1: Fig. S1h). VBIT-4, by preventing VDAC1 oligomerization, protects against mitochondria dysfunction, apoptosis and inflammation (Fig. 1j, Additional file 1: Fig. S1h).

VBIT-4, by protecting against mitochondria dysfunction, allows VDAC1 to function to control the metabolic cross-talk between the mitochondria and the rest of the cell, and transport of Ca^2+^ and fatty acid as acyl-CoA mediates cellular energy production by transporting ATP/ADP and NAD +/NADH, and regulates glycolysis via binding of hexokinase (HK) (Fig. 1j). All are important for cell life.

### VBIT-4 has a stable metabolic profile, and it crosses the blood–brain barrier

**Fig. 2:**
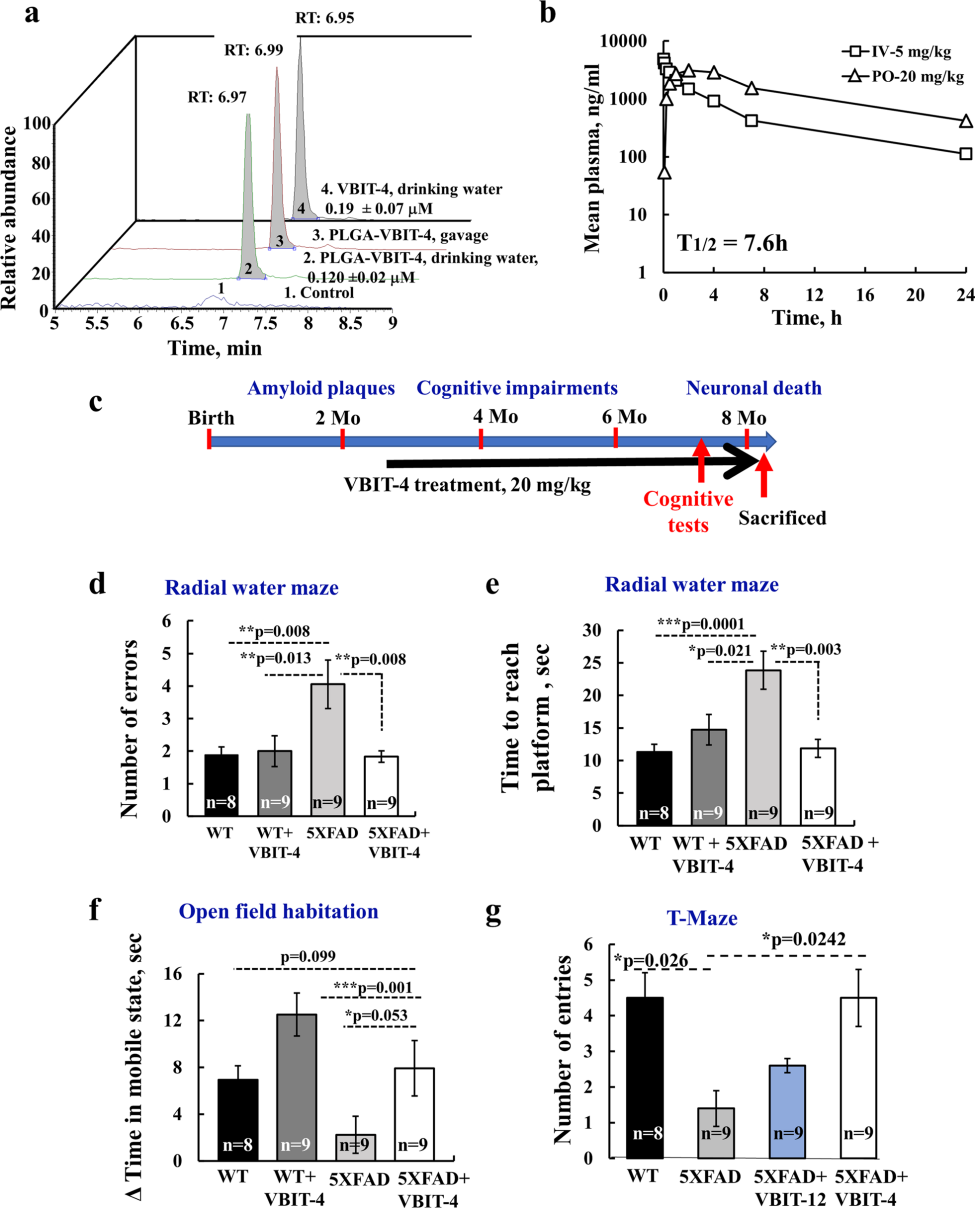
VBIT-4 improves the cognitive performance of 5 × FAD mice. **a** Representative LC–MS/MS analysis of VBIT-4 concentration in VBIT-4-treated mouse brain extracts [1]. Control (PBS no peak detected); (2) PLGA nano-particle-VBIT-4 administered in drinking water; (3) PLGA-VBIT-4 administered through gavage; (4) VBIT-4 in drinking water. The retention time (RT) and VBIT-4 concentration in the brain is indicated. **b** PK profile studied in rats following administration of VBIT-4 by IV (5 mg/kg) and PO (20 mg/kg). The observed PK parameters showed moderate-high oral bioavailability. (F 65%), *T*_1/2_ = 7.6 h, *C*max = 3310 ng/ml, *T*max = 1.33 h, AUCinf = 38,369 h*ng/ml. **c** Disease progression timeline in 5 × FAD mice and the experimental protocol for VBIT-4-treatment. Mice behavioral tests were performed at the age of 7–7.5 months, about 5 months after initiating VBIT-4 or control (0.36% DMSO) treatment (number of mice in each group is indicated). Effect of VBIT-4 on WT was also tested (*n* = 8). **d**, **e** Performance at RAWM was analyzed at trial 6 at the end of day 1, and was expressed as the number of errors (**d**) or time it took to reach the platform (**e**). For (**d**), a one-way ANOVA yielded a significant difference among the groups [*f*(3,28) = 5.4, *P* = 0.005], and Tukey post-hoc analysis revealed that 5 × FAD mice performed more poorly than WT (*P* = 0.008) and WT-VBIT-4 mice (*P* = 0.013). 5 × FAD-VBIT-4-treated mice performed much better than the 5 × FAD mice (*P* = 0.008). For (**e**), a one-way ANOVA yielded a significant difference among the groups [*f*(3,30) = 6.9, *P* = 0.001]. A Tukey post-hoc analysis revealed that 5 × FAD mice performed more poorly than the WT mice (*P* = 0.001), and the WT + VBIT-4 (*P* = 0.021); 5 × FAD-VBIT-4 mice performed better than 5 × FAD mice (*P* = 0.003). **f** An open field habituation test yielded a significant difference among the groups in the time they spent in a mobile state [*f*(3,30) = 4.5, *P* = 0.009]. 5 × FAD mice spent less time than WT mice, and 5 × FAD-VBIT-4 treated mice spent longer than 5 × FAD mice (*P* = 0.053), and similar to that of WT mice. WT-VBIT-4-treated mice (*P* = 0.001) spent a longer time than the WT. A one-tailed t-test revealed significantly better performance of WT than 5 × FAD mice (*P* = 0.013). **g** Number of entries in a T-maze. A one-way ANOVA yielded a significant difference among the groups [*f*(3,28) = 4.22, *P* = 0.014], Tukey post-hoc analysis revealed that 5 × FAD mice performed more poorly than WT mice (*P* = 0.026), and that the 5 × FAD-VBIT-4-treated mice performed better than the 5 × FAD mice (*P* = 0.0242). 5 × FAD-VBIT-12-treated mice performed better than 5 × FAD but less than the VBIT-4-treated mice

To determine if VBIT-4 can cross the blood–brain barrier and mitigate brain pathology when administered in drinking water (either encapsulated in poly lactic-co-glycolic acid (PLGA)-nano-particles, naked), or by gavage, its concentration in the brain was analyzed using liquid chromatography mass spectroscopy (LC/MS/MS) (Fig. 2a). In a preliminary study, VBIT-4 showed an elimination half-life (PK) of 7.6 h (Fig. 2b) indicating a stable metabolic profile, and high plasma protein binding with the bound compound fraction possibly serving as a reservoir from which a slow release can occur (Additional file 1: Table S4). A single-dose toxicity study for VBIT-4 in rats showed no treatment-related mortality or clinical signs, and no significant changes in hematology or in serum chemistry parameters (Additional file 1: Table S4). Overall, the pharmacokinetics data and *in-vivo* efficacy of VBIT-4 appear useful in predicting an effective therapeutic dose.

### VBIT-4 prevents cognitive deterioration in 5 × FAD mice

In previous studies, we demonstrated that VBIT-4 prevents apoptosis-associated processes such as cytosolic Ca^2+^ elevation and ROS production [41], leading to amelioration of the disease-associated processes in several disease models [26, 42, 43]. Therefore, we tested the effects of VBIT-4 on the AD-like pathology in a 5** × **FAD transgenic mouse model carrying five familial AD (FAD) mutations [45]. This model exhibits Aβ plaques at 2 months, synaptic degeneration and neuronal loss beginning at about 4 months, and massive neuronal loss at 8–9 months of age [45]. The experimental timeline of VBIT-4 treatment and its molecular structure, are presented in Fig. 2c. and Additional file 1: Fig. S1a.

To evaluate the effects of VBIT-4 on several aspects of cognitive performance, 5** × **FAD mice were given VBIT-4 in drinking water twice a week (20 mg/kg), and at the age of 7–7.5 months, they were subjected to four behavioral tests (radial arm water maze, Y-maze, T-maze, and open field tests) (Fig. 2d–g). Results of the radial arm water maze showed that the 5** × **FAD mice made about twice the errors of wild-type (WT) mice and took twice the time to reach the platform (Fig. 2d, e). In contrast, the performance of VBIT-4-treated 5** × **FAD mice was similar to that of the WT in both error numbers and time to reach the platform (Fig. 2d, e).

Next, we used the open field habituation test to evaluate long-term non-associative, non-aversive spatial learning [48]. The 5** × **FAD mice spent about 25% of the time that the WT spent in a mobile state. When treated with VBIT-4, they performed similarly to the WT mice (Fig. 2f). Interestingly, in this test, VBIT-4 also improved the performance of the WT group, perhaps affecting the exploratory activity and reactivity of the mice to a novel environment.

The T-maze test assesses spatial long-term memory and alternation behavior, including the mouse's ability to recognize and differentiate between a new and a familiar compartment. Here, 5** × **FAD mice made significantly fewer entries to the correct arm than the WT mice. The VBIT-4-treated mice had similar entries as the WT group (Fig. 2g). In this test, we also examined another VDAC1-interacting molecule, VBIT-12 [41], and found it to be less effective than VBIT-4 (Fig. 2g, blue bar).

Finally, the Y-maze test revealed that the 5** × **FAD mice had fewer correct triads than the WT group, but when treated with VBIT-4, their performance was similar to that of the WT group (Additional file 1: Fig. S2a). Thus, oral administration of VBIT-4 rescued several aspects of cognitive function in the AD-like 5** × **FAD mice.

It should be noted that treating WT with VBIT-4 had no effect on the expression of specific markers for astrocytes, microglia, or neurons (Additional file 1: Fig. S2b–e).

The finding that VBIT-4 had no effect in WT healthy mice could be due to the fact that the VDAC1 levels are too low to lead a shift of VDAC1 from monomeric to oligomeric form. Therefore, in WT mice no apoptosis occurs, and VDAC1 mediates the normal metabolic processes. This is supported by a cell-based study showing that VBIT-4 does not interfere with normal mitochondria function [41].

### VDAC1 is overexpressed in the neuropil surrounding Aβ plaques in the 5 × FAD mouse brain, and VBIT-4 protects against neuronal loss

**Fig. 3:**
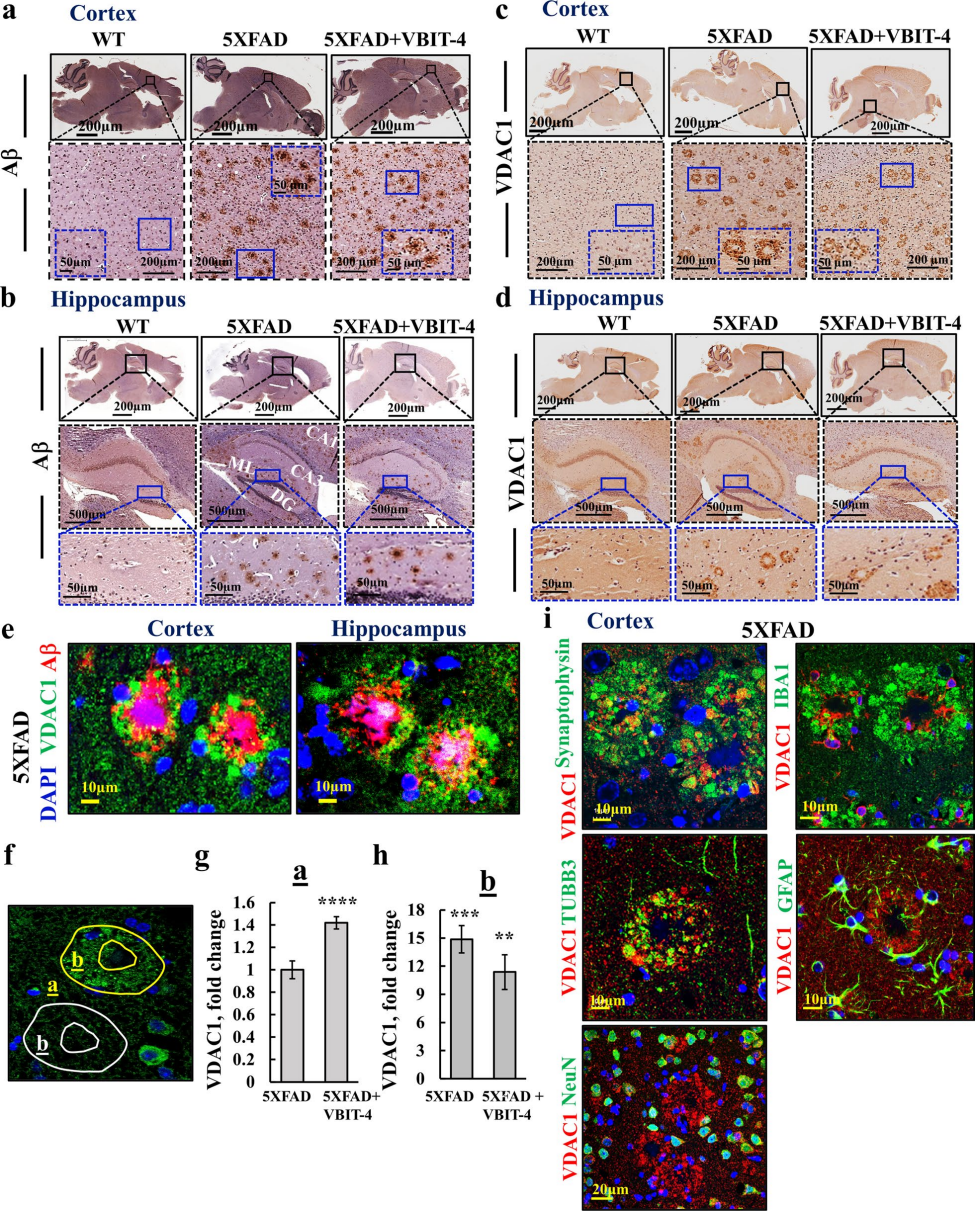
VDAC1 is highly expressed in the neuropil surrounding the Aβ plaques of the 5 × FAD mouse model. **a**–**d** Representative cortical and hippocampal sections from WT and 5 × FAD mice treated and untreated with VBIT-4, IHC stained for Aβ (**a**, **b**) or VDAC1 (**c**, **d**). Higher magnifications of selected areas are shown within the dashed-line squares. **e** Confocal IF images of cortical and hippocampal sections from 5 × FAD mice co-IF-stained for Aβ and VDAC1. The over-expressed VDAC1 rings are formed around the Aβ plaques. **f**–**h** Quantitative analysis of VDAC1 expression levels in cortical sections outside the plaques (**g**), (area **a** in **f**) and in the neuropil surrounding the Aβ plaques (**h**), (area b in **f**); in **h**, numbers are relative to levels outside of the plaque (**a**). Results show means ± SEM (*n* = 3). **P < 0.01, ***P < 0.001, ****P < 0.0001. **i** Representative Co-IF staining of cortical sections from 5 × FAD mice for VDAC1 and neuronal markers (TUBB3, NeuN, synaptophysin); microglia (IBA1) or astrocytes (GFAP)

Next, mice that underwent behavioral testing were sacrificed, and their brains were used to examine the pathological features of the disease. As pathologies in the hippocampus and cortex are closely associated with AD development [57], we focused on these brain regions. IHC staining of brain sections of 5x FAD mice showed that Aβ was distributed throughout the cortex and hippocampus, with formation of numerous plaques (Fig. 3a, b). Immunostaining showed that VDAC1 in the WT mice was evenly distributed, while the 5 × FAD mice showed punctate staining throughout the sections and strong staining in ring-like structures (Fig. 3c, d). Similar staining patterns for Aβ and VDAC1 were obtained in VBIT-4-treated 5 x FAD mice.

To determine the relationship between Aβ plaques and VDAC1 rings, we carried out co-immunofluorescence (co-IF) staining for Aβ and VDAC1. Results demonstrated that the strongly stained ring-like structures represented overexpressed VDAC1 in the neuropils (composed of mostly unmyelinated axons, dendrites, and glial cell processes that form a synaptically dense region) surrounding the Aβ plaques (Fig. 3e, Additional file 1: Fig. S3a). The intensity of VDAC1 in regions outside the rings (area a in Fig. 3f) was significantly higher in the VBIT-4-treated 5 × FAD mice (1.4-fold) than in the untreated 5 × FAD mice (Fig. 3g). In VDAC1-expressing rings (area b in Fig. 3f), the VDAC1 level was dramatically elevated (by 15-fold) relative to area a, and in the VBIT-4 treated mice, this elevation was significantly smaller (12-fold) (Fig. 3h).

To identify the cell compartments surrounding the Aβ plaques that overexpressed VDAC1, we used four neuron-specific markers: synaptophysin to identify presynaptic terminals; class III beta-tubulin (TUBB3) that stains neuronal cell bodies, dendrites, and axons; neuronal nuclear protein (NeuN) to stain the neuronal somas; and post-synaptic density protein-95 (PSD-95). We also stained for the glial fibrillar acidic protein (GFAP) to identify astrocytes, and for the Ca^2+^-binding adaptor molecule-1 (IBA-1) to identify microglia (Fig. 3i). The results showed co-localization of VDAC1 in the rings only with synaptophysin and TUBB3, but not with the astrocytic or microglial markers. The result suggested that the overexpressed VDAC1 was in neuronal terminals surrounding Aβ plaques.

**Fig. 4:**
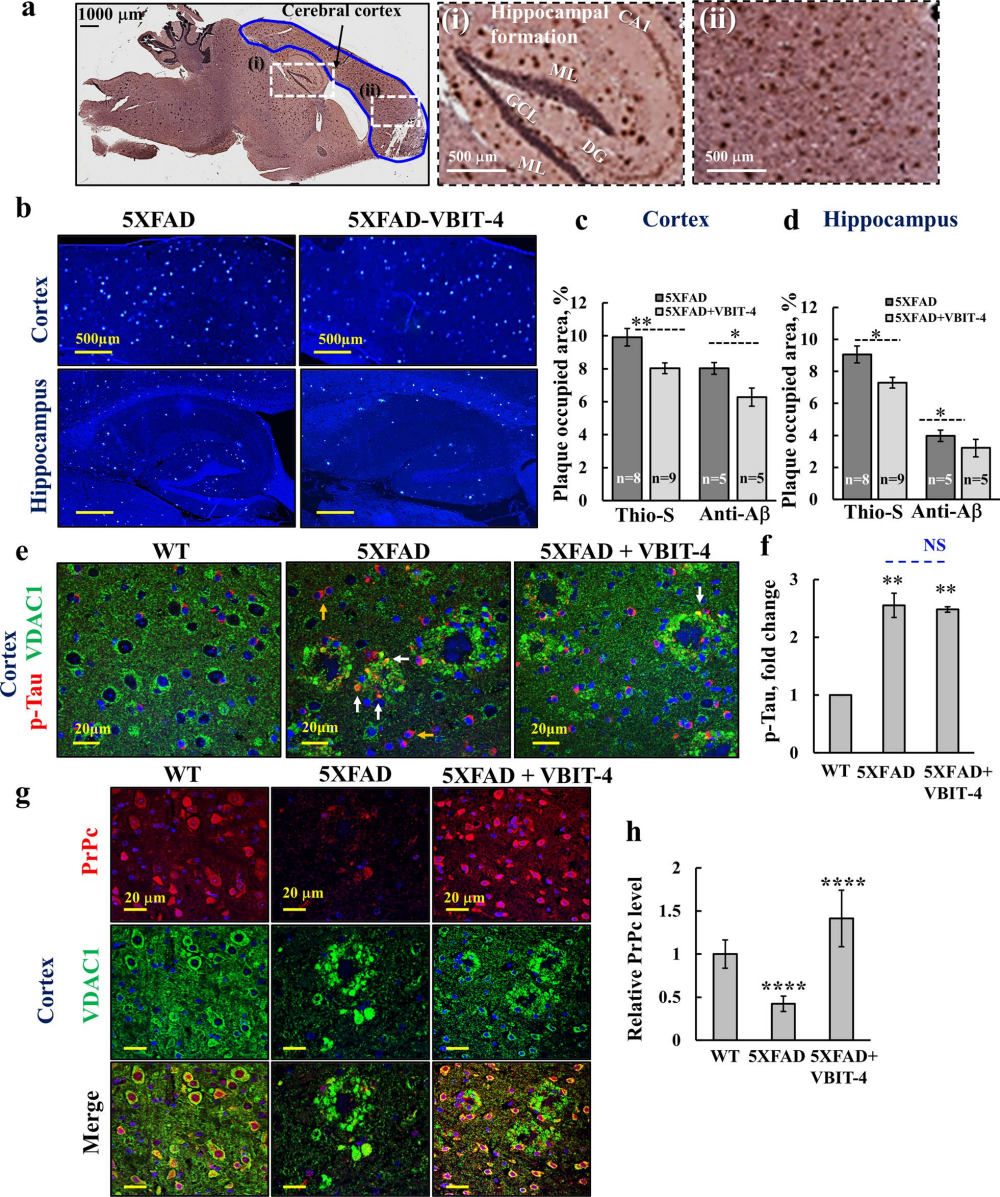
Effect of VBIT-4 treatment on the levels of Aβ plaques, and the expression of p-Tau and PrPc in the 5 × FAD brain. **a** Brain sections from 5 × FAD mice were immunostained for Aβ using anti-Aβ antibodies. The cerebral cortex and hippocampal formation that were analyzed in this study are enlarged in (i) and (ii) panels. The CA1 (cornu ammonis subfield 1), ML (molecular layers), GCL (granule cell layer), and DG (dentate gyrus) are indicated. **b** Representative thioflavin-S (Thio-s) staining of Aβ plaques in cortical and hippocampal sections from VBIT-4-treated and untreated 5 × FAD mice. **c**, **d** Areas occupied by Aβ plaques in the cortex (**c**) and hippocampus (**d**), as analyzed from Thio-s or anti-Aβ antibodies (anti-Aβ), are expressed as mean ± SE (*n* = 5–9 as indicated). **e**–**h** IF staining and quantification of p-Tau and VDAC1 (**e**, **f**), and of VDAC1 and PrPc (**g**, **h**) in cortical sections from WT and VBIT-4-treated and untreated 5 × FAD mice. and their quantification. Results show means ± SEM (*n* = 3 animals for each group, with  IF was performed 2–3 times for each group), **P < 0.01, ***P < 0.001, ****P < 0.0001. NS, not significant. *p-*value in blue color represents the significance of VBIT-4-treated 5 × FAD mice relative to untreated mice

Given the effect of VBIT-4 in improving learning and memory in the AD mice, we next analyzed the effect of VBIT-4 on the levels of Aβ plaques and proteins implicated in AD pathology. The hippocampus and cortical areas occupied by Aβ plaques were analyzed following thioflavin-S staining and immunostaining for Aβ (Fig. 4a–d). For both hippocampal and cortical Aβ staining, there was about a 20% decrease in the area occupied by Aβ plaques in VBIT-4-treated relative to untreated 5 × FAD mice.

We then examined the effects of VBIT-4 on other proteins implicated in AD pathology and found that the level of p-Tau was increased (2.7-fold) in 5 × FAD, but VBIT-4 treatment had no effect on its level (Fig. 4e, f).

The cellular prion protein (PrPc) [58] with proposed neuroprotective effects [59] has been found to be lower in AD than in non-AD individuals [60]. Here, we found about 2.5-fold lower expression of PrPc in 5 × FAD mouse brains than in WT brains, while VBIT-4 treatment increased the PrPc level in 5 × FAD mice to be even higher than that in WT mice (Fig. 4g,h).

Finally, we analyzed the level of islet amyloid polypeptide amylin. Amylin is a peptide hormone synthesized and co-secreted with insulin by pancreatic β cells [61], and mediates toxic effect via mitochondrial dysfunction [62]. The results showed that the amylin expression levels were higher in both the cortex and hippocampus of 5 × FAD mice compared to WT mice, but VBIT-4 treatment  had no significant effect on its expression levels (Additional file 1: Fig. S3b–e).

Thus, among the four tested proteins whose expression levels are greatly modified in the context of AD, PrPc and Aβ expression levels were altered by VBIT-4, while p-Tau and amylin were not. The decrease in Aβ level by VBIT-4 may result from the prevention of plaque formation/growth or its increased removal.

### VBIT-4 protects against AD-related neuronal loss

**Fig. 5:**
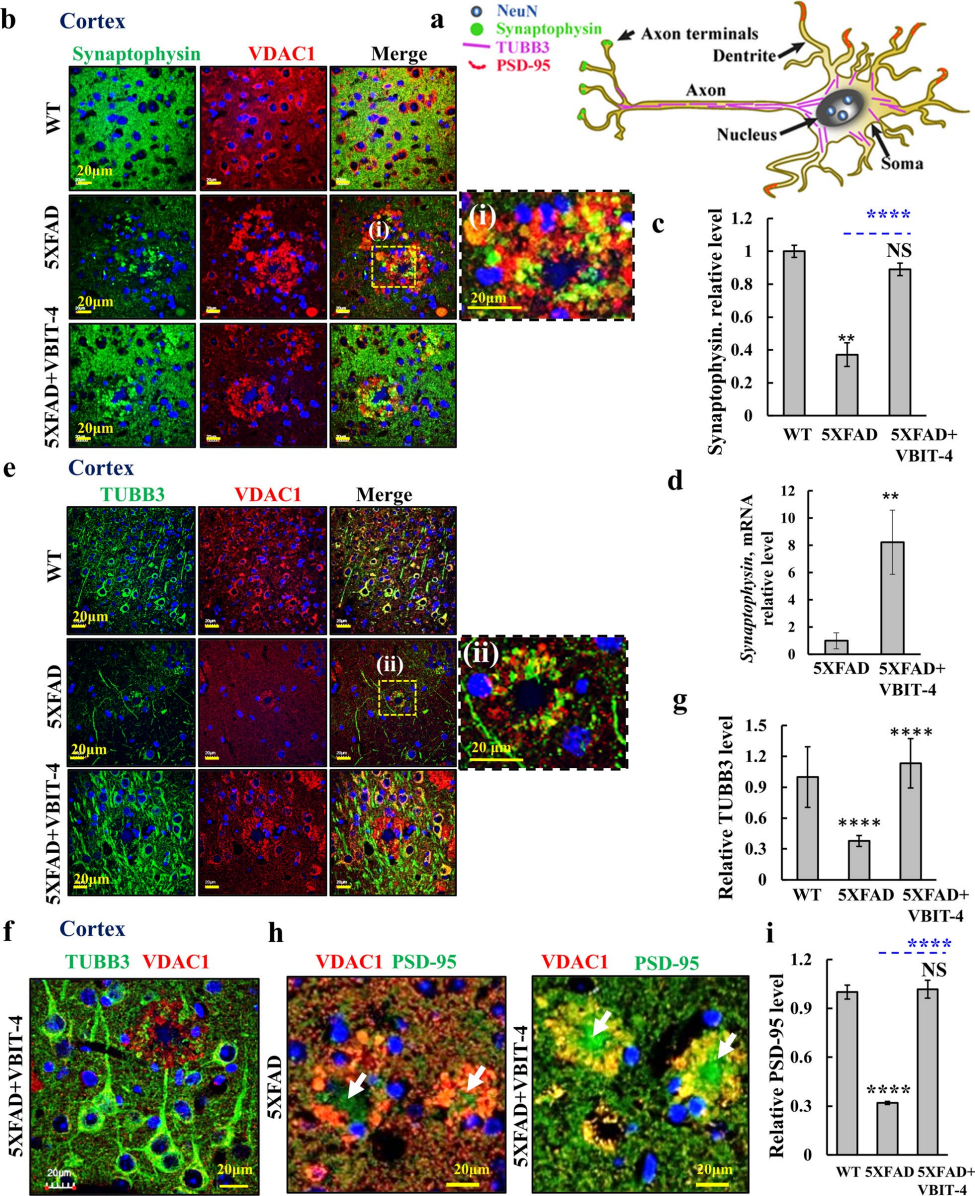
Overexpressed VDAC1 around the Aβ-plaques is localized to neurons. **a** Schematic presentation of four neuronal markers localized to different compartments within a neuron. **b**–**g** Co-immunostaining for VDAC1 and synaptophysin (**b**), TUBB3, (**e**) of cortical sections from WT, untreated- and VBIT-4-treated 5 × FAD mice. (i) and (ii) are enlargements to show co-localization of synaptophysin or TUBB3 with VDAC1. Quantitative analysis of synaptophysin IF staining (**c**) and its mRNA levels (**d**) and of TUBB3 (**g**). **f** Cortical sections from VBIT-4-treated 5 × FAD mouse IF with anti-TUBB3 and VDAC1 showing neurons with their terminals reaching the Aβ plaque. Nuclei were stained with DAPI. **h**, **i** Cortical sections from untreated and VBIT-4 treated 5 × FAD mice immunostained for PSD-95 and VDAC1 (**h**) and PSD-95 quantification analysis (**i**). Arrows point to dendrites with no overexpressed VDAC1. Results show means ± SEM (*n* = 3–4 mice), **P < 0.01, ****P < 0.0001. *P-*value in blue color represents the significance of VBIT-4-treated relative to untreated 5 × FAD mice. NS, not significant

To determine the effect of VBIT-4 on neuronal survival in 5 × FAD mice and to further identify the cell compartments surrounding the Aβ plaques that overexpress VDAC1, we used four neuron-specific markers: synaptophysin, TUBB3, NeuN, and PSD-95 (Fig. 5a). IF staining for synaptophysin in both cortex and hippocampus showed threefold decrease in the 5 × FAD mice relative to the WT mice, while the VBIT-4-treated 5 × FAD mice did not exhibit this decrease (Fig. 5b,c). The prevention of synaptophysin decrease by VBIT-4 treatment was confirmed by q-RT-PCR (Fig. 5d).

TUBB3 was highly expressed in neurons in WT mice, but was decreased by 2.5 folds in the 5 × FAD mice, and the decrease was prevented by VBIT-4 treatment (Fig. 5e–g), suggesting that VBIT-4 prevented the neuronal loss in the 5 × FAD mice.

Next, staining for the neuronal soma protein NeuN, an RNA-binding protein specific for post-mitotic neurons predominantly associated with cell nuclei [63], was lacking in the VDAC1-overexpressing neuropils surrounding Aβ plaques (Additional file 1: Fig. S4a). In addition, in the 5 × FAD mice, the structures that were stained with either DAPI or NeuN appeared smaller than those in the WT, which might represent apoptotic cells (Additional file 1: Fig. S4a). In VBIT-4-treated 5 × FAD mice, the nuclei and somas of neurons had similar sizes as those of the WT.

Next, we analyzed the effect of VBIT-4 on the expression level of PSD-95, a scaffolding protein involved in the assembly and function of the post-synaptic density complex. This protein is involved in anchoring receptors and ion channels, and plays an indispensable role in signal transmission and, hence, in cognition [64]. PSD-95 expression was decreased in 5 × FAD mice, but the decrease was prevented by VBIT-4 treatment (Fig. 5h, i, Additional file 1: Fig. S4d). Interestingly, most but not all PSD-95-expressing compartments in the Aβ plaques showed co-localization with the overexpressed VDAC1 (Fig. 5h, Additional file 1: Fig. S4d, white arrows). Similar results were obtained in the hippocampus (Additional file 1: Fig. S4b–d).

Taken together, in 5 × FAD mice, the co-localization of overexpressed VDAC1 with synaptophysin and TUBB3 [see Fig. 5b(i), e(ii)] suggests that the “ring” structures surrounding the Aβ plaques contain neuronal terminals overexpressing VDAC1, leading to cell death, and thereby neuronal loss. Treatment with VBIT-4 protected against synaptic and neuronal loss both in the cortex and in the hippocampus.

### VBIT-4 inhibits apoptosis in 5 × FAD mice

**Fig. 6:**
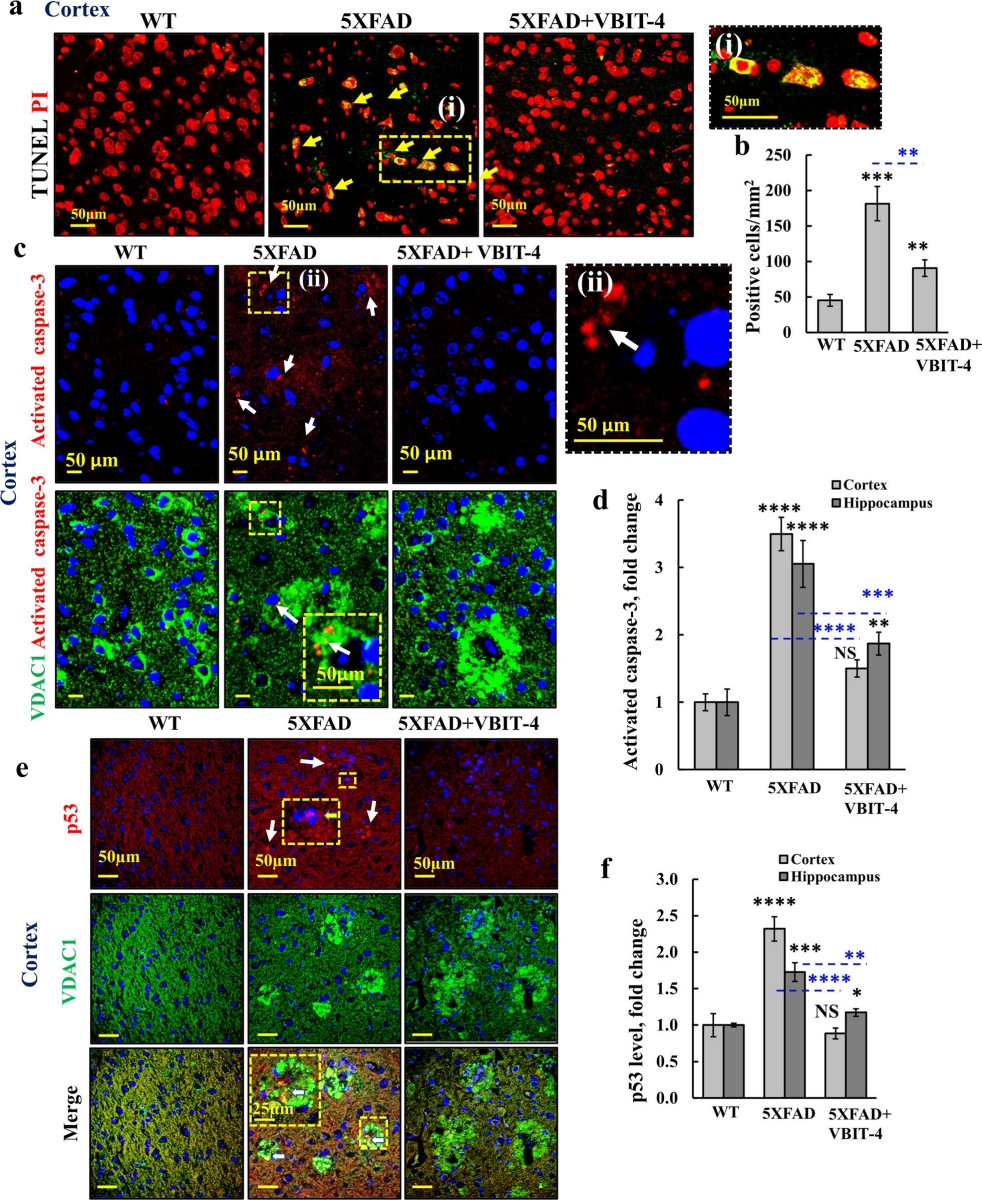
VBIT-4 treatment of 5 × FAD mice protects against cell death. **a** Representative TUNEL staining of cortical sections from WT, untreated-, and VBIT-4-treated-5 × FAD mice, with a magnification of the selected area (**i**). The arrows point to apoptotic cells stained green/yellow; red represents propidium iodine-stained nuclei. **b** Average number of TUNEL-stained cells per mm^2^. **c**–**f** Confocal images of cortical sections from WT, untreated- and VBIT-4-treated-5 × FAD mice co-immunostained for VDAC1 and activated caspase-3 (**c**) with a magnification of the selected area (ii), or p53 (**e**) and their quantifications (**d**, **f**). Results show means ± SEM (*n* = 3 mice), *P < 0.05, **P < 0.01, ***P < 0.001, ****P < 0.0001. *P-*value in blue color represents the significance of VBIT-4-treated relative to untreated 5 × FAD mice. NS, not significant

The overexpression of VDAC1 in the synaptic terminals surrounding Aβ plaques found in this study and previous report of association of VDAC1 overexpression with apoptotic cell death [34] suggest that VDAC1 may induce apoptotic neuronal death [34, 51]. Thus, apoptosis was evaluated by TUNEL staining and IF staining for activated caspase-3 expression, which was shown to be elevated in the brains of severe AD cases [65]. Relative to WT mice, the number of TUNEL-stained cells in the 5 × FAD mice was increased over 3 folds, while in the VBIT-4-treated 5 × FAD mice, the number was significantly reduced (Fig. 6a, b). Activated caspase-3 levels in the 5 × FAD mice were increased by 2.5 folds in both the cortex and the hippocampus, and the levels were greatly reduced in VBIT-4-treated 5 × FAD mice (Fig. 6c, d, Additional file 1: Fig. S5a).

Consistent with our results from the primary neuronal cultures, the expression level of p53, which regulates cell cycle, apoptosis, and senescence [66], was higher in the cortex and hippocampus of 5 × FAD mice than in WT mice, and that of VBIT-4-treated mice was similar to the WT group (Fig. 6e, f, Additional file 1: Fig. S5b). The p53 level also increased in some Aβ plaque-surrounding compartments, and was co-localized with the overexpressed VDAC1 (Fig. 6e, Additional file 1: Fig. S5b), suggesting mitochondrial localization [67].

### VBIT-4 prevents dysregulated metabolism in 5 × FAD mice

Neuro-metabolic dysfunctions leading to neurodegeneration, are associated with impaired glucose transport and metabolism, brain insulin resistance, and age-induced mitochondrial dysfunction [10, 36, 68, 69]. Considering impaired metabolism in AD [10, 36, 68] and VDAC1 regulation of metabolism [21, 24], we evaluated the expression of several metabolism-related proteins in 5 × FAD mice and the effects of VBIT-4 on their expression.

The glucose transporters (Gluts) are differentially expressed in the brain, with Glut-1 expressed in astrocytes, Glut-2 in microglia and neurons, and Glut-3 and insulin-regulated Glut-4 in neurons [70]. Glut-1 and Glut-3 are downregulated in AD [71, 72].

**Fig. 7:**
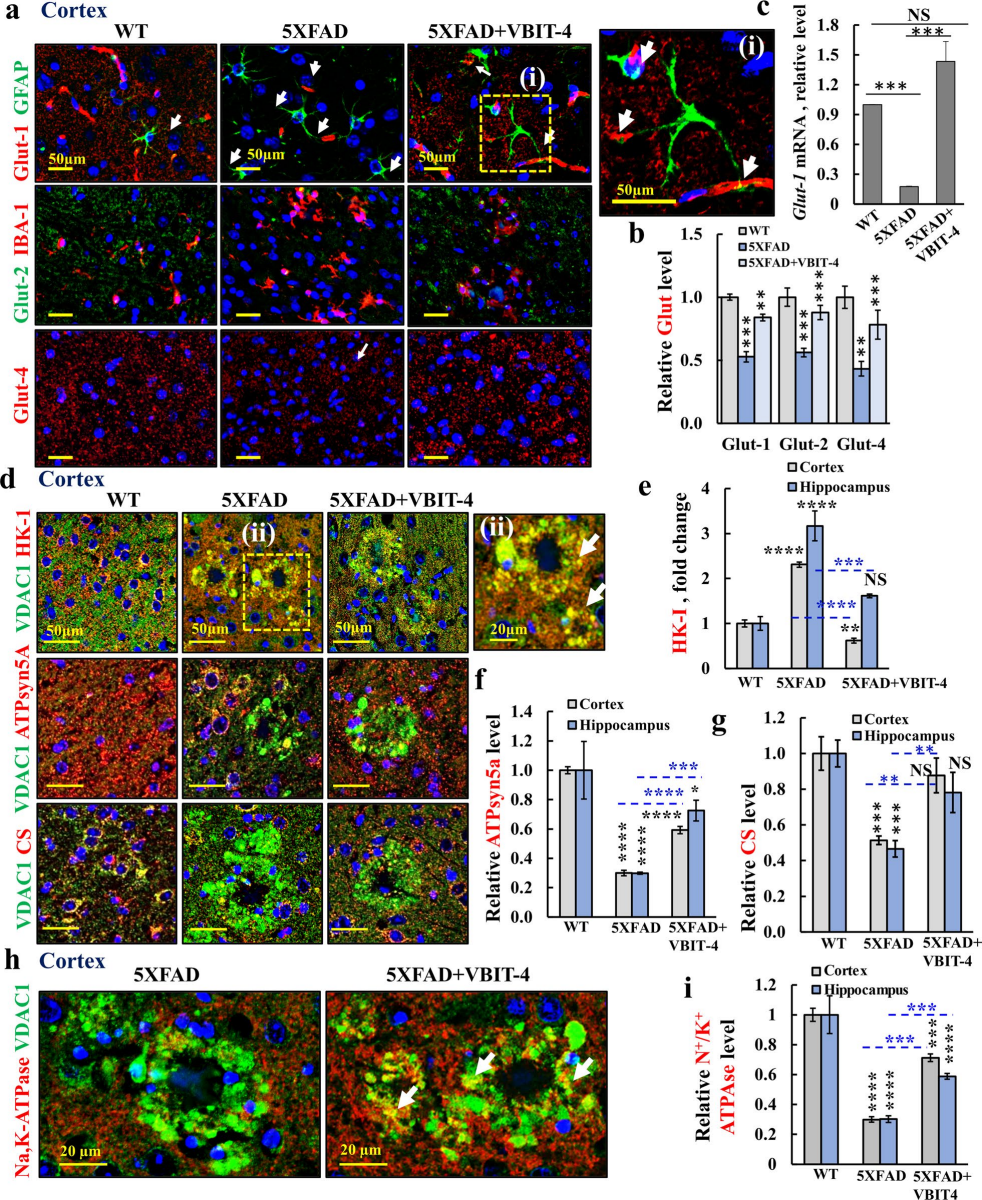
VBIT-4 treatment of 5 × FAD mice protects against cell metabolic impairments. **a** Confocal images of cortical sections from WT, untreated- and VBIT-4-treated-5 × FAD mice co-immunostained for glucose transporters: Glut-1 and GFAP, showing localization in astrocytes’ dendritic end-feet touching the blood vessels (white arrows), magnified in (i), Glut-2 co-stained with IBA-1 and Glut-4. **b** Glu-1,2,4 quantifications. (**c**) q-RT-PCR analysis of Glut-1 mRNA levels. **d**–**g** Cortical sections from the three groups co-stained for VDAC1 and HK-I, with a magnification of the selected area (ii), CS, or ATP synthase (ATPsyn5a) and their quantification in cortex and hippocampus (**e**–**g**). **h**, **i** Co-staining of Na,K-ATPase and VDAC1 and their quantification. Results show means ± SEM (*n* = 3), *P < 0.05, **P < 0.01, ***P < 0.001, ****P < 0.0001. *P-*value in blue color represents the significance of VBIT-4-treated relative to untreated 5 × FAD mice. NS, not significant

We found that the expression levels of Glut-1, Glut-2, and Glut-4 were downregulated in 5 × FAD mice, but not in VBIT-4-treated 5 × FAD mice (Fig. 7a, b, Additional file 1: Fig. S6). As expected, Glut-1 is expressed in the astrocytic dendritic end-feet near blood vessels, mediating glucose uptake across the blood–brain barrier endothelial cells (Fig. 7a(i), Additional file 1: Fig. S6a (i)). The expression of Glut-1 in the non-blood vessel compartment showed a two-fold decrease in 5 × FAD mice, and this was prevented by VBIT-4 treatment (Fig. 7a, b). Similar results were obtained at the mRNA level (Fig. 7c).

Glut-2, expressed in the microglia and other neuronal cells, was reduced in 5 × FAD mice, but not when treated with VBIT-4 (Fig. 7a, b, Additional file 1: Fig. S6b). Glut-4 is expressed in neurons, and its level was also reduced in 5 × FAD mice, which was prevented by VBIT-4 treatment (Fig. 7a, b). Similar results were obtained for the levels of Glut-1, Glu-2, and Glut-4 in the hippocampus (Additional file 1: Fig. S6c–f).

Considering impaired metabolism in AD [10, 36, 68] and VDAC1 regulating metabolism [21, 24], we also evaluated the expression of several metabolism-related proteins in 5×FAD mice and the effects of VBIT-4 on their expression levels (Fig. 7d–g). The expression levels of the glycolytic enzyme, hexokinase-I (HK-I), in the cortex and hippocampus were increased in the 5 × FAD mice, but the increase was prevented by VBIT-4 treatment (Fig. 7d, e, Additional file 1: Fig. S7). HK-I punctate staining was co-localized with VDAC1, including in the neuropils surrounding the Aβ plaques (Fig. 7d(ii)), suggesting that HK-I was mitochondrial bound. The expression levels of the Krebs cycle enzyme citrate synthase (CS) and ATP synthase (ATPsyn5a) were highly decreased in both the cortex and the hippocampus of 5 × FAD mice, but the decreases were prevented by VBIT-4 treatment (Fig. 7d, f, g, Additional file 1: Figs. S8, S9). Interestingly, in contrast to HK-I, which was co-localized with the overexpressed VDAC1 in the Aβ-plaque area, the mitochondrial proteins CS and ATPsyn5a were co-localized with VDAC1 in healthy neurons, but not in the neuropils around Aβ plaques (Additional file 1: Figs. S8, S9). This points to dysfunctional mitochondria around the Aβ-plaque areas, consistent with reduced metabolic activity [18].

Some VDAC1 was previously found to be localized to the plasma membrane (pl-VDAC1) [21], so we tested whether this might be the case also in the Aβ-plaque area by analyzing the co-localization of VDAC1 with the plasma membrane protein, Na,K-ATPase (Fig. 7h, i). The results showed that in the 5 × FAD mouse cortex, most of the VDAC1 around the Aβ-plaques was not co-localized with Na,K-ATPase (Fig. 7h).

The results also showed that the Na,K‐ATPase expression was decreased by about three folds (Fig. 7i), consistent with the reported decrease in AD patients and in a transgenic mouse AD model [73, 74]. Moreover, the decrease of Na,K‐ATPase staining in the VDAC1-overexpressing neuronal terminals surrounding the Aβ plaques was largely prevented by VBIT-4 treatment (Fig. 7h, i, Additional file 1: Fig. S10, circled plaque area). Similar results were obtained for the hippocampus (Additional file 1: Fig. S10c, d). Considering the function of Na,K-ATPase in maintaining the Na^+^ and K^+^ gradient across the plasma membrane, which is essential for maintaining resting membrane potential and hence neuronal excitability [75], the decrease in its expression levels points to decreased neuronal excitability in the 5 × FAD mice.

### VBIT-4 changes phenotypic properties of astrocytes and microglia

**Fig. 8:**
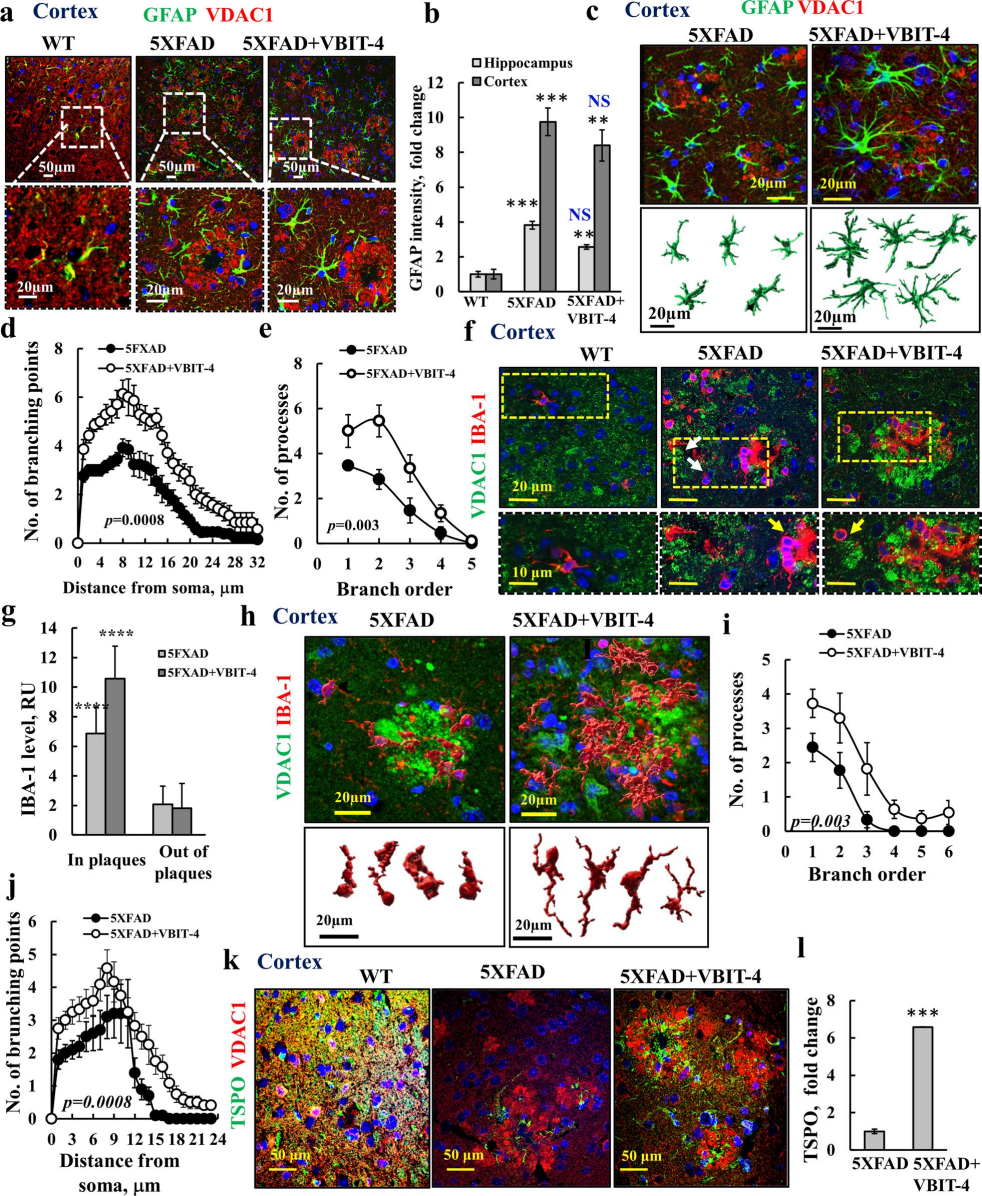
VBIT-4 treatment of 5 × FAD mice improves astrocyte and microglia morphology and activates microglia. **a** Confocal images of cortical sections from WT, untreated-, and VBIT-4-treated-5 × FAD mice co-immunostained for VDAC1 and GFAP. **b** Quantification of GFAP intensity in cortical and hippocampal sections. **c**–**e** Spinning disk microscopy 3D imaging of 50 μm cortical sections from 5 × FAD, and VBIT-4-treated-5 × FAD mice co-immunostained for GFAP and VDAC1 (**c**), analyzed using Imaris software for astrocyte 3D structures (**c**), number of branching points as a function of the distance from the soma (**d**) and number of processes for each branch order (**e**). **f** Cortical sections from WT, VBIT-4-treated, and untreated 5 × FAD mice were co-immunostained for VDAC1 and IBA-1. Higher magnifications of selected areas are shown. **g** Quantitative analysis of IBA-1 expression levels in and outside the Aβ plaques. **h**–**l** Spinning disk microscopy 3D imaging of 50 μm cortical sections from VBIT-4-treated- and untreated-5 × FAD mice stained for IBA-1 shown in 3D, as analyzed using Imaris software (**h**). Representative microglia structures in the Aβ plaques are shown at the bottom, and number of processes for each branch order (**i**) and the number of branching points as a function of the distance from soma (**j**). **k**, **l** Confocal images of cortical sections from WT, VBIT-4-treated, and untreated-5 × FAD mice co-immunostained for TSPO and VDAC1 (**k**) and quantification of staining intensity in the Aβ plaques (**l**). Results show means ± SEM (*n* = 3), ***P < 0.001, ****P < 0.0001. *P-*value in blue color represents the significance of VBIT-4-treated relative to untreated 5 × FAD mice. NS, not significant

Astrocytes support neurons by shuttling metabolites, secreting trophic factors, and regulating ion balance and pH [45]. Reactive gliosis has been shown in numerous models of AD and in AD patients [76]. In 5 × FAD mice, gliosis begins to occur around 2 months, and develops in parallel with plaque formation [45]. Indeed, IF staining showed that GFAP and glutamine synthase (GS), both expressed mainly in astrocytes [77], were increased, with GFAP increased by nine and three folds in the cortex and hippocampus of 5 × FAD mice, respectively, compared to the levels in WT (Fig. 8a, b; Additional file 1: S11a–c).

Since 3 × FAD mice show astroglia atrophy, as manifested by decreased surface areas and volumes of GFAP-positive cells relative to WT [78], we reconstructed astrocytes by confocal imaging of 50-μm-thick sections, and analyzed the GFAP-stained images within the Aβ plaques using Imaris. The results showed that astrocyte morphology in 5 × FAD mice was highly modified in comparison to the VBIT-4-treated mice (Fig. 8c–e). Astrocytes in the VBIT-4-treated mice had more processes with greater surface area, more branches, and more branching points along the processes (Fig. 8d, e; Additional file 1: Fig. S11d, e). The result suggested that astroglia distraction leads to early synaptic disorders, resulting in cognitive deficits in AD [78].

In AD, the microglia play important roles in Aβ clearance and neuroinflammatory response via secretion of pro-inflammatory cytokines [79]. In 5 × FAD mice, IF immunostaining showed that IBA-1, involved in phagocytosis by activated microglia, was threefold and sevenfold higher levels of IBA-1 outside and inside the Aβ plaques relative to WT (Fig. 8f-h, Additional file 1: Fig. S11f). Upon VBIT-4 treatment, IBA-1 levels were further increased about 11-fold in the Aβ plaques (Fig. 8g). No co-localization of IBA-1 and VDAC1 was observed, indicating that the cells overexpressing VDAC1 were not microglia.

Activated microglia undergo morphological changes and migrate to the site of injury [80]. 3D images of microglia within the Aβ plaques were reconstructed from IBA-1 images with Imaris. In the 5 × FAD mice, microglia had short and thick processes with an amoeboid-shape, whereas in the VBIT-4-treated mice the microglia were larger and had more and longer processes (Fig. 8h–j, Additional file 1: Fig. S11g, h). This finding suggests that VBIT-4 prevents damage to the microglia.

To further determine the effect of VBIT-4 on microglial activation, we analyzed the expression levels of the mitochondrial translocator protein (TSPO), as its upregulation is often accompanied with microglial activation and secretion of cytokines, and it is considered to be a marker of neuroinflammation [81] and AD severity [82]. The TSPO expression was found to be redistributed to be mainly in microglia around the Aβ plaques (Fig. 8k) and was increased in the 5 × FAD cortex and hippocampus (Fig. 8l, Additional file 1: Fig. S11i). Indeed, in 5 × FAD mice, TSPO was present mainly in Aβ plaques as visualized by VDAC1 staining, and its level was sevenfold higher in VBIT-4-treated mice (Fig. 8l).

Astrocytes play an important role in brain energy metabolism, mediating glucose uptake from blood vessels to neurons (Additional file 1: Fig. S6(i)) and microglial phagocytosis, which require a large amount of energy. The decreased expression of several metabolism-related enzymes (CS and cytochrome c oxidase [COX-IV]) in both astrocytes and microglia in 5 × FAD mice, was restored by VBIT-4 (Additional file 1: Figs. S12, S13), indicating that the astrocytic metabolic functions were restored in VBIT-4-treated mice.

### VBIT-4 treatment prevents exaggerated neuroinflammation

**Fig. 9:**
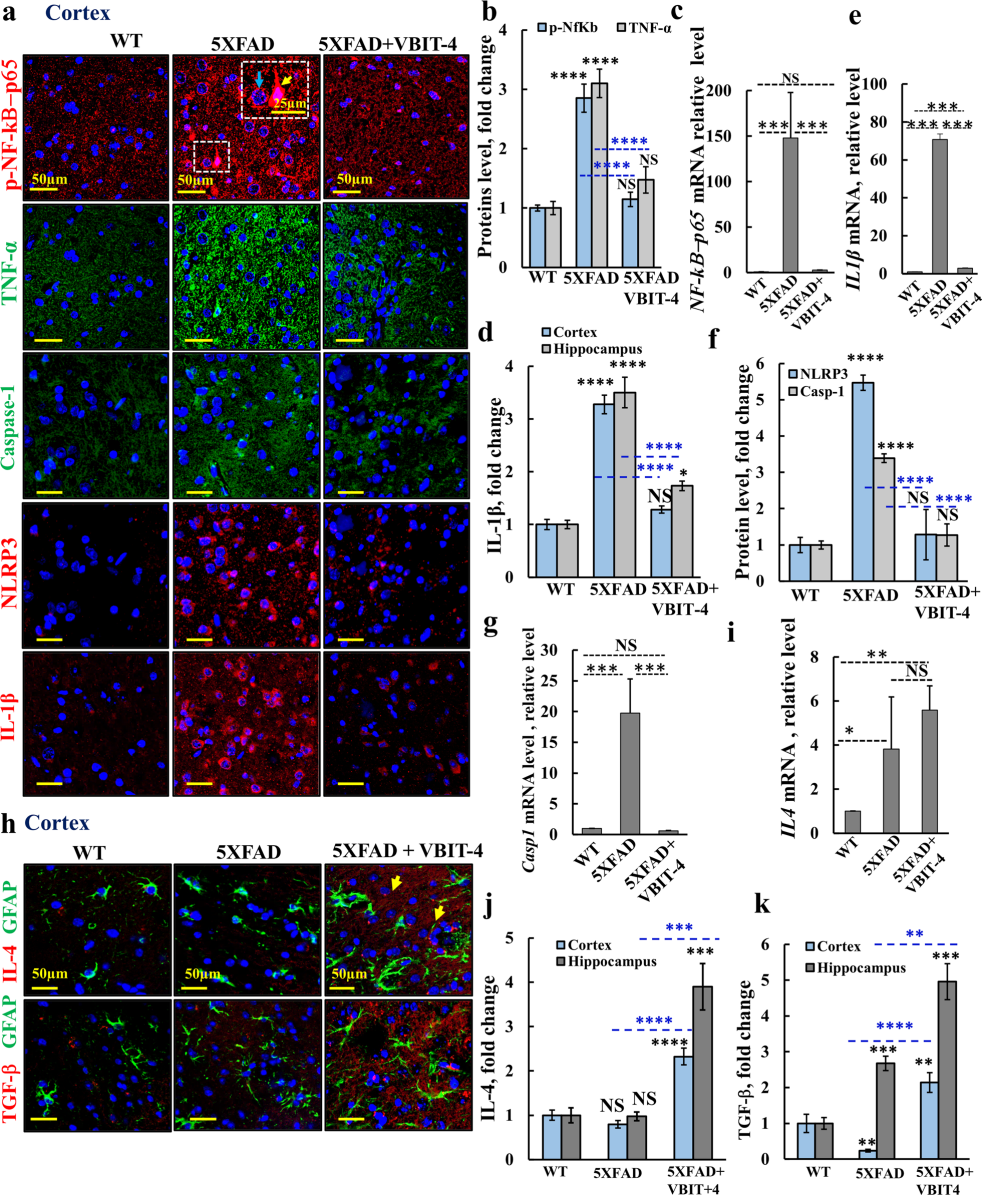
VBIT-4 reduces inflammation signaling and induces anti-inflammatory neuroprotective astrocytes and microglia in 5 × FAD mice. **a**,** b**, **d** Confocal images of cortical sections from WT, untreated-, and VBIT-4-treated-5 × FAD mice co-immunostained for p-NF-kB-p65, TNF-α, NRLP3, caspase-1 or IL1-β (**a**), and their quantifications (**b, d, f**). **c**, **e**,** g**, **i** q-RT-PCR analysis of NF-kB-p65, IL-1β, caspase-1 and IL-4 mRNA levels. **h** Confocal images of cortical sections from WT, untreated- and VBIT-4-treated 5 × FAD mice co-immunostained for GFAP and IL-4 or TGF-β, and their quantifications (**j**, **k**). *P-*value in the blue color represents the significance of VBIT-4-treated relative to untreated mice. Results show means ± SEM (*n* = 3), *P < 0.05, **P < 0.01, ***P < 0.001, ****P < 0.0001. *P-*value in blue color represents the significance of VBIT-4-treated relative to untreated 5 × FAD mice. NS, not significant

Next, we tested the effects of VBIT-4 on neuroinflammation associated with AD [83]. The transcription factor nuclear factor kappa B (NF-κB) functions in inflammation, and is implicated as a risk factor in AD [84]. Immunostaining showed that the phosphorylated, activated NF-κB-p65 (p-NF-κB-p65) was significantly increased in 5 × FAD mice compared to WT (Fig. 9a, b). In the cortex, p-NF-κB-p65 was mainly abundant in the cytoplasm and not as expected in the nuclei of neurons, particularly around neuronal nuclei with few stained nuclei (Fig. 9a, yellow and blue arrows) [85]. *NF-κB-p65* mRNA level was dramatically increased (~ 150-fold) in the 5 × FAD brain, and VBIT-4 prevented this increase (Fig. 9c).

As in the cortex, in the hippocampus, p-NF-κB-p65 staining was stronger in both the molecular and the granular layers where it was concentrated around the nuclei (Additional file 1: Fig. S14(i), arrows). In both the cortex and hippocampus of the 5 × FAD mice, VBIT-4 treatment reduced p-NF-κB-p65 expression to a level comparable to that in WT mice (Fig. 9a, b; Additional file 1: Fig. S14a,b). The activated p-NF-κB-p65 was found in astrocytes (Additional file 1: Fig. S14c), but also in neurons as shown for AD patients [86], where it co-localized with TUBB3, except in some of their processes (Additional file 1: Fig. S14d(ii) (iii)).

The levels of cytokines interleukin-1β (IL-1β) and tumor necrosis factor-α (TNF-α) regulated by NF-κB, are known to be increased in AD brains [87]. Consistent with this, our results showed increased TNF-α expression in 5 × FAD mice, while VBIT-4 prevented this elevation (Fig. 9a, b, Additional file 1: Fig. S15). Similarly, IL-1β expression was significantly increased in both cortex and hippocampus of 5 × FAD mice, while VBIT-4 prevented or attenuated this increase (Fig. 9a, d; Additional file 1: Fig. S15). Consistently, result of q-RT-PCR showed that *IL-1β* mRNA expression in the 5 × FAD mouse cortex was about 70-fold higher than that in WT mice, and VBIT-4 treatment greatly reduced this level (Fig. 9e). IL-1β expression levels were also increased in the hippocampus (Additional file 1: Fig. S15b).

NLRP3 (NOD-, LRR-, and pyrin domain-containing protein 3) acts as a sensor molecule, and together with the adaptor protein ASC (apoptosis-associated speck-like protein containing CARD) and pro-caspase-1 forms the NLRP3 inflammasome. The NLRP3 inflammasome is critical for the innate immune system [88], and is associated with neuroinflammation in AD [89]. We found that NLRP3 was highly expressed in both the cortex and hippocampus of 5 × FAD mice, while VBIT-4 treatment prevented the increase (Fig. 9a, f; Additional file 1: Fig. S16a,b,f).

In the activated inflammasome, caspase-1 is activated, converting proinflammatory cytokines such as pro-IL-1β into active forms [90]. The expression level of caspase-1 was highly increased in the cortex and hippocampus of 5 × FAD mice, but not in VBIT-4-treated mice (Fig. 9a, f;. Additional file 1: Figs. S16 and S17). This was also confirmed at the mRNA level (Fig. 9g). Thus, VBIT-4 protects against neuroinflammation.

The increase in activated microglia and decrease in pro-inflammatory agents induced by VBIT-4 treatment led us to consider the transition of microglia and astrocytes from a pro-inflammatory/neurotoxic (M1) to an anti-inflammatory/neuroprotective (M2) phenotype [91]. As the neuroprotective astrocytes and microglia are promoted by IL-4, IL-13, IL-10, and TGF-β [91], we compared the expression levels of IL-4 and TGF-β in VBIT-4-treated and untreated 5 × FAD mice. The results show that in 5 × FAD mice IL-4 levels were as in the WT in the microglia of both cortex and hippocampus, while TGF-β was decreased over 2 folds in the cortex and increased 3 folds in the hippocampus of 5 × FAD mice. However, upon VBIT-4 treatment, IL-4 and TGF-β were increased about 2- and 5-folds in the cortex and hippocampus, respectively (Fig. 9h–k, Additional file 1: Figs. S18, S19).

## Discussion

AD neuropathology is multifactorial, involving numerous biological pathways, including neuro-metabolic mitochondrial dysfunctions, impaired Ca^2+^ homeostasis, Aβ and hyperphosphorylated-tau accumulation, and neuroinflammation [92]*.*

**Fig. 10:**
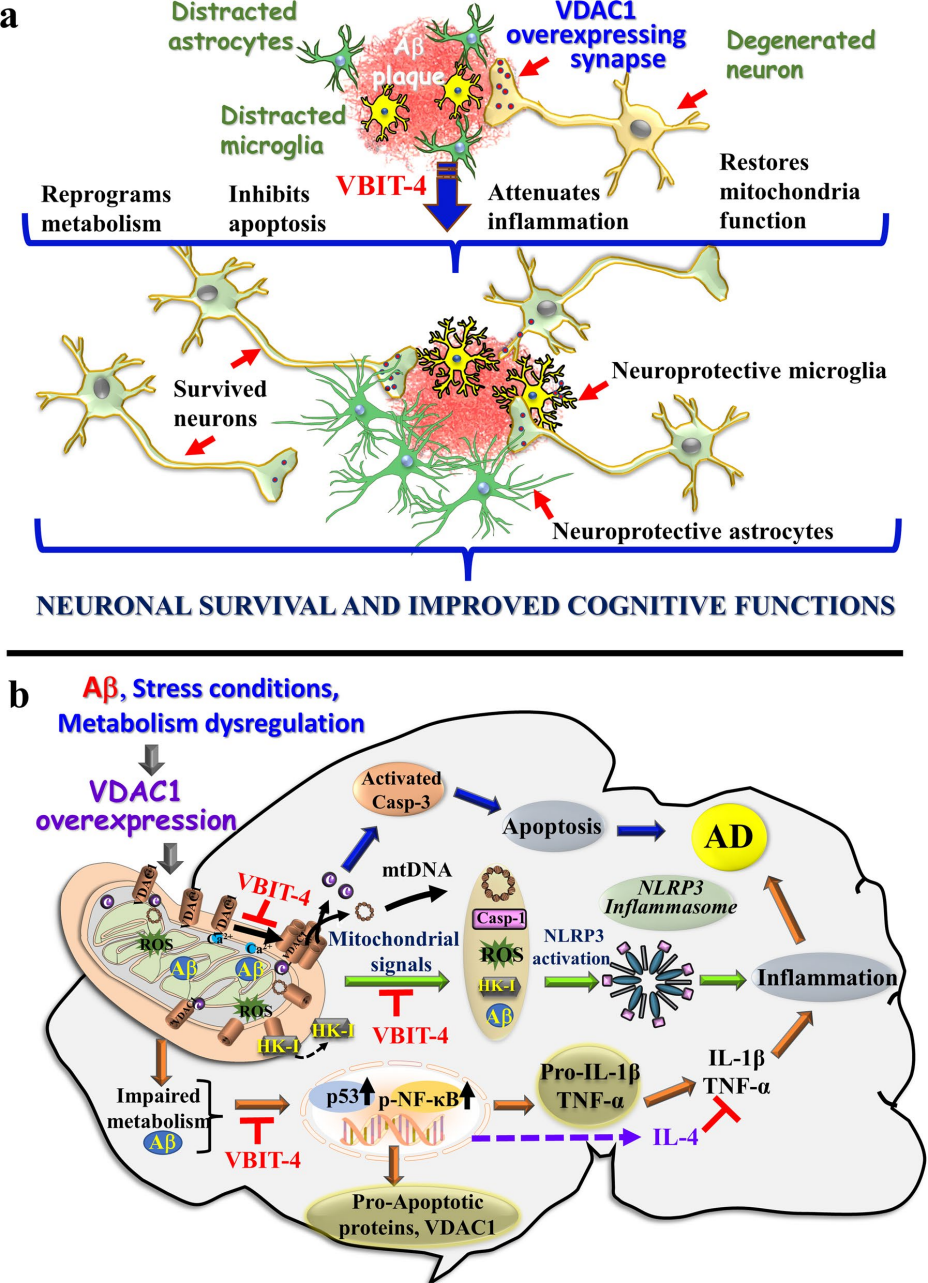
Schematic model of mitochondria and VDAC1 functions in apoptosis, inflammasome activation, and inflammatory response leading to AD, and their prevention by VBIT-4 in 5 × FAD mice. **a** Aβ plaques contain neurons that overexpress VDAC1 (red circles), astrocytes (green cells), and activated microglia (yellow cells). Following VBIT-4 treatment, the cells undergo metabolic reprograming to OXPHOS and restored mitochondria functions; inhibited apoptosis and inflammation, leading to neuronal survival and improved astrocyte and microglia morphology and their activation to the neuroprotective phenotypes. All lead to modulated neuronal survival on and improved cognitive function. **b** Molecular mechanisms proposed for mitochondria and VDAC1-mediated AD pathology and their modulation by VBIT-4. VDAC1 is involved in numerous mitochondria-associated functions, including cell metabolism, inflammation, and apoptosis, which are all impaired in AD. VDAC1 overexpression induced by Aβ triggers mitochondrial dysfunction, apoptosis, and immuno-inflammation via three major signaling pathways: I. Apoptosis pathway (blue arrows): Apoptosis is induced by oligomerization of the overexpressed VDAC1 (induced by Aβ) to form a large channel mediating Cyto *c* release, apoptosome formation, caspase-3 activation, and apoptotic cell death, contributing to AD. VBIT-4 inhibits VDAC1 oligomerzation, thereby apoptosis. II. Mitochondrial signaling (green arrows): Mitochondrial dysfunction (resulting from Aβ toxicity) increases ROS levels, as well as expression of HK-I, pro-caspase-1, Ca^2+^ and TSPO. This facilitates the NRLP3 inflammasome assembly and activation, leading to pro-caspase-1 activation and pro-inflammatory cytokine maturation, thereby initiating an inflammatory cascade associated with AD development. VBIT-4 prevents mitochondrial dysfunction, and thereby the mitochondria signaling to inflammasome activation. III. Impaired metabolism (orange arrows): Mitochondria decreased metabolism, connected to epigenetic, alters gene expression [141], increasing NF-kB levels, thereby enhancing inflammatory factor levels associated with neuroinflammation. These, together with Aβ inducing VDAC1 and p53 expression stimulating apoptosis [54–56], lead to AD development. VBIT-4 increases the expression of IL-4 and TGF-β, suppressing NLRP3-dependent caspase-1 activation. In this model, the overexpression and oligomerization of VDAC1 acts as a common functional junction for all these activated pathways in AD. Hence, inhibition of VDAC1 oligomerization by VBIT-4 suppresses apoptosis while restoring metabolism and suppressing NLRP3-dependent caspase-1 activation and the subsequent IL-1β and TNF-α activation, resulting in inhibition of inflammation. VBIT-4 inhibits mtDNA release into the cytosol, and thereby NLRP3 activation and IL-1β production. In addition, VBIT-4 upregulates IL-4 and TGF-β to modulate neuroprotective phenotypes of astrocytes and microglia. Collectively, these effects of VBIT-4 lead to prevention of AD pathology including cognitive dysfunction

This study presents a new approach to targeting the mitochondrial gatekeeper VDAC1 using a newly developed VDAC1-interacting small molecule, VBIT-4, to treat AD pathology in a mouse model. We demonstrated that Aβ induces VDAC1 overexpression in the neuropils surrounding the Aβ plaques, suggesting that VDAC1 is responsible for mitochondrial dysfunction, leading to apoptosis and neuroinflammation. We conclude that VBIT-4 prevented apoptosis and neuroinflammation and switched glia to neuroprotective phenotypes, finally leading to restoration of cognitive function (Fig. 10).

Significant strategic efforts have been focused on reducing production or increasing clearance of Aβ to treat AD, but all have thus far failed [3, 93–95]. In agreement with recent views that alternative perspectives to the Aβ-cascade hypothesis should be followed with a focus on mitochondria as a major target for treating AD [4, 12–17, 20, 96], our results argue against Aβ accumulation being the primary event leading to a cascade of effects that ultimately result in neuronal damage. We found that VBIT-4 prevented AD-associated pathophysiological changes, including neuronal loss, neuroinflammation, and neuro-metabolic dysfunctions, and the VBIT-4-treated 5 × FAD mice manifested with normal cognitive functions, even when there were no significant changes in phosphorylated-Tau and a ~ 20% decrease in Aβ-plaque load.

Recent observations have suggested an association between AD and PrPC levels [58, 97]. PrPC, which is proposed to be neuroprotective [59], was highly decreased in 5 × FAD mouse brains compared to that in the WT, while VBIT-4 restored the level to be even higher than the WT. VBIT-4 had no effect on the levels of amylin proposed to be associated with AD [61].

We demonstrated that Aβ induces VDAC1 overexpression in the neuropils surrounding the Aβ plaques and propose that this overexpressed VDAC1 is responsible for mitochondrial dysfunction, leading to neuroinflammation and apoptosis. VBIT-4, by inhibiting VDAC1 oligomerization and related processes, protects against the loss of cognitive functions in 5 × FAD mice (Fig. 10).

### Aβ-induced VDAC1 overexpression leads to neuronal cell death

It is proposed that in AD, progressive brain neuronal loss due to apoptotic cell death accumulates over the years, and this impacts learning, memory, and general cognition. While the mechanisms responsible for cell death in AD are not fully understood, it is clear that apoptosis is the primary contributor to AD neurodegeneration [16–18, 98]. The proposed mitochondrial cascade has recently gained support by various studies, suggesting that targeting and preventing this cascade is a promising approach to treating AD [12–15, 99, 100].

Here, we showed that Aβ added to a primary neuronal cell culture induced an overexpression of VDAC1. In the 5 × FAD mouse model, VDAC1 was specifically overexpressed around the Aβ plaques in the neuronal terminals, suggesting that this overexpression may lead to VDAC1 oligomerization and formation of a large channel that allows pro-apoptotic proteins from the mitochondria to subsequently trigger apoptosis [21, 23–25, 35, 101]. VBIT-4, by inhibiting VDAC1 oligomerization, prevented mitochondria dysfunction and apoptosis, and thereby loss of neurons in 5 × FAD mice. VDAC1 overexpression in the neuronal terminals surrounding the Aβ plaques in this study, and in AD brains, APP mouse brains, and Aβ-treated cells reported in previous studies [31, 32, 102], may result from VDAC1 promoter activation by Aβ. Indeed, the DNA consensus sequence that binds to Aβ [54] is also present in the VDAC1 promoter. Alternatively, it has been shown that intracellular Aβ can translocate to the nucleus and activate promoters of genes implicated in AD pathogenesis such as *APOE*, *APP*, *BACE1* [54, 55], and *p53* [56]. p53 expression was elevated in the brains of sporadic AD patients [103] and was shown here to be elevated by Aβ in primary neuronal cultures. Thus, VDAC1 overexpression may also be mediated by Aβ-induced p53 expression. It has been suggested that p53 modulates VDAC1 oligomerization [104, 105]; thus, it may promote VDAC1 oligomerization in the neuronal terminals overexpressing VDAC1.

Neuronal cell death has been demonstrated in AD patients due to mitochondria-mediated apoptosis [11], as reflected in the release of  Cyto *c* from the mitochondria and caspase activation [16, 17, 106]. Here, we showed that in the 5 × FAD brain, caspase-3 was activated, and TUNEL staining was increased, which represents DNA degradation.

VBIT-4 administered to the AD-like mice over a 4-month period prevented cell death, and thereby neuronal loss, as shown by the restored expression of the neuronal markers, synaptophysin, TUBB3, and PSD-95.

A link between VDAC1 expression levels and AD has also recently been demonstrated in double mutant (VDAC1 +/−/TAU) transgenic mice, showing that a partial reduction in VDAC1 levels rescues motor coordination and learning and spatial memory, and enhances mitophagy, autophagy, and synaptic activities [107]. In addition, in the same mouse model*,* reduced VDAC1 was shown to maintain mitochondrial dynamics and enhance mitochondrial biogenesis [108].

### VBIT-4 prevents mitochondrial dysfunction and metabolism impairment, and immunometabolism dysregulation in 5 × FAD mice

The brain’s energy demand is very high, with cognitive functions accounting for a large proportion of the required energy, produced mainly by mitochondria. Therefore, it is not surprising that metabolic dysregulation contributes to the progression of AD [8, 10, 36, 109]. Moreover, glucose metabolism is significantly decreased in AD patients 10 years before the appearance of clinical symptoms [5], with reductions of Glut-1 and Glut-3 in AD patients’ brains [71, 72].

Here, we show that the levels of Glut-1 in astrocytes, Glut-2 in microglia and neurons, and the insulin-sensitive Glut-4 found in distinct subsets of neurons [70], were decreased in 5 × FAD mice, but not in VBIT-4-treated mice, suggesting restoration of the glucose supply.

Mitochondrial dysfunction, constituting early AD pathogenesis, emerges before Aβ plaques appear in the brain, and is associated with reduced metabolism and increased ROS production [13–15, 18, 20, 99, 100]. Our results showed decreased expression of metabolism-related mitochondrial proteins such as CS and ATPSyn5a in 5 × FAD mice, which was prevented by VBIT-4, suggesting that it restores inactive mitochondrial oxidative phosphorylation (OXPHOS).

In the brain, the large amounts of ATP consumed are mostly required for maintenance of the ionic gradients maintained by Na,K‐ATPase, which underlies the resting and action potentials involved in nerve impulse propagation and neurotransmitter release. Thus, it is not surprising that Na,K-ATPase dysfunction is associated with neurological diseases such as depression, mood disorders, stress, AD, learning and memory impairment, neuronal hyperexcitability, and epilepsy.

Consistent with the reported decrease in Na,K‐ATPase expression in AD patients and a transgenic mouse model of AD [73, 74], we showed here that the expression levels of Na,K‐ATPase, a major energy consumption protein, were markedly decreased in the 5 × FAD brains and almost completely absent in the neuropils around Aβ plaques. As Na,K-ATPase maintains the resting membrane potential and hence neuronal excitability [75], decreased levels of Na,K-ATPase are expected to reduce synaptic transmission, as proposed previously [74]. Remarkably, VBIT-4 treatment prevented the decrease of Na,K-ATPase expression in 5 × FAD mice. Thus, VBIT-4 by restoring mitochondrial functions throughout the neuropil, reflected in the expression of metabolism-related proteins, allows Na,K-ATPase to utilize ATP and establish the electrochemical gradient across the plasma membrane, and thereby, maintain information processing.

Finally, compelling evidence implicates a role for mitochondria in inflammation via regulation of the NLRP3 inflammasome, proposing it as a site of initial inflammasome nucleation [110]. Mitochondrial d**y**sfunction, reactive oxygen [111], release of mtDNA involving VDAC1 oligomerization, and inhibition by VBIT-4 [26, 27] have all been linked to NLRP3 inflammasome activation. In addition, HK, a metabolic enzyme, is linked to inflammatory signaling by binding to VDAC1, and it participates in a cascade of events on the mitochondrial surface that promotes NLRP3 inflammasome assembly and activation [112]**.**

The NLRP3 inflammasome as a signaling complex central to inflammation in multiple disease processes including atherosclerosis, myocardial infarction, diabetes, colitis and AD, among others [113, 114], is further discussed below.

### VBIT-4 promotes neuroprotective phenotypes of astrocytes and microglia and restores cognitive functions

Increasing evidence suggests that AD pathogenesis involves not only neurodegeneration, but also immunological signaling, with neuroinflammation being one of the hallmarks of AD [115], and this is associated with neuronal death [116]. Reactive microglia and astrocytes play a key role in neuroinflammation, and in AD, they contribute to the dysfunction and deprivation of synapses and to the neuronal death [117].

Upon certain conditions (such as ROS accumulation), microglia and astrocytes in the brain release inflammatory cytokines such as IL-1β, TNF-α, IL-6, and TGF-β near Aβ plaques [118]. It has been shown that IL-1β drives amplified responses in primed astrocytes and neuronal network dysfunction [119]. Also, elevated serum TNF-α is associated with an increased rate of cognitive decline in patients with AD [120]. Finally, it has been shown that Aβ mediates the NLRP3 inflammasome priming and activation. NF-κB promotes the transcription of NLRP3 and pro-IL-1β, followed by oligomerization of NLRP3 and its interaction with ASC, which recruits caspase-1, subsequently leading to IL-1β and TNF-α activation [83] (Fig. 10b). Indeed, NLRP3 inflammasome has been proposed as a novel therapeutic target for AD [121].

Our results show that the increased expression/activation of p-NF-κB-p65, NLRP3 inflammasome, caspase-1, IL-1β, and TNF-α in 5 × FAD mice was prevented by VBIT-4 treatment. The effect of VBIT-4 may be due to its restoration of OXPHOS, as in AD models, oxidative phosphorylation reduces proinflammatory cytokine production [122].

Microglia and astrocytes are considered to have multiple reactive phenotypes related to the type and stage of neurodegenerative diseases, being neurotoxic/pro-inflammatory or anti-inflammatory/neuroprotective/”healing” [123, 124]. Extracellular Aβ and p-Tau drive microglia and astrocytes into their pro-inflammatory phenotypes. The pro-inflammatory microglia (M1) secret factors such as IL-1β, TNF-α, IL-6, and NO to recruit additional cells and to activate astrocytes into the pro-inflammatory phenotype (A1), pointing to an astrocyte–microglia cross-talk. VBIT-4 dramatically decreased the expression of the pro-inflammatory factors. The anti-inflammatory/neuroprotective astrocytes (A2) and microglia (M2) are promoted by anti-inflammatory cytokines IL-4, IL-13, IL-10, and TGF-β, which lead to the release of diverse factors including FIZZ1 and arginase-1 [91]. Remarkably, VBIT-4 increased the expression of IL-4 and TGF-β. Importantly, IL-4 suppresses TNF-α, IL-6, and NO effects and NLRP3-dependent caspase-1 activation and the subsequent IL-1β secretion, and inhibits subcellular redistribution of NLRP3 into mitochondria [125]. The astrocytic TGF-β is involved in the inhibition of NF-κB signaling, thus, reducing neuroinflammation [91]. Moreover, neurons, under certain conditions, transcriptionally upregulate IL-4 through Ca^2+^-calcineurin-phosphatase, to modulate microglial neuroprotective phenotype [126]. VBIT-4 restores Ca^2+^ homeostasis [41] and induces the microglial neuroprotective phenotype, also resulting in increased Aβ clearance as reflected in the observed decrease in Aβ load. In addition, VBIT-4 inhibits mtDNA release into the cytosol [26], thereby inhibiting NLRP3 modulation and IL-1β production [127]. Thus, VBIT-4 promotes the anti-inflammatory/neuroprotective astrocytes and microglial phenotypes.

The molecular basis of VBIT-4-induced neuroprotective phenotypes of microglia and astrocytes also involves metabolism modulation. The cellular metabolism from mitochondrial OXPHOS to anaerobic glycolysis acts as a switch for the change of glial phenotype from neuroprotective to neuroinflammatory [122, 128]. VBIT-4 reverses microglial and astrocytic metabolism back to mitochondrial OXPHOS, leading to their loss of neurotoxic functions and gain of neuroprotective functions, including neuroinflammation attenuation. This also enhances the microglia-mediated Aβ clearance, as reflected in the decrease in the area occupied by Aβ plaques in VBIT-4-treated mice.

The effects of VBIT-4 on neurons, astrocytes and microglia lead to the improvement of cognitive functions in 5 × FAD mice. The astrocyte–neuron interactions, known as tripartite synapses, are associated with the alterations of morphological dynamics of astrocytes with the number of astrocytic processes increased, and disruption of the interaction is linked to impairments in learning and memory in 5 × FAD mice [129]. Here, we showed that VBIT-4 improved astrocyte morphology and OXPHOS activity.

Additionally, to maintain the connections with neurons, activated astrocytes release many neurotransmitters including glutamate, whose levels were reduced in 5 × FAD mice due to decreased expression of glutamine synthase, leading to cognitive dysfunction. VBIT-4 treatment increased GS levels in astrocytes, thereby, restoring glutamate release and cognitive functions.

Thus, neuroinflammation has a prominent role in the pathogenesis of AD. Considering mitochondrial function in inflammasome activation [111], and VDAC1 being the mitochondria governor, VBIT-4 by interacting with VDAC1, inhibits apoptosis, reduces ROS production, cellular Ca^2+^ [41] and inflammatory response, and restores cell metabolism – all these lead to alleviated AD symptoms.

### A new uncovered strategy to treat AD by targeting the mitochondrial protein VDAC1

AD is a multifactorial disease [92], with its etiology and pathogenesis based on amyloid cascade and tau-hyperphosphorylation hypotheses [2]. However, high-profile clinical trials, including 99% of Phase 2–3 clinical trials of AD, have failed over the years [93, 130]. The drugs currently approved for AD treatment include cholinesterase (ChE) inhibitors (galantamine rivastigmine), donepezil [131], and memantine, a non-competitive *N*-methyl-*D*-aspartate receptor antagonist [132]. Mitochondrial bioenergetics [133], inhibitors of NLRP3 inflammasome [121], and insulin intranasal [134] have also been proposed as therapeutic strategies in AD. Yet, new approaches with novel mechanistic strategies to combat AD are urgently needed. Here, we showed that by using the VDAC1-interacting molecule, VBIT-4, it is possible to mitigate brain pathology in a mouse model of AD.

Considering mitochondrial dysfunction as a critical factor in AD pathogenesis [13–17, 99, 100], we present here results supporting the mitochondria–VDAC1 axis as a new target for AD therapies. VDAC1, a mitochondrial activity governor, controls cell metabolism, energy production, Ca^2+^ homeostasis, ROS production, lipid oxidation, and apoptosis [21, 23–25, 35].

VDAC1 overexpressed in the AD brain is tightly associated with apoptosis induction, and VBIT-4. Targeting this VDAC1 prevents its oligomerization at an **early stage** of apoptosis, and ameliorates all tested AD-associated pathways (Fig. 10). Currently, there are no apoptosis inhibitor-based therapies for diseases associated with enhanced apoptotic cell death such as AD. In contrast, the existing apoptosis inhibitors target proteins at the **end step** of apoptosis, such as caspase inhibitors. The results presented here suggest that the VDAC1-oligomerization-specific inhibitor, VBIT-4, by preventing mitochondrial dysfunction and apoptosis, prevents neuronal cell death, neuroinflammation, and metabolic destruction, hence, restoring cognitive activity in an AD-like mouse model.

Finally, VBIT-4 can reach the brain, and when given in drinking water, it showed a PK of 7.6 h, indicating a stable metabolic profile without mortality or clinical signs. Thus, VBIT-4 is a promising new VDAC1-based drug candidate for the treatment of AD, and it may effectively treat other neurodegenerative diseases.

In this respect, accumulating results suggest that VDAC1 overexpression is a common threat in diseases and point to tight coupling between VDAC1 overexpression, VDAC1 oligomerization, apoptosis induction, and pathological states. These have been shown for neurodegenerative diseases [32, 36, 135], type 2 diabetes [42], lupus [26], colitis [43], acute liver injury [136], rheumatoid arthritis [137], and spinal cord injury [138]. Myocardia of humans and rats [139] and T cells of COVID-19 patients express increased VDAC1 levels [140]. In some of these diseases, VBIT-4 or VBIT-12 has been demonstrated to attenuate disease pathology.

## Conclusions

VBIT-4 alleviates the AD pathology primarily by preventing neuronal loss due to apoptosis as triggered by Aβ-induced VDAC1 overexpression. In 5 × FAD mice, VBIT-4, by preventing VDAC1-associated cell death, protects against neuronal and synaptic loss, restores metabolism in neurons and glia, prevents morphological distraction of astrocytes and microglia, and attenuates neuroinflammation by activating the neuroprotective phenotypes of glia. VBIT-4 prevents cognitive decline in the 5 × FAD mice, as evaluated using several behavioral assessments of cognitive functions.

VBIT-4, by restoring mitochondria functions throughout the neuropil, reflected in the expression of metabolism-related proteins and the expression of glutamine synthetase and Na,K-ATPase, allows the establishment of the membrane potential at the plasma membrane, and results in improved cognitive functions.

Interestingly, VBIT-4 treatment has no significant effects on phosphorylated-Tau and only causes a slight decrease of Aβ deposits in the 5 × FAD mouse model.

In summary, our study suggests that targeting mitochondrial dysfunction with its gatekeeper VDAC1 may represent a new target for AD therapeutic intervention, and VBIT-4 may be a new drug candidate for AD treatment.

## Supplementary Information

**Additional file 1. Materials and Methods. Table S1.** Real-time PCR primers used in this study.** Table S2.** Antibodies used in this study.** Table S3.** VDAC1 promoter sites matching sequence profiles generated from AβID decamers identified by Maloney and Lahiri.** Table S4.** ADME/PK Profile – Summary.** Fig. S1.** Aβ induces VDAC1 overexpression, oligomerization, and apoptotic cell death.** Fig. S2.** VBIT-4 improves cognition, learning, and memory performance in the 5×FAD mouse model and had no effect on WT mice.** Fig. S3.** Effect of VBIT-4 treatment on the levels of amylin expression in the 5 × FAD brain.** Fig. S4.** VBIT-4 treatment of 5 × FAD mice prevents neuronal and synaptic loss.** Fig. S5.** VBIT-4 treatment of 5×FAD mice protects against cell death.** Fig. S6.** VBIT-4-treatment attenuated a decrease in the expression of glucose transporters in 5 × FAD mice.** Fig. S7.** HK-I is overexpressed and co-localized with VDAC1 in the neuropils surrounding the Aβ plaques.** Fig. S8.** VBIT-4 treatment of 5 × FAD mice prevented the decrease in citrate synthase expression.** Fig. S9.** VBIT-4 treatment of 5 × FAD mice prevented a decrease in ATP synthase expression.** Fig. S10.** VBIT-4 treatment of 5 × FAD mice prevented a reduction in NaK-ATPase expression.** Fig. S11.** VBIT-4 treatment of 5 × FAD mice improves astrocyte and microglia morphology.** Fig. S12.** VBIT-4 increases CS and ATP synthase expression in astrocytes in 5 × FAD mice.** Fig. S13.** VBIT-4 increases CS and ATP synthase expression in microglia in 5 × FAD mice.** Fig. S14.** VBIT-4 reduces p-NF-kB —p65 and TNF-α expression in 5 × FAD mice.** Fig. S15.** VBIT-4 reduces IL1-β expression in 5×FAD mice.** Fig. S16.** VBIT-4 reduces NRLP3 and caspase-1 expression in 5 × FAD mice.** Fig. S17.** VBIT-4 reduces IBA-1 and caspase-1 expression in 5 × FAD mice.** Fig. S18.** VBIT-4 increases the expression of IL-4 and TGF-β in microglia of 5 × FAD mice.** Fig. S19.** VBIT-4 increases the expression of IL-4 TGF-β in astrocytes of 5 × FAD mice.

## Data Availability

All are included in the article and the supplementary data.

## Abbreviations

AD: Alzheimer's disease
Aβ ID: Aβ interacting domain
APP: Amyloid precursor protein
ASC: Apoptosis-associated speck-like protein containing CARD
COX-IV: Cytochrome c oxidase
CS: Citrate synthase
Cyto * c*: Cytochrome *c*
DAB: 3,3-Diaminobenzidine
DMSO: Dimethyl sulfoxide
GFAP: Glial fibrillar acidic protein
Glut: Glucose transporters
GS: Glutamine synthase
HK-I: Hexokinase-I
IBA-1: Ca^2+^-binding adaptor molecule-1
IHC: Immunohistochemistry
IF: Immunofluorescence
IL: Interleukin
mtDNA: Mitochondrial DNA
NeuN: Neuronal nuclear protein
NRLP3: Nucleotide-binding domain (NOD)-like receptor protein 3
OXPHOS: Oxidative phosphorylation
PBST: Phosphate buffer saline containing 0.1% Triton-X100
PLGA: Poly lactic-co-glycolic acid
PrPc: Cellular prion protein
PSD-95: Post-synaptic density protein-95
ROS: Reactive oxygen radical
TGF: Transforming growth factor
TNF: Tumor Necrosis Factor
TSPO: Mitochondrial translocator protein
TUBB3: Class III beta-tubulin
TUNNEL: Terminal deoxynucleotidyl transferase biotin-dUTP nick end labeling
VDAC1: Voltage-dependent anion channel 1
