# Correction: Targeting the overexpressed mitochondrial protein VDAC1 in a mouse model of Alzheimer’s disease protects against mitochondrial dysfunction and mitigates brain pathology

**DOI:** 10.1186/s40035-023-00335-3

**Published:** 2023-01-11

**Authors:** Ankit Verma, Anna Shteinfer-Kuzmine, Nikita Kamenetsky, Srinivas Pittala, Avijit Paul, Edna Nahon Crystal, Alberto Ouro, Vered Chalifa-Caspi, Swaroop Kumar Pandey, Alon Monsonego, Noga Vardi, Shira Knafo, Varda Shoshan-Barmatz

**Affiliations:** 1 Department of Life Sciences, Ben-Gurion University of the Negev, 84105 Beer-Sheva, Israel; 2 National Institute for Biotechnology in the Negev, Ben-Gurion University of the Negev, 84105 Beer-Sheva, Israel; 3 Achva Academic College, 79804 Shikmim, Israel; 4 Department of Physiology, Faculty of Health Sciences, Ben-Gurion University of the Negev, 84105 Beer-Sheva, Israel; 5 Ilse Katz Institute for Nanoscale Science and Technology, Ben-Gurion University of the Negev, 84105 Beer-Sheva, Israel; 6 The Shraga Segal Department of Microbiology, Immunology, and Genetics, Faculty of Health Sciences, Ben-Gurion University of the Negev, 84105 Beer-Sheva, Israel; 7 Present Address: NeuroAging Group (NEURAL), Clinical Neurosciences Research Laboratory (LINC), Health Research Institute of Santiago de Compostela (IDIS), 15706 Santiago de Compostela, Spain

**Correction: Translational Neurodegeneration (2022) 11:58** 10.1186/s40035-022-00329-7

Following publication of this article [1], the authors identified an error in the family name of Alon Monsonego.

The incorrect author name is: Alon Monsengo.

The correct author name is: Alon Monsonego.

The author group has been updated above and the original article [1] has been corrected.

## References

[1] Verma (2022). Targeting the overexpressed mitochondrial protein VDAC1 in a mouse model of Alzheimer’s disease protects against mitochondrial dysfunction and mitigates brain pathology. Transl Neurodegener.

