# Supplementary material for: Targeting the overexpressed mitochondrial protein VDAC1 in a mouse model of Alzheimer’s disease protects against mitochondrial dysfunction and mitigates brain pathology
Source: Transl Neurodegener. 2022 Dec 28;11:58. doi: 10.1186/s40035-022-00329-7 (PMC9795455; doi:10.1186/s40035-022-00329-7)
Supplement: Supplementary file 1 — Additional file 1. Materials and Methods. Table S1. Real-time PCR primers used in this study. Table S2. Antibodies used in this study. Table S3. VDAC1 promoter sites matching sequence profiles generated from AβID decamers identified by Maloney and Lahiri. Table S4. ADME/PK Profile – Summary. Fig. S1. Aβ induces VDAC1 overexpression, oligomerization, and apoptotic cell death. Fig. S2. VBIT-4 improves cognition, learning, and memory performance in the 5×FAD mouse model and had no effect on WT mice. Fig. S3. Effect of VBIT-4 treatment on the levels of amylin expression in the 5 × FAD brain. Fig. S4. VBIT-4 treatment of 5 × FAD mice prevents neuronal and synaptic loss. Fig. S5. VBIT-4 treatment of 5×FAD mice protects against cell death. Fig. S6. VBIT-4-treatment attenuated a decrease in the expression of glucose transporters in 5 × FAD mice. Fig. S7. HK-I is overexpressed and co-localized with VDAC1 in the neuropils surrounding the Aβ plaques. Fig. S8. VBIT-4 treatment of 5 × FAD mice prevented the decrease in citrate synthase expression. Fig. S9. VBIT-4 treatment of 5 × FAD mice prevented a decrease in ATP synthase expression. Fig. S10. VBIT-4 treatment of 5 × FAD mice prevented a reduction in NaK-ATPase expression. Fig. S11. VBIT-4 treatment of 5 × FAD mice improves astrocyte and microglia morphology. Fig. S12. VBIT-4 increases CS and ATP synthase expression in astrocytes in 5 × FAD mice. Fig. S13. VBIT-4 increases CS and ATP synthase expression in microglia in 5 × FAD mice. Fig. S14. VBIT-4 reduces p-NF-kB —p65 and TNF-α expression in 5 × FAD mice. Fig. S15. VBIT-4 reduces IL1-β expression in 5×FAD mice. Fig. S16. VBIT-4 reduces NRLP3 and caspase-1 expression in 5 × FAD mice. Fig. S17. VBIT-4 reduces IBA-1 and caspase-1 expression in 5 × FAD mice. Fig. S18. VBIT-4 increases the expression of IL-4 and TGF-β in microglia of 5 × FAD mice. Fig. S19. VBIT-4 increases the expression of IL-4 TGF-β in astrocytes of 5 × FAD mice. [file 40035_2022_329_MOESM1_ESM.pdf]

Supplementary Materials

Targeting the Mitochondrial Protein VDAC1 Mitigates Brain Pathology in Models of Alzheimer's Disease

Ankit Verma[1, 2#], Anna Shteinfer-Kuzmine[2#], Nikita Kamenetsky[1,2], Srinivas Pittala[1,2], Avijit Paul[1,2], Edna Nahon Crystal[3], Alberto Ouro[4†], Vered Chalifa-Caspi[5], Swaroop Kumar Pandey[2], Alon Monsengo[6,] Noga Vardi[1§], Shira Knafo[2,4], and Varda Shoshan-Barmatz[1,2*]

*Corresponding author. Varda Shoshan-Barmatz, vardasb@bgu.ac.il

This PDF file includes:

Supplementary Text

Figs. S1 to S19

Tables S1 to S4

Materials and Methods

Materials

Bovine serum albumin (BSA), phenylmethylsulfonyl fluoride (PMSF), propidiumiodide (PI), Triton X-100, Tween-20, hematoxylin, and eosin were obtained from Sigma (St. Louis, MO). HEPES was from Holland Moran (Fisher Scientific, Geel, Belgium). The TUNEL assay kit was obtained from Promega (Cat. No. G3250; Madison, WI) and paraformaldehyde from Emsdiasum (Hatfield, PA). Dulbecco's Modified Eagle's Medium (DMEM) growth medium was obtained from Gibco (Grand Island, NY). Normal goat serum (NGS), fetal calf serum (FCS), and the supplements L-glutamine and penicillin-streptomycin and XTT (Cat. No. 20-300-1000) were obtained from Biological Industries (Beit Haemek, Israel). Ethylene glycol bis(succinimidyl succinate) (EGS) was obtained from Thermo Fisher (Cat. No. TS-21565; Waltham, MS, US). Primary antibodies, horseradish peroxidase (HRP)-conjugated, and fluorophore-conjugated secondary antibodies, their sources, and the dilutions used are detailed in Supplementary Table S1. 3,3-diaminobenzidine (DAB) was obtained from ImmPact-DAB (Cat. No. VE-SK-4105; Burlingame, CA).

VBIT-4 and VBIT-12 development

VBIT-4 and VBIT-12 were developed as described previously (19). Briefly, the interaction between VDAC1 monomers, was followed in live cells using the BRET2 (Bioluminescence Resonance Energy Transfer) technology. Genetically engineered rat rVDAC1 fusion proteins composed of either rVDAC1-RLuc or rVDAC1-GFP2 cloned into the BRET2 vectors were produced. BRET signals in the cells exposed to apoptosis induction (leading to VDAC1 oligomerization) and subjected to high-throughput screening of drug libraries to look for inhibitors of the BRET signal. Following the identification of the compounds inhibiting VDAC1 oligomerization and apoptosis at low concentration and have no effect on cell viability of control cells were selected. The lead molecules were subjected to several rounds of medicinal chemistry, leading to the development of VBIT-4 and VBIT-12 from two different lead compounds. An issued patent protects these compounds. Both compounds were synthesized by Cham-Partner Co., Ltd. (Shanghai, China).

Cell culture, cisplatin treatment, cell viability assay, and apoptosis

Human neuroblastoma-derived SH-SY5Y (CRL-2266) was from ATCC (Manassas, Virginia). SH-SY5Y cells were maintained using Dulbecco's modified Eagle's medium (DMEM), supplemented with 10% inactivated fetal bovine serum (FBS), and with 100 U/mL penicillin and 100 μg/mL streptomycin. Cells were grown at 37°C in 5% CO_2 in a humidified incubator. For cisplatin treatment, cells (60% confluence) were incubated for 24 h in a complete growth medium with different concentrations of cisplatin (5–20μM) in the absence or presence of VBIT-4 and analyzed for cell vaibility using the XTT assay, according to the manufacturer's instructions. Cell death was analyzed using propidium iodide (PI) staining subjected to flow cytometry analysis.

VDAC1 overexpression and oligomerization analysis

Cells were treated with the cisplatin as described above, harvested, washed with PBS at pH 8.3, and then were incubated at 1 mg of protein/ml with or without EGS (100 μM, 15 min). Lysates (20 µg

protein for measuring VDAC1 expression levels and 60–80 μg protein for visualizing VDAC1 oligomerization) were resolved by SDS–PAGE and electro-transferred to nitrocellulose membranes that were subsequently blocked with 5% non-fat dry milk and 0.1% Tween-20 in Tris-buffered saline (TBS). Samples were then incubated in primary antibodies (sources and dilutions listed in Table S1), and then with the appropriate secondary HRP-conjugated anti-mouse or anti-rabbit antibody (Table S1). Enhanced chemiluminescence substrate EZ-ECL (Cat. No. 205001000; Biological Industries, Israel) was used for detection of HRP activity. Analyses of immuno-reactive VDAC1 monomers, dimers, and multimer bands was performed using FUSION-FX (Vilber Lourmat, France).

TUNEL assay

Paraffin-embedded fixed brain sections were processed for a TUNEL assay using the DeadEnd Fluorometric TUNEL assay (Promega, Madison, WI), according to the manufacturer's instructions. Sections were deparaffinized, equilibrated in PBS, permeabilized with proteinase K (20 μg/ml in PBS), post-fixed in 4% paraformaldehyde, and incubated in TdT reaction mix for 1 h at 37°C in the dark. The slides were then washed in saline–sodium citrate buffer and counter-stained with propidium iodine (1μg/ml). After mounting with Vectashield mounting medium (Vector Laboratories, Burlingame, CA), images were collected using a confocal microscope (Olympus IX81).

Thioflavin-S Aβ staining

Aβ plaques in brains were visualized using thioflavin-S staining (Cat. No. T1892-25G; Merck, Kenilworth, New Jersey, US). Brain sections were incubated as follows: 100% xylene (5 min twice), 50%/50% xylene:100% ethanol (3 min) and then through a series of ethanol solutions (100%, 95%, 80%, 70%, 1–2 min in each). They were then incubated for a few seconds in water and stained with 1% Thioflavin-S for 30 min. Sections were washed in ethanol solutions (70%, 80%, 95%, 100%, 100%, 1–2 min in each) and then coverslipped with mounting media. Sections were viewed under a fluorescent microscope.

RNA isolation from fixed samples and quantitative real-time PCR (RT-PCR) analysis

Total RNA was isolated from 20-μm sections cut from formaldehyde-fixed brains using the Recover All™ Total Nucleic Acid Isolation Kit for FFPE (Cat. No. AB-AM1975; Ambion™, Austin, Texas, US), according to the manufacturer's protocol. Total RNA quality was analyzed using the Agilent RNA 6000 nano kit. The RNA integrity values obtained for total RNA extracted were 8–10. Complementary DNA (cDNA) was synthesized using a qPCRBIO cDNA synthesis kit (Cat. No. PB30.11-10; PCR Biosystems, London, UK,) according to the manufacturer's protocol.

Quantitative-PCR was performed with specific primers (Table S2) in triplicates using SYBR Green master mix (Cat. No. 4367659; Thermo Fisher, Waltham, MS, US). Next, samples were amplified by the 7300 Real-Time PCR System (Applied Biosystems). Following this, target gene levels were normalized with α-actin mRNA as an internal control, and the mean fold change (± SEM) of the three replicates of 3–4 mice per group were calculated.

Table S1. Antibodies used in this study

Antibodies against the indicated protein, their catalogue number, source, and the dilutions used in IHC, immunoblot, and immunofluorescence experiments are presented.

No	Antibody	Source and Cat. No.	IHC	IF	WB
**1.**	Mouse monoclonal anti-VDAC1	Abcam, Cambridge, UK, ab186321	-	1:500	
**2.**	Rabbit polyclonal anti-VDAC1	Abcam, Cambridge, UK, ab15895	-	1:500	1:5000
**3.**	Rabbit anti-Amyloid β	Provided by Dr. Monsonego (109)	1:400	1:400	
**4.**	Mouse monoclonal anti-Amyloid β	Biolegend, San Diego, USA, 803001	-	1:300	
**5.**	Rabbit monoclonal anti-Tau (phospho S396)	Abcam, Cambridge, UK, ab32057	-	1:400	
**6.**	Chicken polyclonal anti-Prion protein PrP	Abcam, Cambridge, UK, ab178545	-	1:500	
**7.**	Mouse monoclonal anti-NeuN	Merck, U.S.A., MAB377	-	1:500	
**8.**	Rabbit monoclonal anti-synaptophysin	Abcam, Cambridge, UK, ab32127	-	1:400	
**9.**	Rabbit polyclonal anti-p-NFκB-p56 (Ser536)	Bioss Antibodies, Massachusetts, USA, bs-0982R	-	1:400	
**10.**	Rabbit polyclonal anti-NLRP3/NALP3	Novus Biologicals, CO, U.S.A. NB77080SS	-	1:300	
**11.**	Rat monoclonal anti-caspase-1	Biolegend, San Diego, USA, 645101	-	1:300	
**12.**	Rabbit polyclonal anti-IL-1β	Novus Biologicals,CO, U.S.A. NB600633	-	1:200	
**13.**	Rabbit polyclonal anti-cleaved caspase-3	Cell Signaling Technology, MA, USA, 9661S		1:300	
**14.**	Mouse monoclonal anti-TUBB3	Biolegend, San Diego, USA, 801202	-	1:500	
**15.**	Rabbit polyclonal anti-PSD-95	Abcam, Cambridge, UK, ab18258	-	1:300	
**16.**	Rabbit polyclonal anti-P53	Abcam, Cambridge, UK, ab131442	-	1:200	1:2000
**17.**	Mouse monoclonal anti-TNFα	Abcam, Cambridge, UK, ab1793		1:200	
**18.**	Mouse monoclonal anti-glucose transporter Glut-2	Santa Cruz Biotechnology, Texas USA, sc-518022		1:300	
**19.**	Mouse monoclonal anti-GFAP	Santa Cruz Biotechnology, Texas USA, sc-33673		1:500	
**20.**	Rabbit monoclonal anti-glucose transporter Glut-4	Abcam, Cambridge, UK, ab33780		1:400	
**21.**	Rabbit monoclonal anti-hexokinase I	Abcam, Cambridge, UK, ab150423	-	1:200	
**22.**	Rabbit monoclonal recombinant anti-Na,K-ATPase	Abcam, Cambridge, UK, ab76020	-	1:200	
**23.**	Rabbit polyclonal anti-ATP Synthase 5A	Abcam, Cambridge, UK, ab151229	-	1:300	
**24.**	Rabbit polyclonal anti-citrate synthetase	Abcam, Cambridge, UK, ab96600	-	1:300	
**25.**	Rabbit monoclonal anti-Amylin	Abcam, Cambridge, UK, ab254259	-	1:400	
**26.**	Rabbit monoclonal anti-TSPO	Abcam, Cambridge, UK, ab109497		1:1000	
**27.**	Mouse monoclonal anti-IBA	Abcam, Cambridge, UK, ab283319		1:250	
**28.**	Rabbit monoclonal anti-glucose transporter Glut-1	Abcam, Cambridge, UK,ab115730		1:1000	
**29.**	Mouse monoclonal anti-Glutamine synthetase	Bio-Legend, California, US BLG-856202		1:500	
**30.**	Rabbit monoclonal anti-Cytochrome c oxidase subunit Vic	Abcam, Cambridge, UK, ab150422		1:250	
**31.**	Mouse monoclonal anti-IL4	Santa Cruz Biotechnology, Texas, USA, sc-53084		1:500	
**32.**	Mouse monoclonal anti-TGFβ1	Santa Cruz Biotechnology, Texas, USA sc-130348		1:500	
**33.**	Goat anti-chicken, alexa fluor 555	Abcam, Cambridge, UK, ab150170		1:1000	
**34.**	Anti-mouse IgG, Alexa Fluor 488	Abcam, Cambridge, UK, ab150109	-	1:1000	

**35.**	Anti-mouse IgG, Alexa Fluor 555	Abcam, Cambridge, UK, ab 150110	-	1:1000	
**36.**	Anti-rabbit IgG, Alexa Fluor 555	Abcam, Cambridge, UK, ab150086	-	1:1000	
**37.**	Anti-rabbit IgG, Alexa Fluor 488	Thermo Fisher Scientific, USA, A-110	-	1:1000	
**38.**	Anti-rat IgG, Alexa Fluor 488	Abcam, Cambridge, UK, ab150153	-	1:1000	
**39.**	Anti-mouse IgG, Alexa Fluor 568	Thermo Fisher Scientific, USA, A-110	-	1:1000	
**40.**	Anti-goat polyclonal chicken, Alexa Fluor 555	Abcam, Cambridge, UK, ab150170	-	1:1000	

Table S2. Real-time PCR primers used in this study

The genes examined and the forward and reverse sequences of the primers used are indicated.

Gene	Primer sequences
β-Actin	Forward: 5'- CATTGCTGACAGGATGCAGAAGG -3'<br>Reverse: 5'- TGCTGGAAGGTGGACAGTGAGG -3'
Synaptophysin	Forward: 5'- TTGGCTTCGTGAAGGTGCTGCA -3'<br>Reverse: 5'- ACTCTCCGTCTTGTTGGCACAC -3'
NF-kB-p65	Forward: 5'- GCATGCGATTCCGCTATAAATG -3'<br>Reverse: 5' - GTCCTGTGTAGCCATTGATCTT -3'
Caspase 1	Forward: 5'- TGGTATCCAGGAGGGAATATGT -3'<br>Reverse: 5'- TCACCTTGGGCTTGTCTTTC -3'
IL-1β	Forward: 5'- TGGACCTTCCAGGATGAGGACA -3'<br>Reverse: 5'- GTTCATCTCGGAGCCTGTAGTG -3'
GLUT1	Forward: 5'- GCTTCTCCAACTGGACCTCAAAC-3'<br>Reverse: 5'- ACGAGGAGCACCGTGAAGATGA-3'
IL-4	Forward: 5'- ATCATCGGCATTTTGAACGAGGTC -3'<br>Reverse: 5'- ACCTTGGAAGCCCTACAGACGA -3'

Supplementary Results

Table S3. VDAC1 promoter sites matching sequence profiles generated from AβID decamers identified by Maloney and Lahiri

Profiles for the Aβ–Interacting Domain (AβID) were generated from two sets of sequences: (A) six EMSA-validated AβID decamers from APOE, APP, BACE1, and TP53, presented in Table 2 in (110); and (B) 35 computationally-predicted AβID decamers from the promoters of 10 different genes, presented in Table 3 in (110). The profiles were constructed using MEME (111), while requesting that for each sequence set, only one profile (a.k.a. "motif") of 10 bp would be generated. The profiles were then searched with FIMO (111) against a 997-bp region surrounding the VDAC1 mRNA (RefSeq NM_003374.3) transcription start site (TSS, chr5:134,004,568-134,005,564 on the Human GRCh38/hg38 Assembly), consisting of 589 bp upstream and 408 bp downstream of the TSS. The results show profile occurrences with a *p*-value less than 0.0001. The *p*-value of a profile occurrence is defined as the probability of a random sequence of the same length as the profile matching that position of the sequence with as good or a better score. The score for the match of a position in a sequence to a profile is computed by summing the appropriate entries from each column of the position-dependent scoring matrix that represents the profile. The *q*-value of a profile occurrence is defined as the false discovery rate if the occurrence is accepted as significant.

		-	Genomic location				Statistical results		
No.	Profile	Matched sequence VDAC1 promoter	Start	End	Distance from TSS	Strand	Score	*p*-value	*q*-value
1	A<br>B	GGGGATGGGG	281	290	-309	+	15.68<br>14.29	5.01E-06<br>5.87E-06	0.00699<br>0.00805
2	B	GGGGGAGGAG	477	486	-113	-	12.54	2.45E-05	0.0168
3	B	GGGGGCGGAG	739	748	149	+	10.96	7.71E-05	0.0232
4	A	GGGCCTGGGG	733	742	143	+	8.89	8.02E-05	0.0424
5	B	GGGAAGGGGG	482	491	-108	-	10.86	8.15E-05	0.0232
6	B	GGGGCCGGGG	310	319	-280	+	10.77	8.45E-05	0.0232
7	A	CGGGATGGGG	691	700	101	+	8.61	9.12E-05	0.0424

The most significant site on the VDAC1 promoter was located 309 bp upstream of the TSS. This site contained the four 3’ Gs which appeared in all EMSA-validated sequences, including the G marked with an asterisk in Fig. 4 in (2), whose alteration to A was shown to significantly diminish Aβ binding (2). Moreover, this site was nearly identical (9 out of 10 bases) to the decamer predicted by dynamic weight analysis (2) to have the maximum possible score (GGGGTTGGGG) as AβID.

Table S4. ADME/PK Profile – Summary

These studies were carried out by Shanghai ChemPartner Co., Ltd

Parameter	Value
Caco2 permeability	6.8x10-6 cm/sec (A-B)<br>7.8x10-6 cm/sec (B-A)
Solubility in SIF<br>(Simulated intestinal fluid)	72 μM
Metabolic Stability	$T_{1/2}$ in HLM ~55min; RLM ~75min
Partitioning to RBC	KRBC/PL = 0.36 (human)<br>KRBC/PL = 0.7 (rat)
Plasma Protein Binding	Bound Fraction:<br>99.9% (human); 99.7% (rat)
CYPs inhibition	IC50 > than 10 μM,
PK Profile	F%=65; T1/2= 7.6h<br>Cmax=3310ng/ml, Tmax=1.33hr
Single dose toxicity	No toxicity
Mini AMES	No mutagenic potential

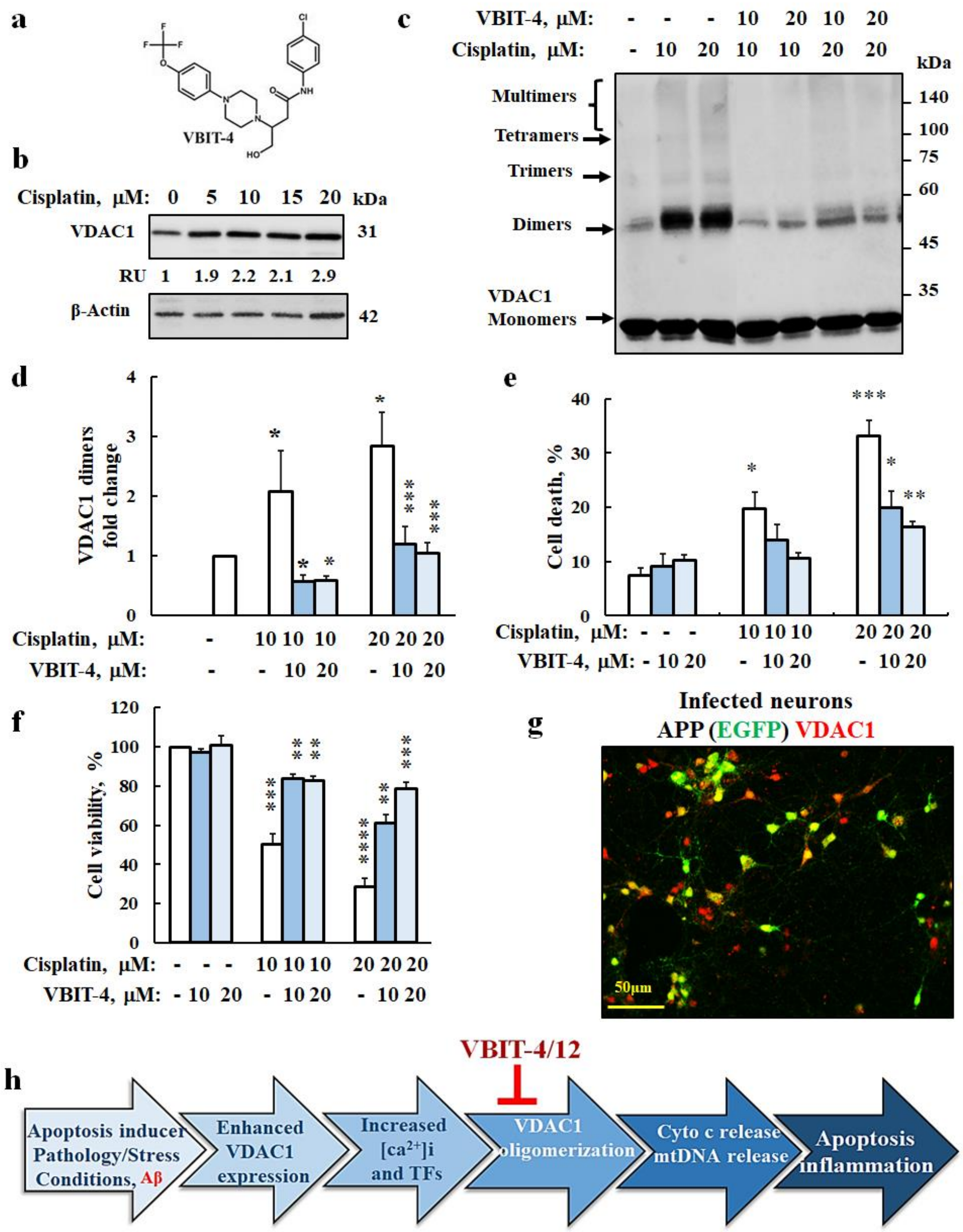

Fig. S1. Aβ induces VDAC1 overexpression, oligomerization, and apoptotic cell death. (a) VBIT-4 structural formula. (**b**) SH-SY5Y cells were incubated (24 h) with cisplatin and analyzed for VDAC1 expression levels, shown in relative units (RUs). (**c-e**) Cells were incubated with cisplatin in the absence and presence of the indicated concentrations of VBIT-4, then VDAC1 oligomerization, as revealed using EGS-based cross-linking and quantitative analysis of VDAC1 dimers (**c,d**), and apoptotic cell death was assayed using propidine iodin and FACS analysis (**e**), as previously described (19). The positions of VDAC1 monomers and oligomers and of the molecular weight standards are indicated. The cells were also subjected to a survival assay using XTT (**f**). (**g**) Primary neural culture was infected with APP swe/lnd-EGFP Sindbis virus for 16 h to overexpress and secret Aβ into the medium (conditioned medium). Immunostaining of APP-infected cells for VDAC1 and the expressed GFP is seen. (**h**) Apoptosis stimuli, pathological or stress conditions, and Ab enhance VDAC1 expression via activating the VDAC1 promoter by transcription factors (TFs) and/or Ca^{2+}, leading to VDAC1 overexpression, and oligomerization of the overexpressed VDAC1, allowing cytochrome c or/and mtDNA release, and thereby apoptosis and inflammation.

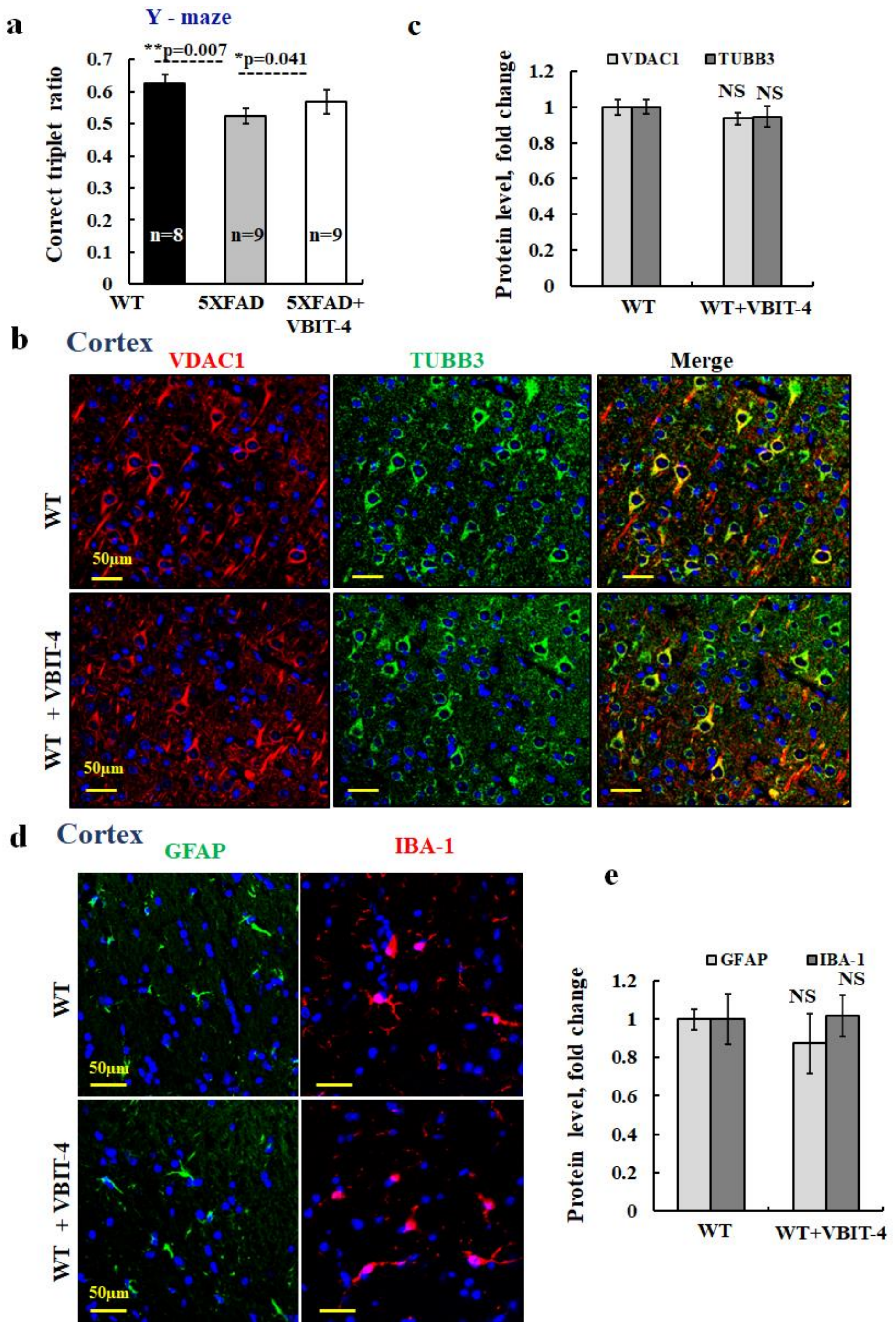

Fig. S2. VBIT-4 improves cognition, learning, and memory performance in the 5XFAD mouse model and had no effect on WT mice. (a) Y-Maze test was performed on WT (n=8), and untreated and 5-month-treated 5XFAD mice (n=9) with VBIT-4 (n=9). Performance in the Y-maze allowing mice to explore all three arms of the maze. An ANOVA yielded a significant difference among the groups [f(2,33)=4.46, *p*=0.019]. Tukey post hoc analysis revealed that 5XFAD mice performed more poorly than WT mice (*p*=0.007), and VBIT-4-treated 5XFAD mice performed better than untreated mice (*p*=0.041). (**b-e**) Confocal images of cortical and hippocampal sections from WT, untreated, and VBIT-4-treated mice co-immunostained for VDAC1 and TUBB3 (**B**), or GFAP or IBA-1 (**d**) and their quantification (**c,e**). Results show means ± SEM (n=3), NS, not significant, showing no effect of VBIT-4 on the expression of the tested proteins in WT mice.

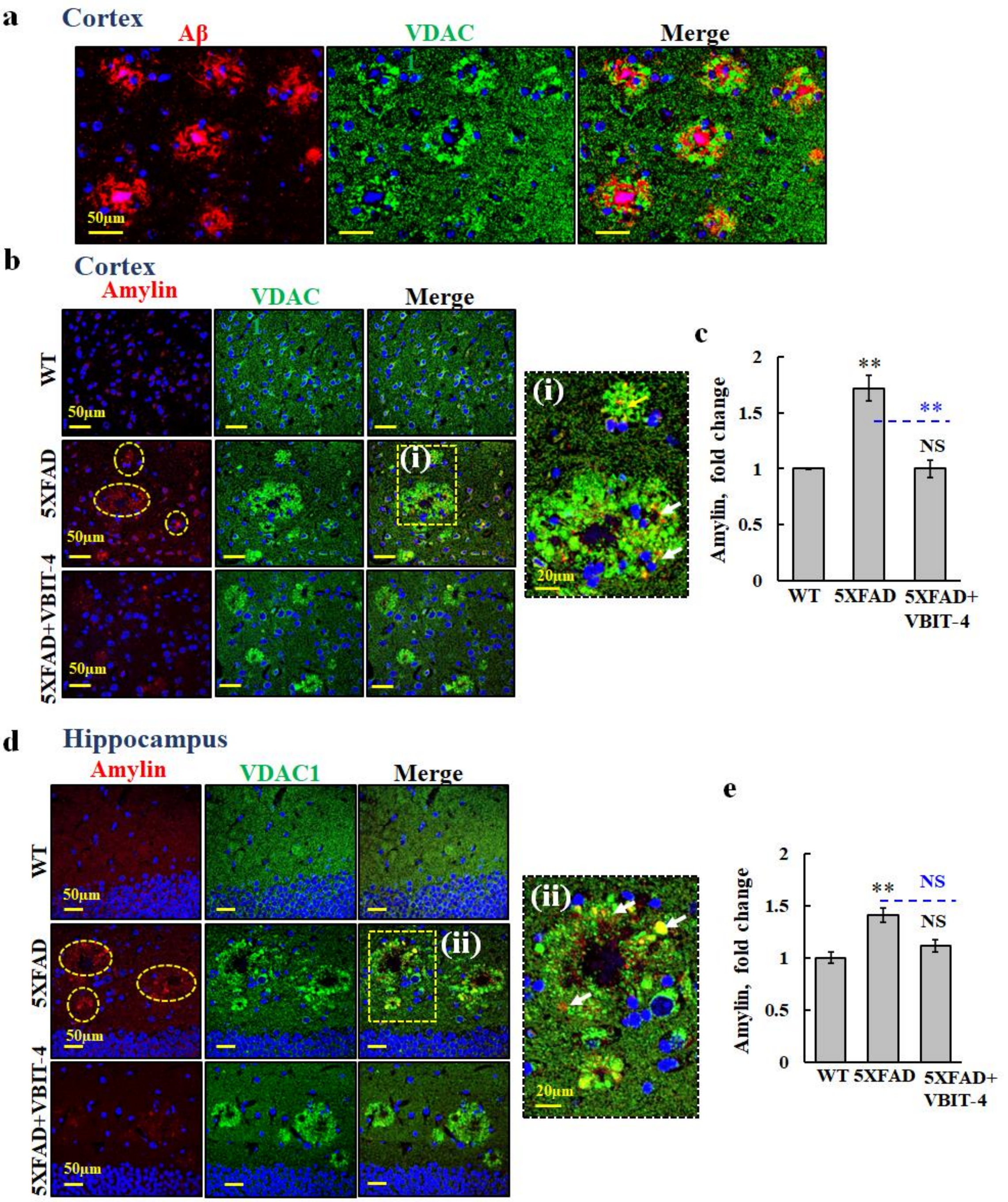

Fig. S3. Effect of VBIT-4 treatment on the levels of amylin expression in the 5XFAD brain. (a) IF co-staining for Aβ and VDAC1 in cortical sections, showing overexpressed VDAC1 in the neuropils surrounding the plaques. (**b–e**) IF co-staining for amylin and VDAC1 in cortical and hippocampal sections; staining in the Aβ plaques is circled, and selected regions in the merged images are enlarged **(i)** and **(ii)**. White arrows point to co-localization of VDAC1 and amylin. (**c,e**) Quantification of amylin expression levels in the cortex and hippocampus. Results are shown as means ± SEM (n=3), ***p* <0.01. *p*-value in blue represents the significance of VBIT-4 treated mice relative to untreated mice. NS, not significant (n=3).

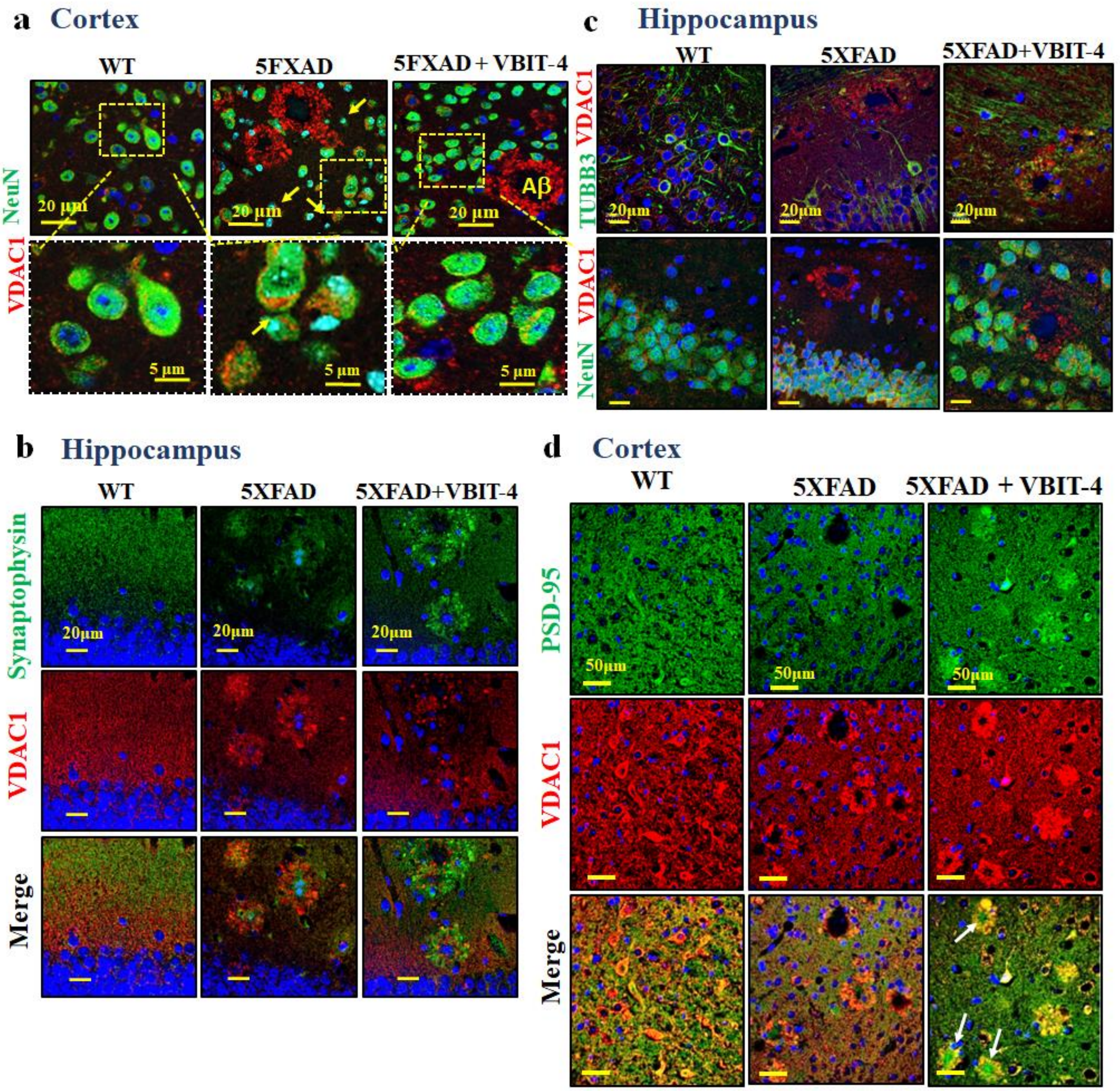

Fig. S4. VBIT-4 treatment of 5XFAD mice prevents neuronal and synaptic loss. (a) Co-immunostaining for VDAC1 and NeuN of cortical sections from WT, untreated- and VBIT-4-5XFAD mice. (**b,c**) Hippocampal sections from WT, and untreated and VBIT-4-treated 5XFAD mice co-immunostained for VDAC1 and either synaptophysin (B), TUBB3, or NeuN (C). (**d**) Cortical sections from WT, untreated, and VBIT-4-treated-5XFAD mice co-immunostained for VDAC1 and PSD-95. White arrows point to PSD-95 staining in the Aβ plaques that are not co-localized with VDAC1.

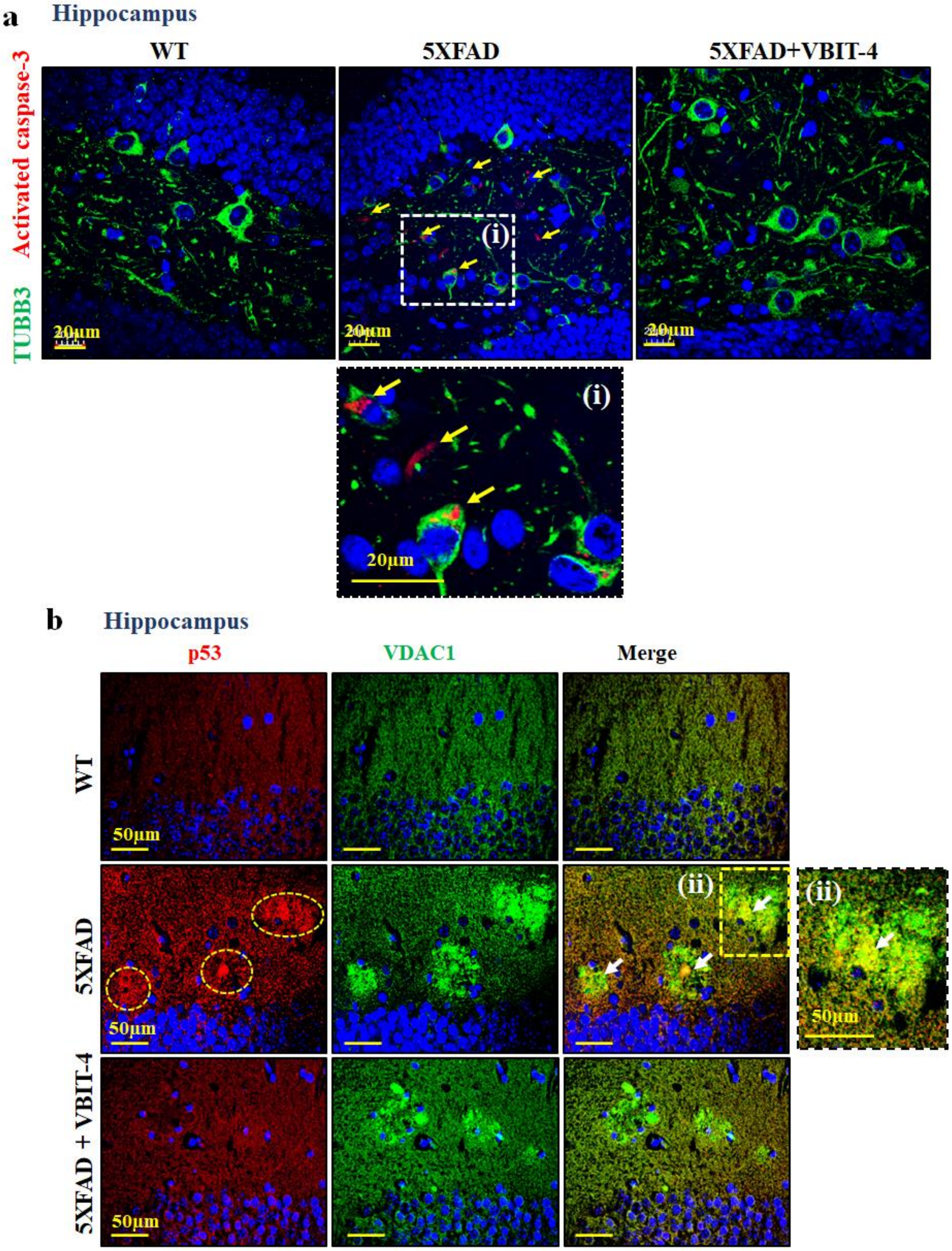

Fig. S5. VBIT-4 treatment of 5XFAD mice protects against cell death. (a) Confocal images of hippocampal sections from WT, and untreated and VBIT-4-treated 5XFAD mice co-immunostained for TUBB3 and activated caspase-3 (arrows). (**b**) Confocal images of hippocampal sections co-immunostained for p53 and VDAC1, showing increased expression of p53 in the Aβ plaques (circled) and co-localization with VDAC1 (arrows). Higher magnification of the selected area is shown in (i) and (ii).

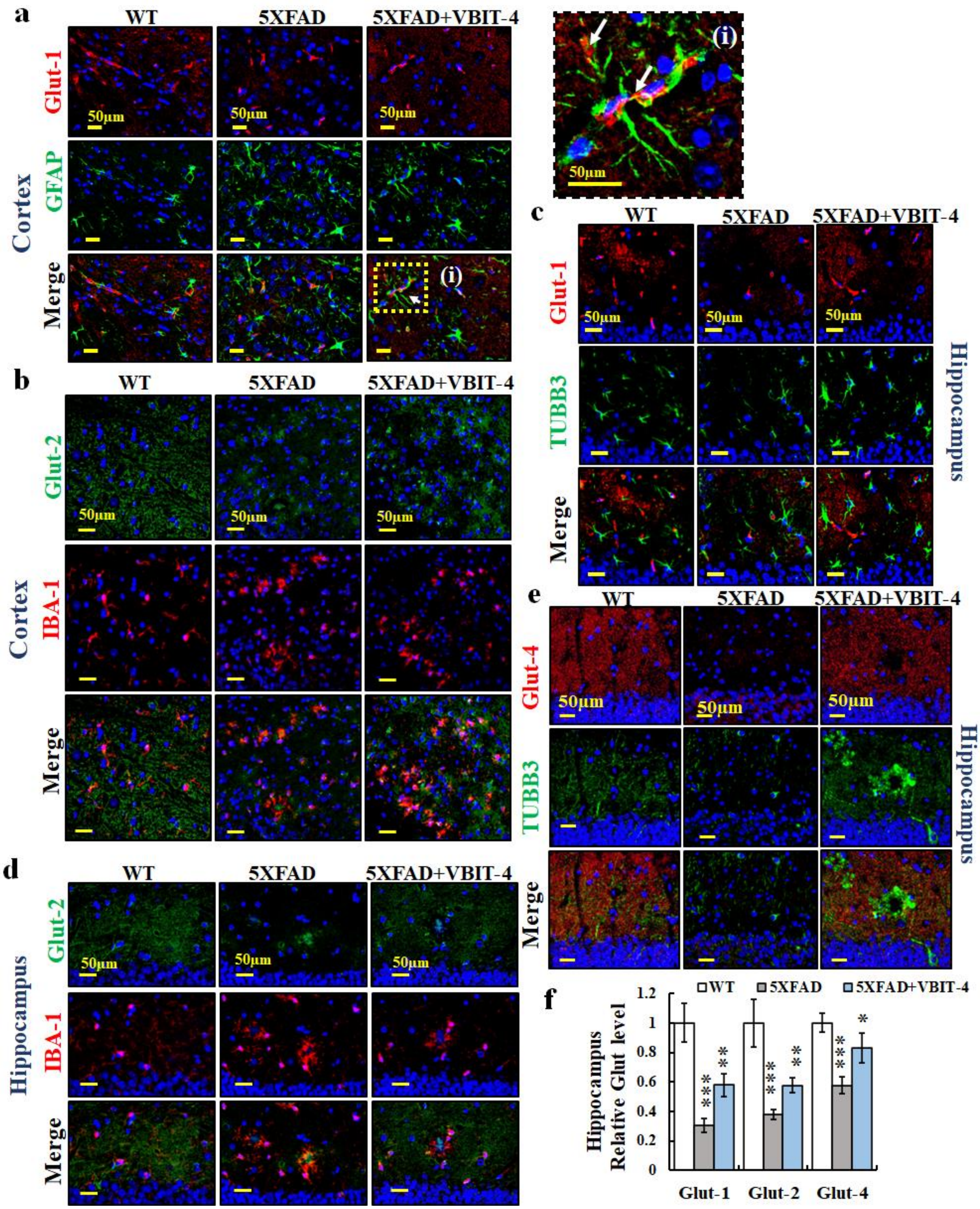

Fig. S6. VBIT-4-treatment attenuated a decrease in the expression of glucose transporters in 5XFAD mice. (**a,b**) Confocal images of cortical sections from WT, and untreated and VBIT-4-treated 5XFAD mice co-immunostained for Glut-1 and GFAP (**a**), and Glut-2 and IBA-1 (**b**). White arrows in the enlarged image (**a(i)**) point to Glut-1 localized in astrocytes' dendritic end-feet attaching the blood vessels. (**c–e**) Co-immunostaining of hippocampal sections for Glut-1 and TUBB3 (**c**), Glut-2 and IBA-1 (**d**), and Glut-4 and TUBB3 (**e**). (**f**) Quantitative analysis of glucose transporter expression levels. Results show means ± SEM (n=3), ***p* <0.01 ****p* <0.001.

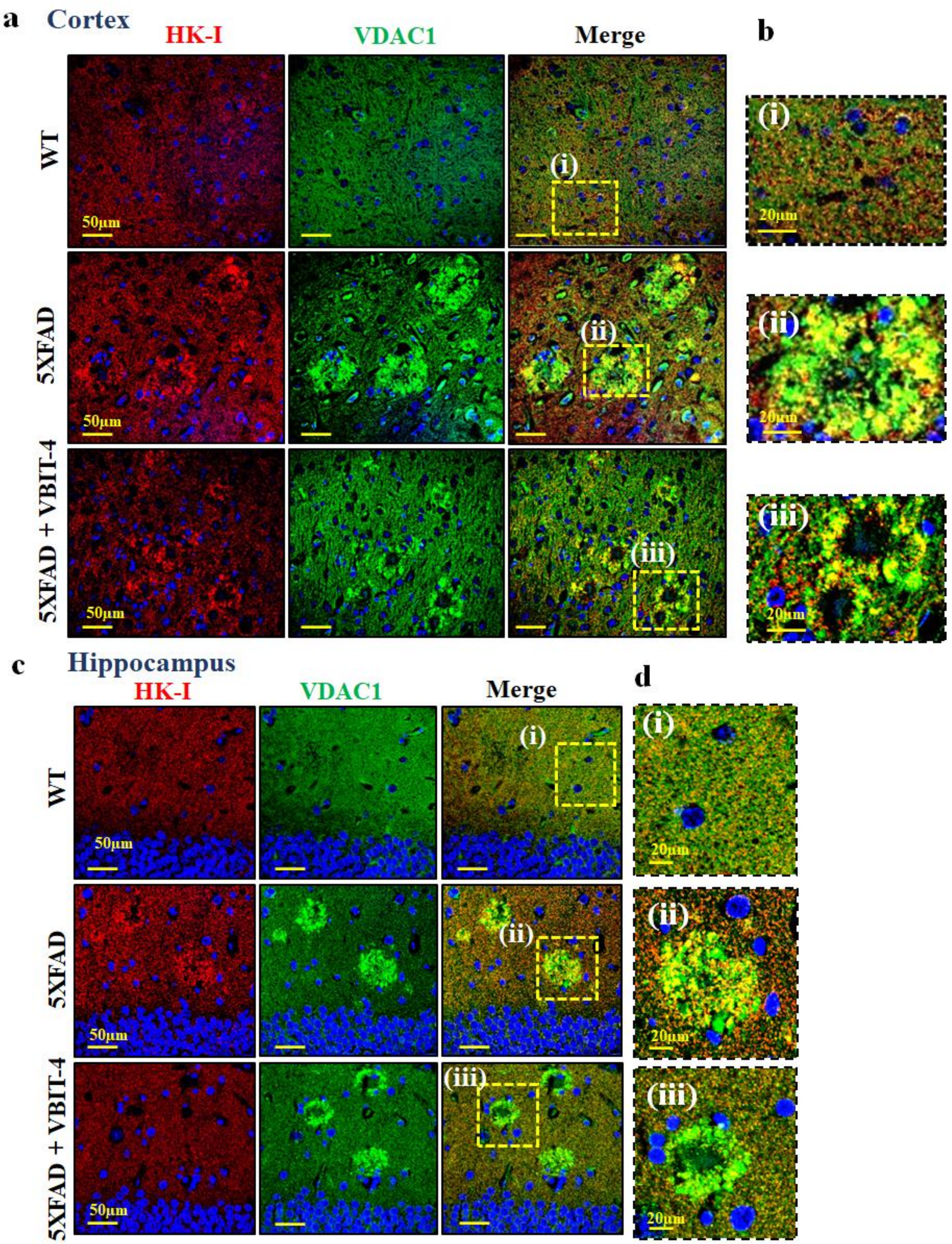

Fig. S7. HK-I is overexpressed and co-localized with VDAC1 in the neuropils surrounding the Aβ plaques. Cortical (**a,b**) and hippocampal (**c,d**) sections from WT, and untreated and VBIT-4-treated 5XFAD mice co-immunostained for HK-I and VDAC1. Co-localization of HK-I with VDAC1 in the neuropils around Aβ plaques is further shown in the enlarged images (**b(i-iii), d(i-iii)**).

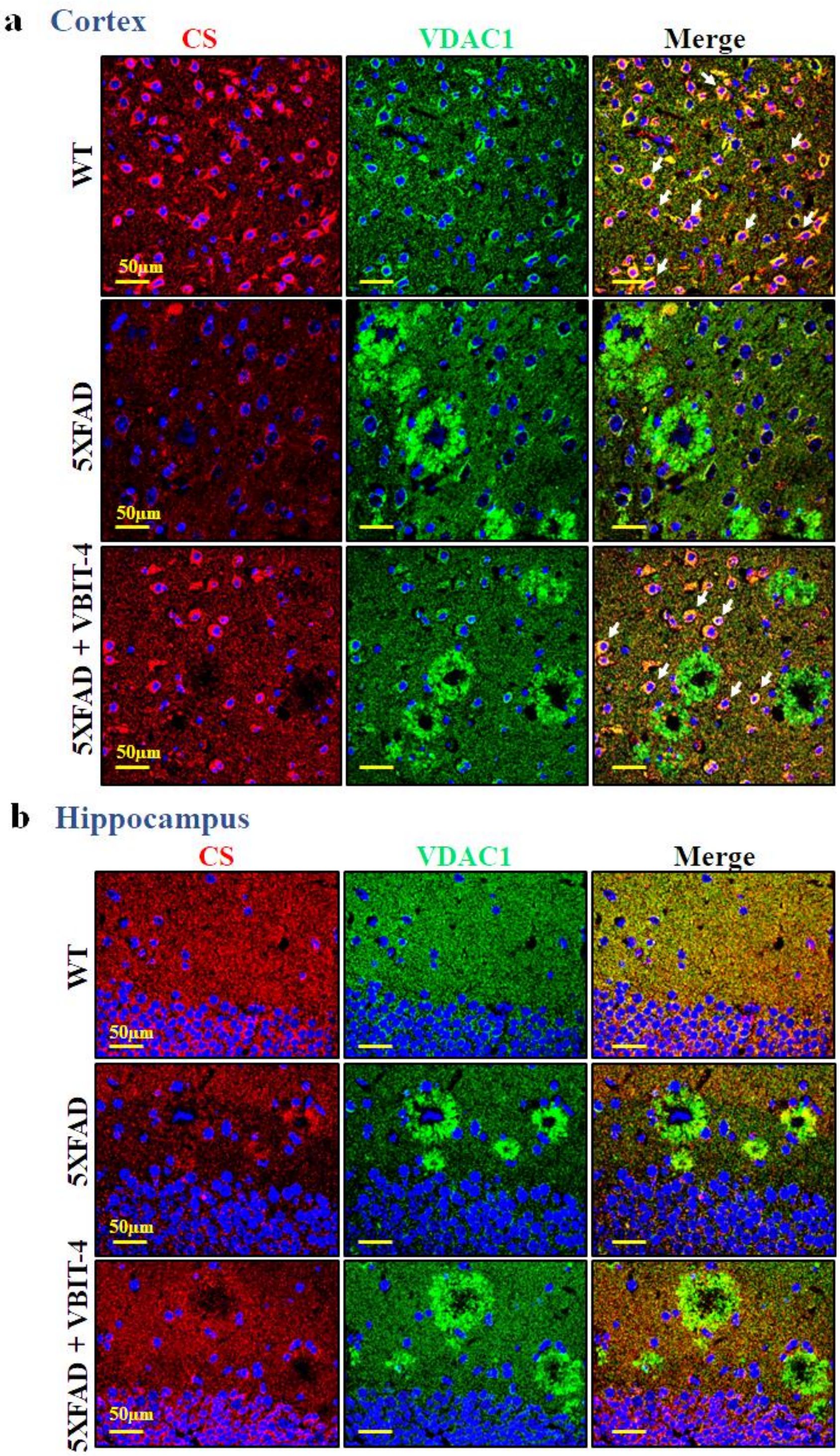

Fig. S8. VBIT-4 treatment of 5XFAD mice prevented the decrease in citrate synthase expression. Cortical (**a**) and hippocampal (**b**) sections co-immunostained for citrate synthase (CS) and VDAC1. In WT, CS was strongly expressed in the cell cytosol (arrows in A). This expression was dramatically decreased in untreated, and largely restored in the VBIT-4-treated 5XFAD mice. CS was not localized with VDAC1 in the neuropils surrounding the Aβ plaques.

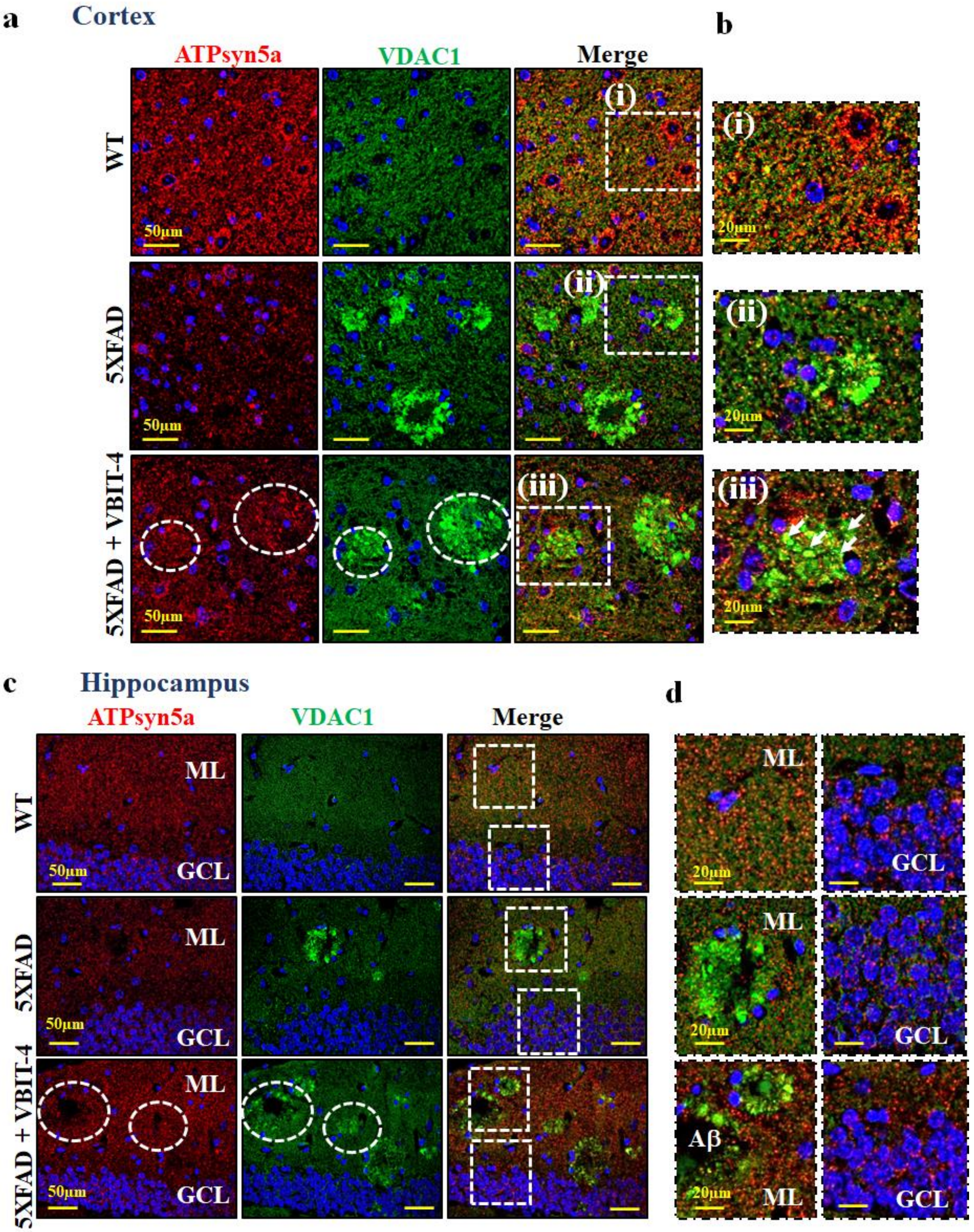

Fig. S9. VBIT-4 treatment of 5XFAD mice prevented a decrease in ATP synthase expression. Cortical (**a,b**) and hippocampal (**c,d**) sections co-immunostained for ATPsyn5a and VDAC1. In VBIT-4-treated 5XFAD mice, ATPsyn5a is expressed in the neuropil surrounding the Aβ plaques (circled). (**b**) ATPsyn5a was co-localized with VDAC1 in WT (**b(i)**) and in 5XFAD treated with VBIT-4 (arrows in **b(iii)**), but not in 5XFAD mice (arrows in **b(ii)**). (**d**) Magnification of the molecular layer (ML) and granule cell layer (GCL), showing ATPsyn5a expression levels in VBIT-4-treated 5XFAD mice.

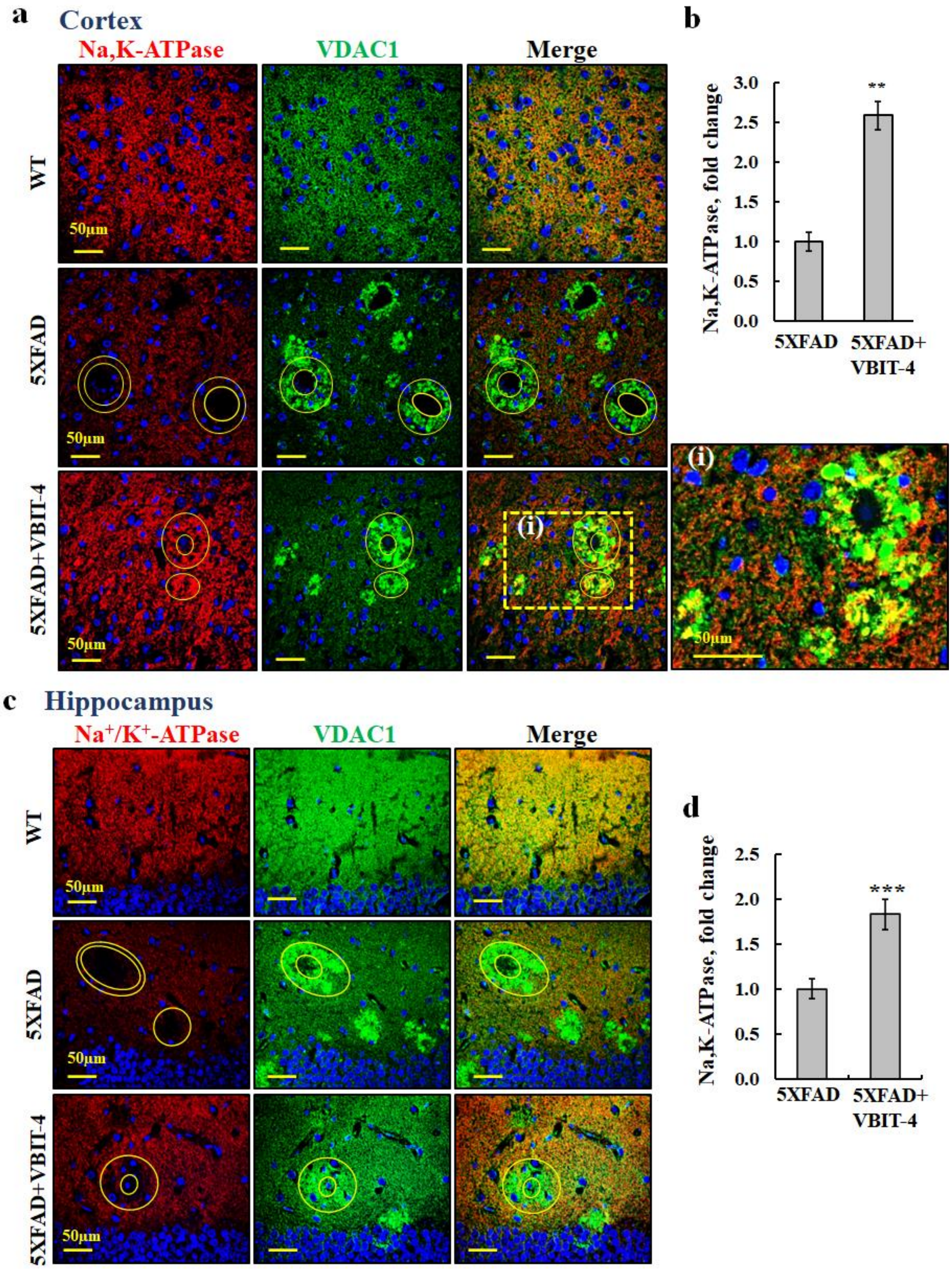

Fig. S10. VBIT-4 treatment of 5XFAD mice prevented a reduction in NaK-ATPase expression. Cortical (**a,b**) and hippocampal (**c,d**) sections co-immunostained for Na,K-ATPase and VDAC1. No expression of Na,K-ATPase was observed in the Aβ plaques surrounding neuropils in the 5XFAD mice, but Na,K-ATPase was expressed and co-localized with VDAC1 in the Aβ plaques surrounding the neuropils (circled) in VBIT-4-treated 5XFAD mice, is further shown in the enlarged image (**a(i)**). Quantitative analysis of total Na,K-ATPase expression levels in the cortical neuropils surrounding the Aβ plaques of VBIT-4-treated and untreated 5XFAD mice (**b,d**). Results show means ± SEM (n=3), ***p* <0.01 ****p* <0.001.

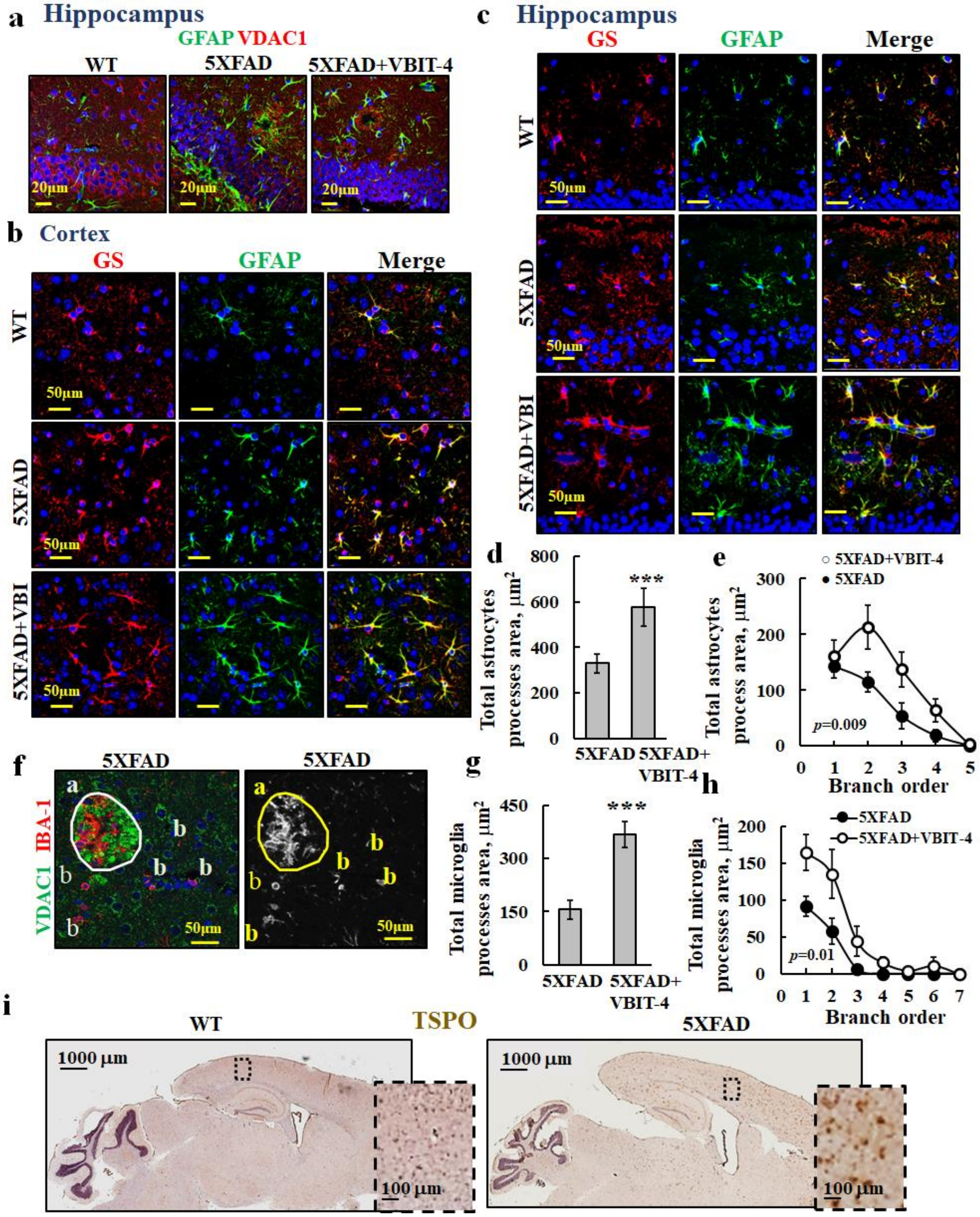

Fig. S11. VBIT-4 treatment of 5XFAD mice improves astrocyte and microglia morphology. (a) Hippocampal sections from WT, and untreated and VBIT-4-treated 5XFAD mice co-immunostained for VDAC1 and GFAP. (**b,c**) Cortical and hippocampal sections co-immunostained for glutamine synthase (GS) and GFAP. (**d,e**) Analysis of the 3D-reconstructed images of astrocytes in 50μm cortical sections from untreated and VBIT-4-treated 5XFAD mice, immunostained for GFAP, imaged using Spinning disk microscopy, and analyzed using Imaris software. Total area of all processes (**d**) and total process area as a function of branch order (**e**) are larger in astrocytes of treated mice. (**f**) Illustration of IBA-1 staining and its quantification in the Aβ plaques (**a**) and outside of the plaques (**b**) of black and white converted IBA1-staning. (**g,h**) Analysis for total process areas for microglia (as described above for astrocytes). Results show means ± SEM (n=3), *p*-values are presented, ****p* <0.001. (**i**) IHC staining of brain sections from WT and 5XFAD mice immunostained for TSPO, showing its increased expression in the form of plaques in the 5XFAD mice.

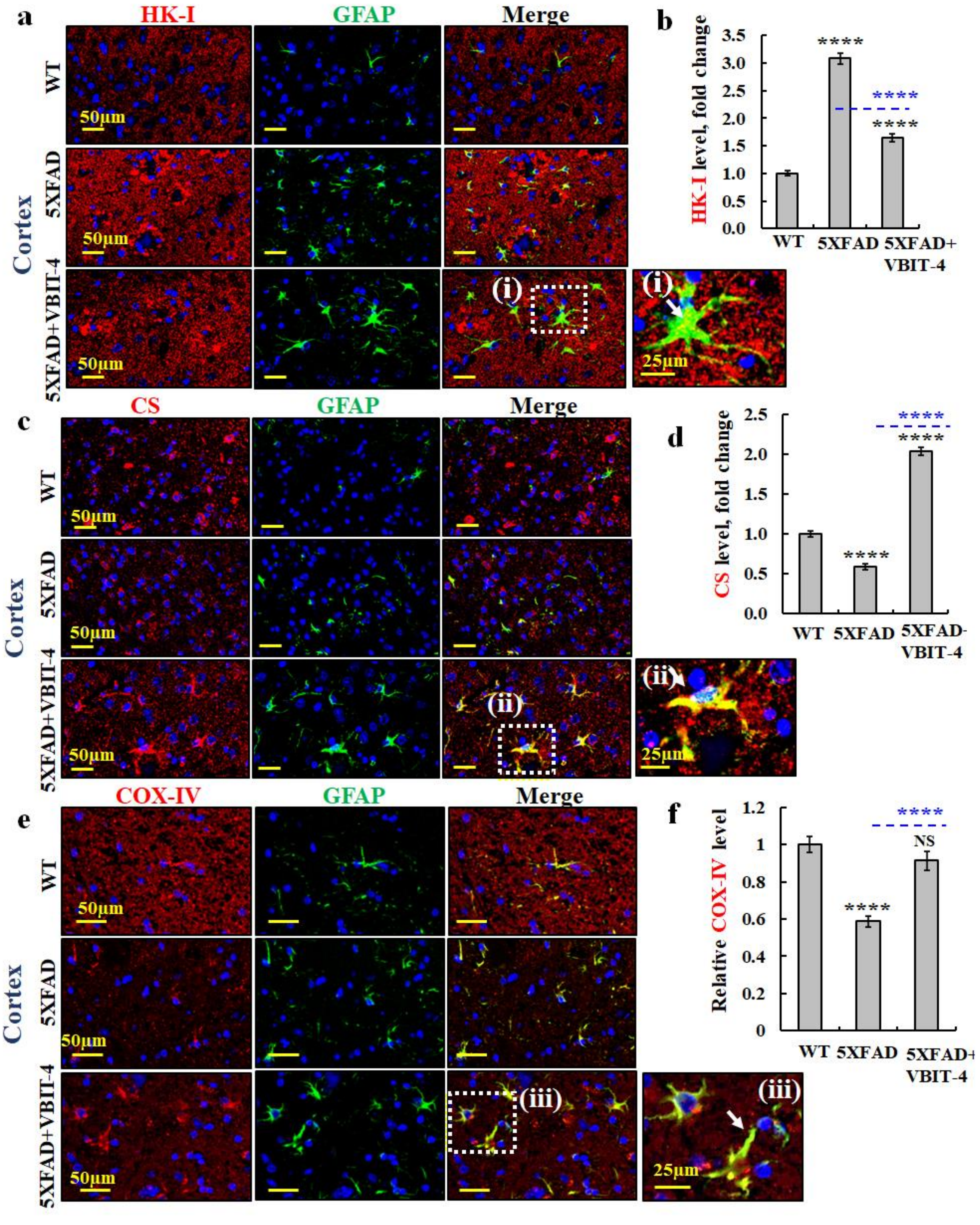

Fig. S12. VBIT-4 increases CS and ATP synthase expression in astrocytes in 5XFAD mice. Cortical sections from WT, and untreated and VBIT-4-treated 5XFAD mice were co-immunostained for GFAP and HK-I (**a,b**), or CS (**c,d**), or COX-IV (**e,f**). The morphology improved astrocytes in VBIT-4-treated 5XFAD mice, showing increased expression of the mitochondrial enzymes and their co-localization with GFAP, as is further shown in the enlarged images (**a(i), c(ii), e(iii)**). Results show means ± SEM (n=3). Results show means±SEM (n=3), ****p <0.0001. p-value in blue color represents the significance of VBIT-4-treated relative to untreated mice. NS, not significant.

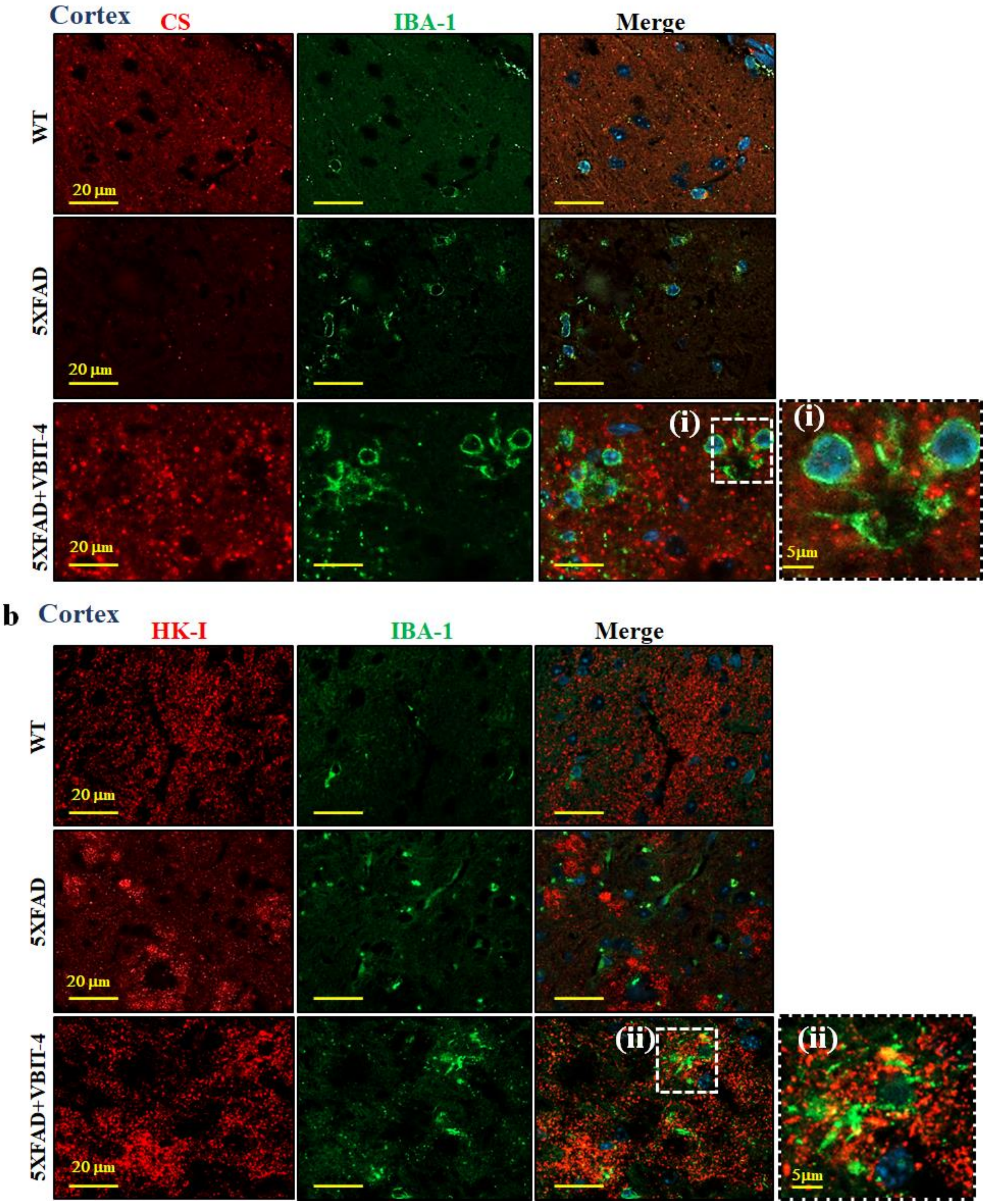

Fig. S13. VBIT-4 increases CS and ATP synthase expression in microglia in 5XFAD mice. Cortical sections from WT, and untreated and VBIT-4-treated 5XFAD mice were co-immunostained for CS and IBA1 (**a**), or HK-I and IBA1 (**b**). The morphology improved microglia in VBIT-4-treated 5XFAD mice, showing increased expression of the mitochondrial enzymes and their co-localization with IBA-1 as is further shown in the enlarged images (**a(i), b(ii)**).

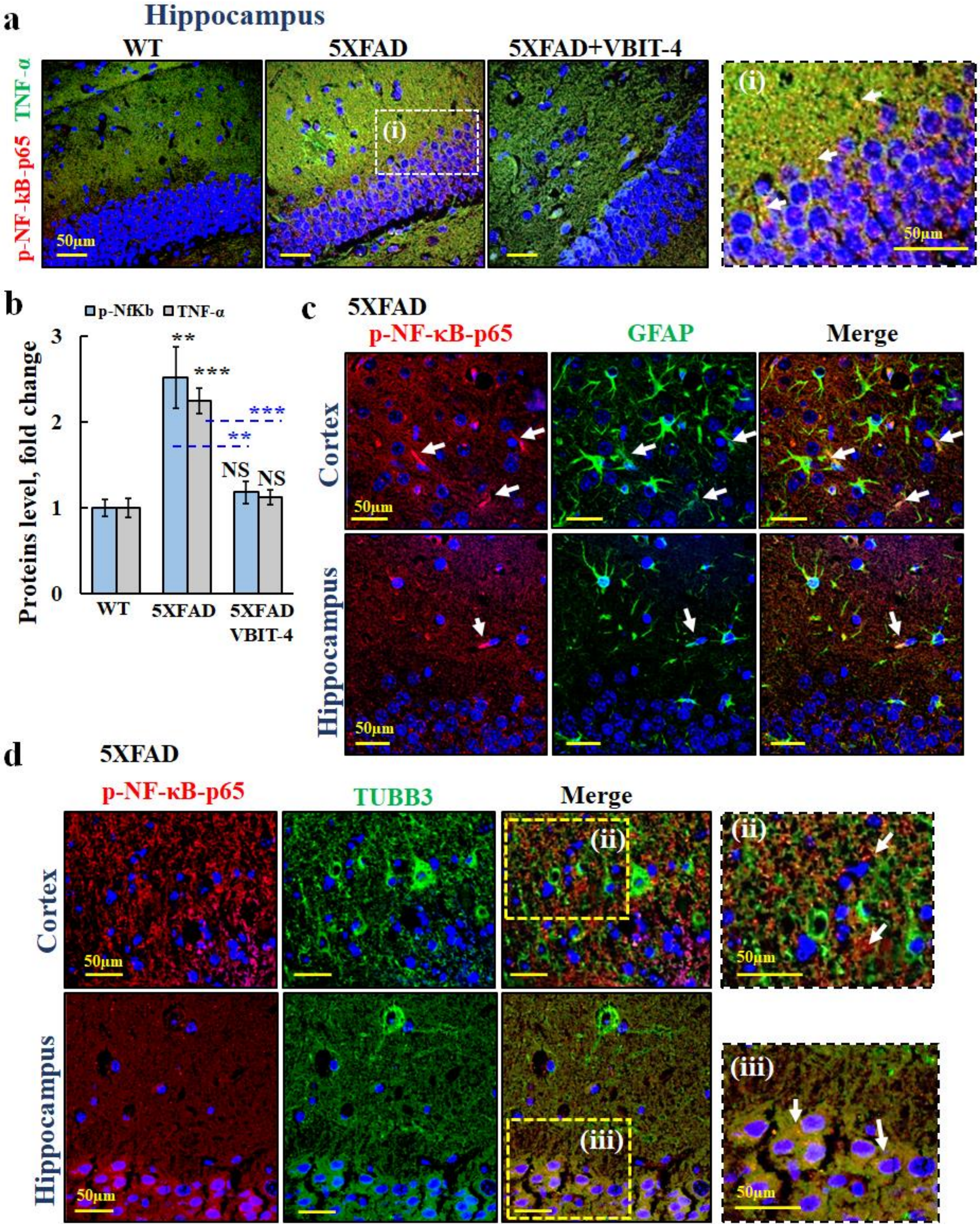

Fig. S14. VBIT-4 reduces p-NF-kB—p65 and TNF-α expression in 5XFAD mice. (a,b) Confocal images of hippocampal sections from WT, and untreated and VBIT-4-treated 5XFAD mice co-immunostained for p-NF-kB-p65 and TNF-α (**a**) and their relative expression levels (**b**). (i) shows an enlargement of a section to show co-staining of p-NF-kB and TNF-α (arrows). Results show means ± SEM (n=3), ***p* <0.01 ****p* <0.001. *p*-value in blue represents the significance of VBIT-4-treated mice relative to untreated mice. NS, not significant. (**c,d**) Co-immunostaining for p-NF-kB-p65 with GFAP (**c**) or with TUBB3**,b,** arrows) (**d**), showing p-NF-kB expression in astrocytes and neurons in cortical and hippocampal sections from 5XFAD mice. Enlargement are shown in **b**(ii,iii**),** arrows point to co-staining.

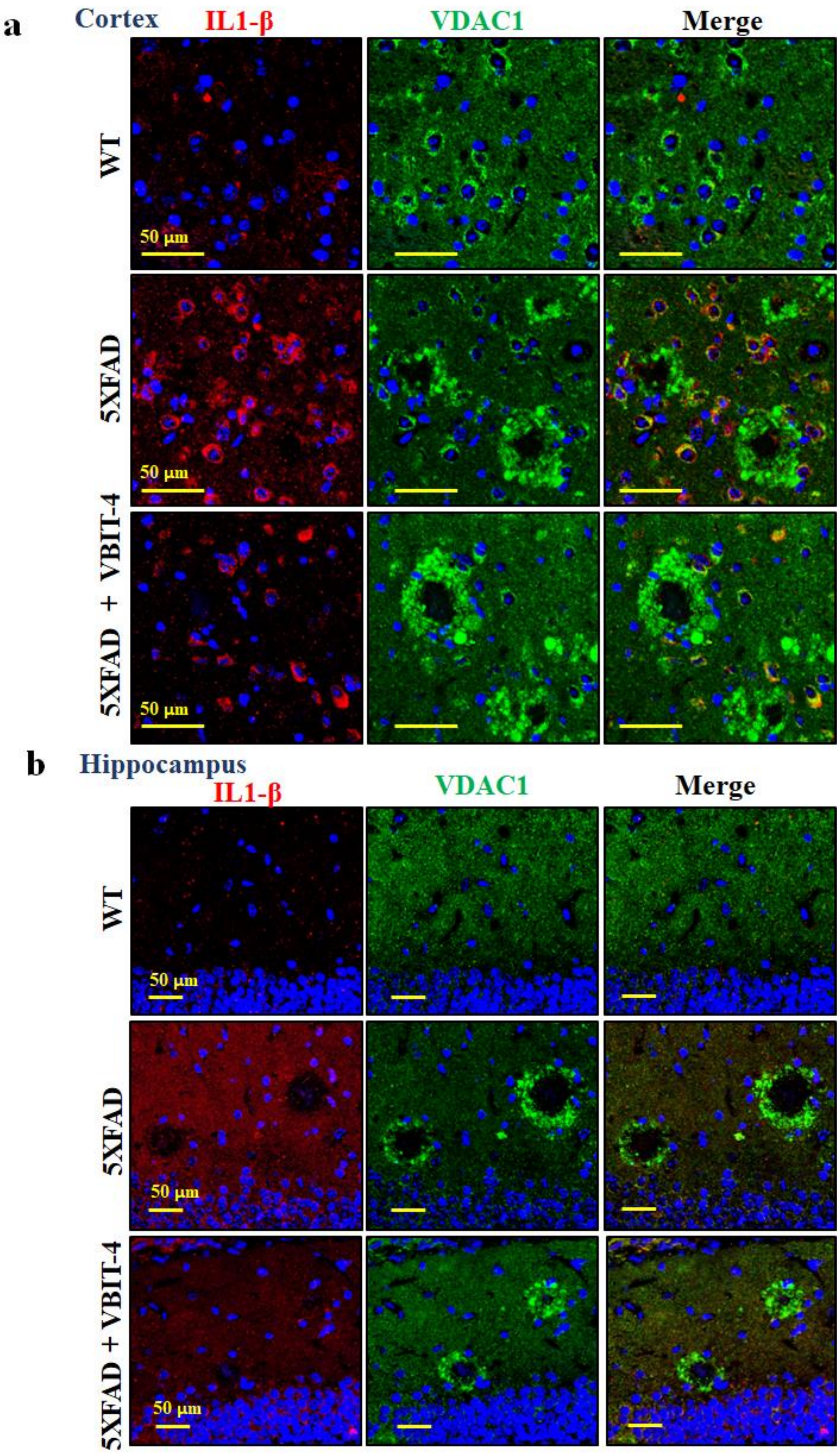

Fig. S15. VBIT-4 reduces IL1-β expression in 5XFAD mice. (a,b) Confocal images of cortical (**A**) and hippocampal (**B**) sections from WT, and untreated and VBIT-4-treated 5XFAD mice, co-immunostained for IL-1β and VDAC1.

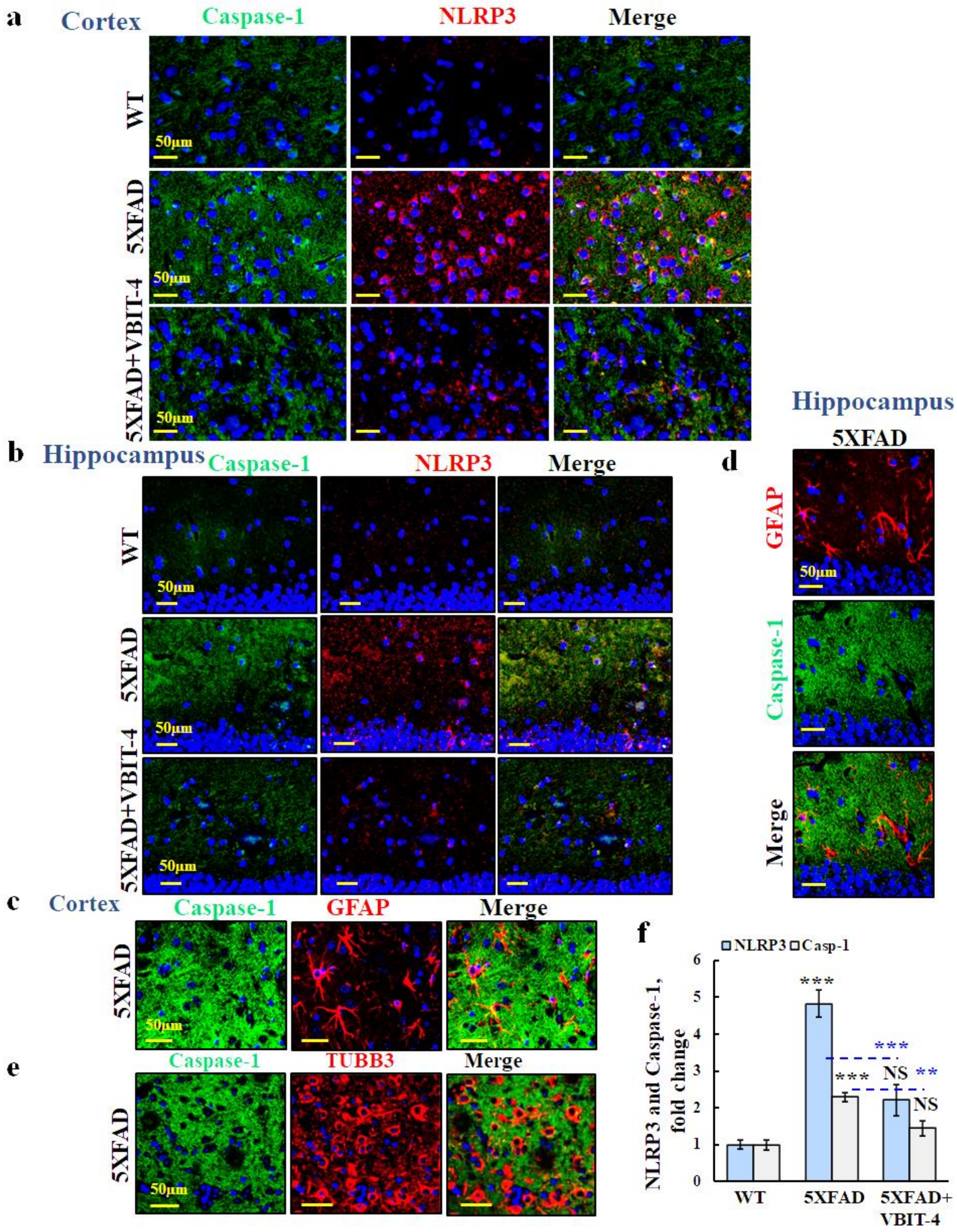

Fig. S16. VBIT-4 reduces NRLP3 and caspase-1 expression in 5XFAD mice. Confocal images of cortical and hippocampal sections from WT, and untreated and VBIT-4-treated 5XFAD mice co-immunostained for caspase-1 and NRLP3 (**a,b**), or GFAP (**c,d**), or TUBB3 (**e**) to visualize their expression in astrocytes or/and neurons. (**f**) Quantitative analysis of NRLP3 and caspase-1 in the hippocampus. Results show means ± SEM (n=3), $**p < 0.01$ $***p < 0.001$. *p*-value in blue color represents the significance of VBIT-4-treated relative to untreated mice. NS, not significant.

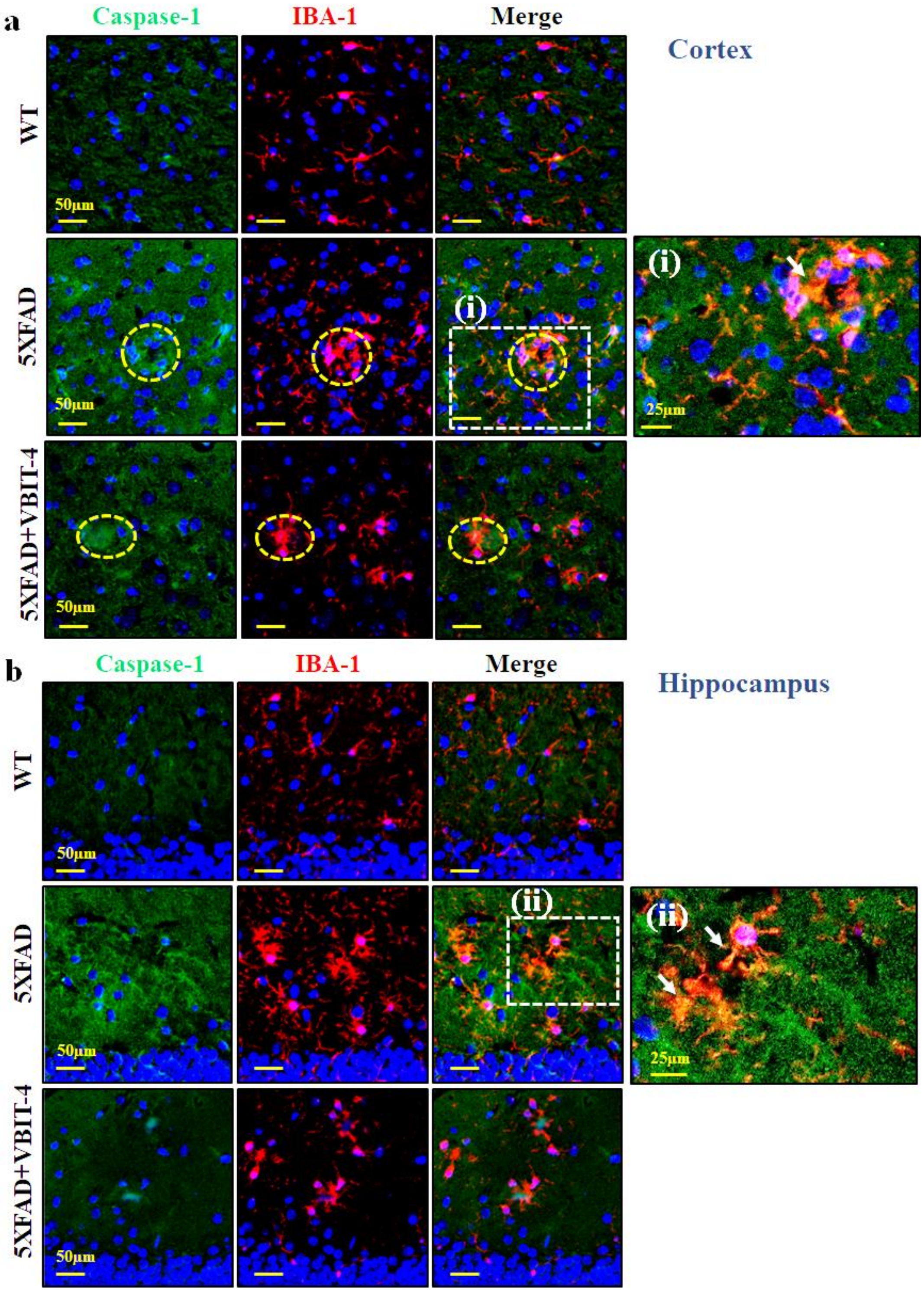

Fig. S17. VBIT-4 reduces IBA-1 and caspase-1 expression in 5XFAD mice. Confocal images of cortical (**a**) and hippocampal (**b**) sections from WT, and untreated and VBIT-4-treated 5XFAD mice co-immunostained for IBA1 and caspase-1. The expression of caspase -1 in the microglia is clearly shown (enlargements, **a(i), b(ii),** arrow**s**). Circles show Aβ-plaques.

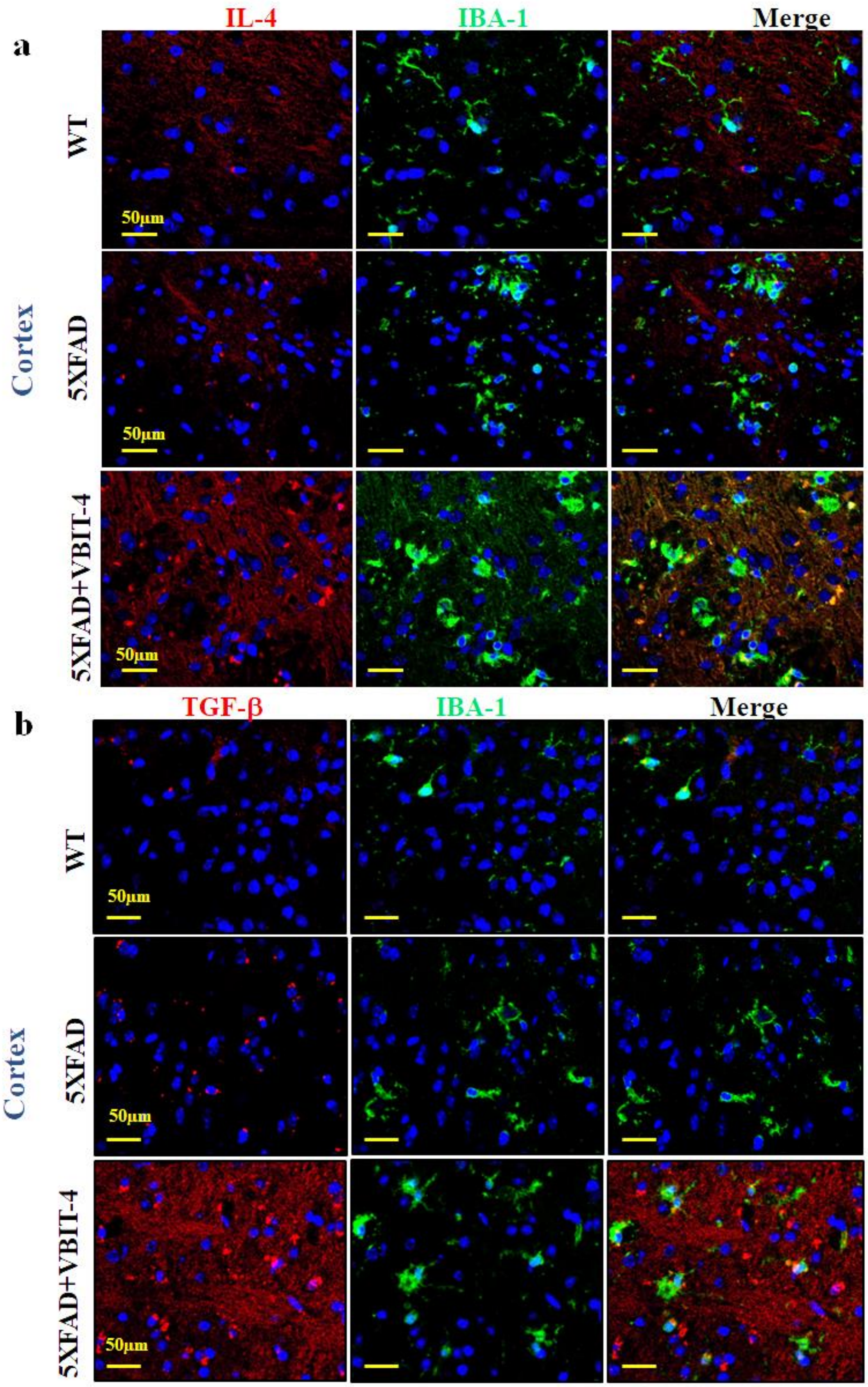

Fig. S18. VBIT-4 increases the expression of IL-4 and TGF-β in microglia of 5XFAD mice. Confocal images of cortical sections from WT, and untreated and VBIT-4-treated 5XFAD mice co-immunostained for IBA-1 and IL-4 (**a**), or TGF-β (**b**).

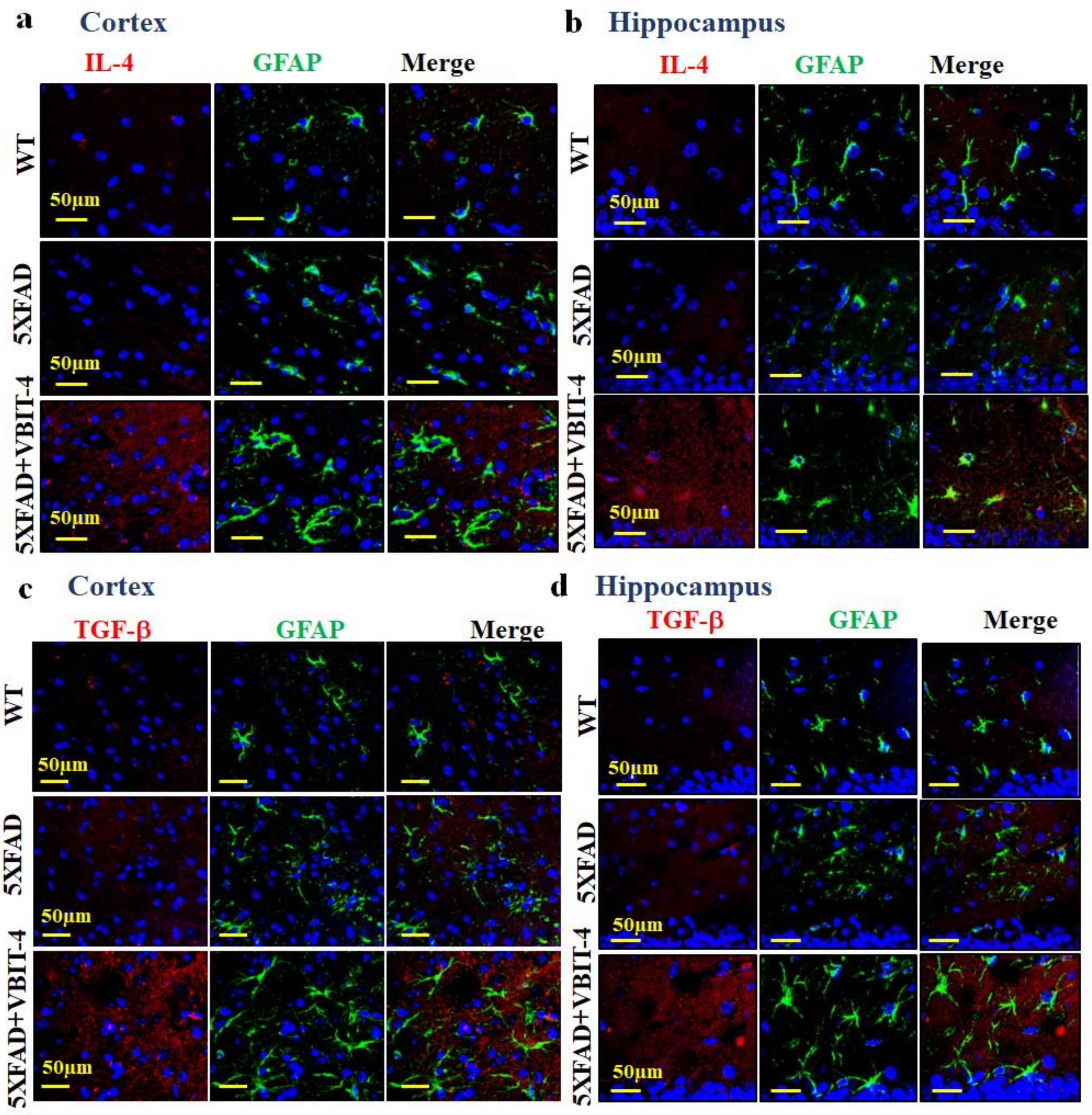

Fig. S19. VBIT-4 increases the expression of IL-4 TGF-β in astrocytes of 5XFAD mice. Cortical (**a,c**) and hippocampal (**b,d**) confocal images from WT, and untreated and VBIT-4-treated 5XFAD mice co-immunostained for GFAP and IL-4 (**a,b**) or TGF-β (**c,d**).
